# A Target-Disease Network Model of Second-Generation BCR-ABL Inhibitor Action in Ph+ ALL

**DOI:** 10.1371/journal.pone.0077155

**Published:** 2013-10-10

**Authors:** Uwe Rix, Jacques Colinge, Katharina Blatt, Manuela Gridling, Lily L. Remsing Rix, Katja Parapatics, Sabine Cerny-Reiterer, Thomas R. Burkard, Ulrich Jäger, Junia V. Melo, Keiryn L. Bennett, Peter Valent, Giulio Superti-Furga

**Affiliations:** 1 CeMM – Research Center, Molecular Medicine of the Austrian Academy of Sciences, Vienna, Austria; 2 Department of Internal Medicine I, Division of Hematology and Hemostaseology, Medical University of Vienna, Vienna, Austria; 3 Ludwig Boltzmann Cluster Oncology, Vienna, Austria; 4 Department of Haematology, Centre for Cancer Biology, Adelaide, Australia; 5 Imperial College London, London, United Kingdom; Westmead Millennium Institute, University of Sydney, Australia

## Abstract

Philadelphia chromosome-positive acute lymphoblastic leukemia (Ph+ ALL) is in part driven by the tyrosine kinase bcr-abl, but imatinib does not produce long-term remission. Therefore, second-generation ABL inhibitors are currently in clinical investigation. Considering different target specificities and the pronounced genetic heterogeneity of Ph+ ALL, which contributes to the aggressiveness of the disease, drug candidates should be evaluated with regard to their effects on the entire Ph+ ALL-specific signaling network. Here, we applied an integrated experimental and computational approach that allowed us to estimate the differential impact of the bcr-abl inhibitors nilotinib, dasatinib, Bosutinib and Bafetinib. First, we determined drug-protein interactions in Ph+ ALL cell lines by chemical proteomics. We then mapped those interactions along with known genetic lesions onto public protein-protein interactions. Computation of global scores through correlation of target affinity, network topology, and distance to disease-relevant nodes assigned the highest impact to dasatinib, which was subsequently confirmed by proliferation assays. In future, combination of patient-specific genomic information with detailed drug target knowledge and network-based computational analysis should allow for an accurate and individualized prediction of therapy.

## Introduction

Philadelphia chromosome-positive (Ph+) leukemias express the oncogenic fusion tyrosine kinase BCR-ABL, which drives the disease through constitutive anti-apoptotic and proliferative signaling. Ph+ leukemias are divided into chronic myeloid leukemia (CML) [1] and a subset of acute lymphoblastic leukemia (ALL) [2]. CML is successfully treated with the BCR-ABL tyrosine kinase inhibitor imatinib (Gleevec, STI-571), which is widely appreciated as the paradigm for targeted therapy [3]. Even though resistance against imatinib is observed in several cases [4], many of these can be adequately addressed through the employment of more potent second-generation BCR-ABL kinase inhibitors, such as nilotinib (*Tasigna*, AMN107) [5], dasatinib (Sprycel, BMS-354825) [6], bosutinib (SKI-606) [7] and bafetinib (INNO-406, NS-187) [8]. In addition, nilotinib and dasatinib have been described to exert superior effects in freshly diagnosed patients with CML [9,10]. Therefore, these agents are expected to replace imatinib as frontline therapy in the near future. 

Tyrosine kinase inhibitors (TKI) have also had a significant impact on the therapy of Ph+ ALL as the introduction of imatinib greatly improved initial responses of patients. Though it is enhanced by combination of imatinib with conventional chemotherapy, remission is short-lived and relapse remains a daunting challenge [11-13]. This is caused by many of the same mechanisms relevant in CML, such as BCR-ABL point mutations that confer resistance to imatinib [14,15]. In addition, in about 20-30% of all CML patients who progress to blast phase, the transformed clone resembles Ph+ ALL (lymphoid blast phase of CML). In both instances, the focus of the medical and research community has turned again towards next-generation TKI [13]. Likewise, combinations of nilotinib and dasatinib with chemotherapy are starting to show some promising results in the treatment of Ph+ ALL [12]. 

Being a more heterogenous disease than CML [16], Ph+ ALL shows on average 8 to 9 gene copy number alterations in addition to the expression of BCR-ABL. The most prominent deletions were observed for the transcription factor genes *IKFZ1* (encoding IKAROS) and *PAX5* as well as for *CDKN2A*, which encodes the tumor suppressor cyclin-dependent kinase inhibitor 2A [17]. Deletion or mutation of *IKZF1* or *CDKN2A* have been described to have a negative prognostic impact [18,19]. Thus, it appears that the particularly aggressive character of Ph+ ALL is not owed to the constitutive tyrosine kinase activity of BCR-ABL alone, but also to the contributions of other genetic factors. Accordingly, given that many kinase inhibitors are known to be highly pleiotropic drugs, it is not clear how effective the second-generation BCR-ABL inhibitors will be in the long-term and which one will be best suited for therapy of treatment-naïve Ph+ ALL with wild-type BCR-ABL. Kinase inhibitor target profiles are routinely investigated on a kinome-wide level either by large-scale *in vitro* kinase inhibition or kinase binding competition assays [20]. For a systems-type appreciation of TKI action, however, it is advantageous to employ a cell-specific approach. At the same time, it should include a genome-, transcriptome-, or proteome-wide dimension. For instance, one method that is widely used determines drug-induced transcriptomic signatures [21]. 

**Figure 1:**
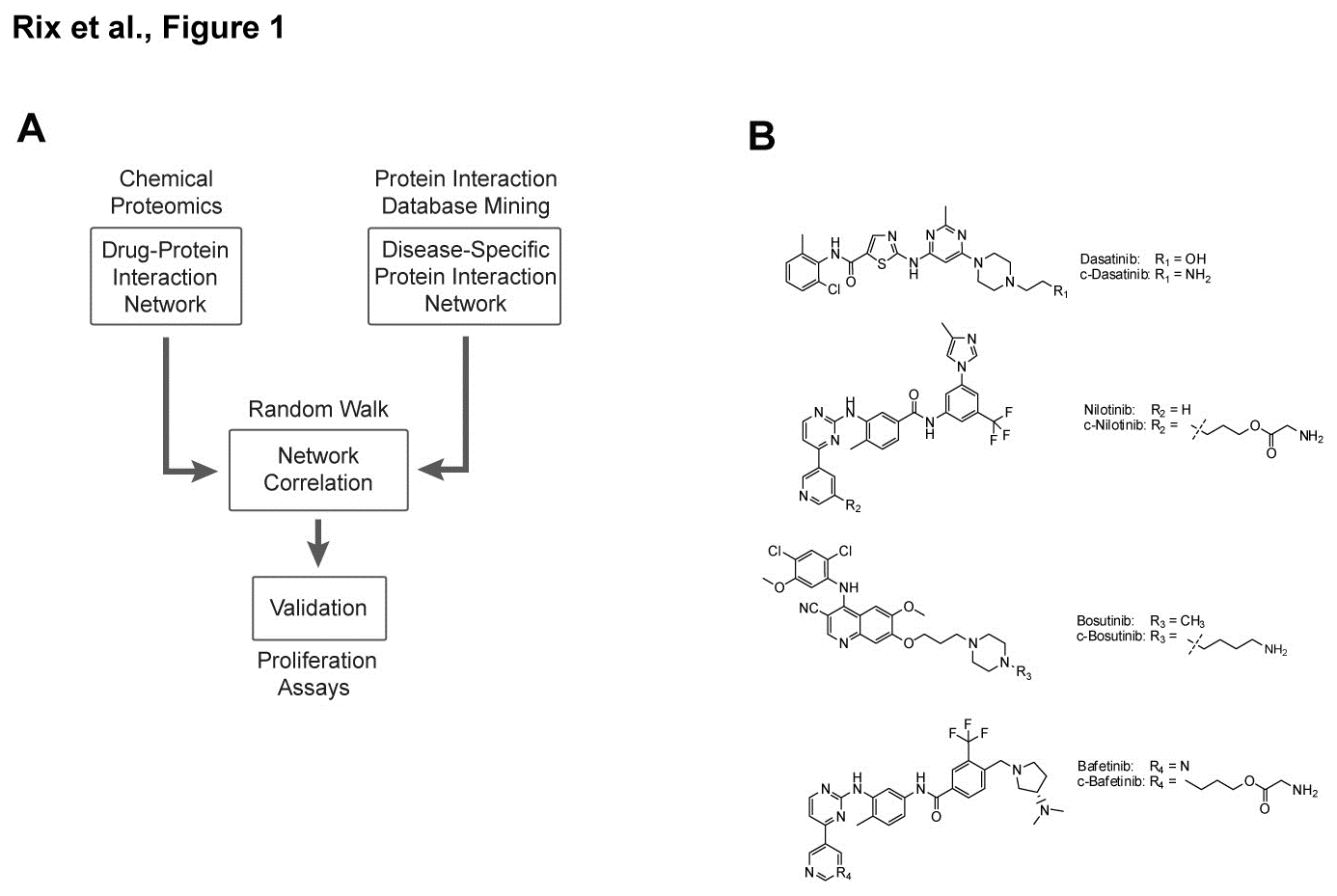
Schematic outline of the integrated chemical proteomics and computational biology strategy. **A**. Drug-protein interaction networks are generated by chemical proteomics while the protein-protein interaction (PPI) network is derived from public databases and modified to represent the specific disease. The interaction networks are correlated through a random walk approach across the PPI network using proteins from the drug-protein network as entry points. The resulting correlation scores are subsequently validated by cell proliferation assays. **B**. Chemical structures of the four second-generation BCR-ABL tyrosine kinase inhibitors dasatinib (Sprycel, BMS-354825), nilotinib (*Tasigna*, AMN107), bosutinib (SKI-606) and bafetinib (INNO-406, NS-187) as well as their coupleable analogues c-dasatinib, c-nilotinib, c-bosutinib and c-bafetinib that were used for immobilization and subsequent chemical proteomics experiments.

Here, we chose a systems biology approach that integrated proteomics and computational methods to predict TKI action in a Ph+ ALL-specific context (Figure 1A). First, we characterized the global protein binding signatures of nilotinib, dasatinib, bosutinib and bafetinib in Ph+ ALL cells by chemical proteomics, an unbiased, post-genomic drug affinity chromatography technology enabled by downstream mass spectrometry (MS) [22-25]. In parallel, we compiled protein-protein interaction (PPI) data from several public databases and generated Ph+ ALL disease-specific PPI network models, which were based on previously reported copy number alterations [17]. Correlation of the obtained drug-target profiles with the Ph+ ALL PPI network models allowed for the correct prediction of dasatinib as the most efficient drug as determined by subsequent validation experiments.

## Materials and Methods

### Biological Material

BV-173 and SUP-B15 were obtained from the German Collection of Microorganisms and Cell Cultures (DSMZ), Z-119 cells were a kind gift from the originator Dr. Zeev Estrov (MD Anderson Cancer Center). BV-173, Z-119, and SUP-B15 cells were cultured in RPMI 1640 medium and 10% (SUP-B15: 20%) fetal calf serum (PAA laboratories, Pasching, Austria). Peripheral blood was collected at diagnosis from six patients with Ph+ ALL. For molecular characteristics of patient samples see Table S1 in File S1. Mononuclear blood cells (PBMCs) were obtained using Ficoll and subsequently pooled. The study was conducted in accordance with the Declaration of Helsinki and was approved by the institutional review board (Medical University of Vienna). Written informed consent was obtained before blood donation in each case. Antibodies used were mouse monoclonal anti-phosphotyrosine 4G10 (Upstate Biotechnology), monoclonal mouse anti-c-ABL (Ab-3) (Calbiochem), rabbit polyclonal anti-actin (Cytoskeleton), IRDye 800 donkey anti-rabbit IgG (LI-COR Biosciences) and peroxidase-conjugated AffiniPure goat anti-mouse IgG (Jackson Immuno Research Laboratories).

### Compounds, Immobilization and Affinity Purification

Nilotinib and dasatinib were purchased from LC Laboratories (Woburn, MA). Bosutinib was a kind gift of Oridis Biomed (Graz, Austria). Bafetinib, pc-bafetinib, c-dasatinib and pc-nilotinib were synthesized by WuXi AppTec (Shanghai, China). c-Bosutinib was synthesized by Vichem Chemie Ltd. (Budapest, Hungary). In analogy to c-bafetinib, pc-nilotinib was esterified with N-Boc-glycine, deprotected with trifluoroacetic acid and the resulting c-nilotinib was immobilized on NHS-activated Sepharose 4 Fast Flow resin (GE Healthcare Bio-Sciences AB, Uppsala, Sweden) [26]. c-Dasatinib, c-bosutinib and c-bafetinib were immobilized as reported previously. However, the final drug concentration was 25 nmol/50 µl beads for all four drugs [26-28]. Affinity chromatography and elution with formic acid were performed in biological duplicate as described [29]. Modifications included the reduction of the incubation period from 2 hrs to 1 hr as well as omittance of *N*-dodecyl-β-D-maltoside from the lysis buffer. The HEPES-NaOH buffer was composed of 50 mM HEPES (pH 8.0), 0.5 mM EDTA and 100 mM NaCl. After incubation, beads were washed with 100 bed volumes of lysis buffer and subsequently with 50 bed volumes of HEPES-NaOH buffer. Competition experiments were performed in singlicate by incubation of an aliquot of each cell lysate with the respective drug affinity matrix in the presence of 20 µM free drug. For BV-173 lysates, we applied 5 mg, for Z-119 lysates 4.5 mg of total protein per experiment at a concentration of 5 mg/300 µl. For the patient PBMC pool-derived lysate we applied 0.69 mg of total cell lysate. All mass spectrometry experiments were performed at least in duplicates.

### Sample Preparation, Mass Spectrometry (MS) and MS Data Processing

Preparation of samples, MS analysis and data processing were performed as reported [29]. In addition, the sequence of BCR-ABL^p190^ was appended to our in-house database prior to database searching with Mascot (Matrix Science, London, UK) and Phenyx (GeneBio S.A., Geneva, Switzerland) [30] to account for the unique BCR-ABL isoform observed in Ph+ ALL. Peptide and protein identifications obtained by both search engines were combined with score thresholds chosen to achieve a maximal false discovery rate (FDR) of 1% on the protein groups (proteins sharing peptides). Namely, single peptide protein identifications were accepted provided Mascot ion score ≥ 40 or Phenyx z-score ≥ 4.75 and P-value < 0.001. Multiple peptide protein identifications were imposed Mascot ion score ≥ 14 or Phenyx z-score ≥ 4.2 and P-value < 0.001. For a protein passing the criteria above, all the additional peptides with Mascot ion score ≥ 10 or Phenyx z-score ≥ 4 were additionally accepted and taken into account in the protein group 1% FDR estimation. Conflicting spectral identifications between the two search engines were discarded for security and as a practical way to validate the FDR on a routine basis. 

### Kinase Inhibition Analysis

Nilotinib and non-esterified c-nilotinib (pc-nilotinib) were assayed *in vitro* for inhibition of recombinant full-length c-ABL (Upstate Biotechnology, Lake Placid, NY) as described previously [26]. *In vitro* kinase inhibition for MAP2K1, MAP2K2, MAPK9 and binding assays for MAP3K2 were performed on the Invitrogen Z’LYTE® or LanthaScreen® platforms, respectively.

### Target Deconvolution Analysis

Specificity of protein binding was determined by differential analysis of competition pulldowns. Based on the spectral count (SC) ratio between uncompeted and competed experiments, a threshold of 2.0 and a minimum average spectral count of 10.0 were applied. For proteins not identified in the competition experiment, a minimum average spectral count of 1.0 was required to be considered specific. Additionally, proteins absent in at least two drug-protein interaction datasets, thus suggesting specificity, were included. Subsequently, a score *A*_*i*_ = *SC*_*i*_ * SeqCov_*i*_ was computed for each specific protein *i* to describe apparent abundance in the eluate, which correlates with binding strength and protein expression (*SC*_*i*_=spectral count, *SeqCov*_*i*_=protein sequence coverage). For combining the two cell line-dependent datasets, the maximum of both scores was applied to represent the optimal binding potential. Non kinase target scores were reduced by the application of a factor 0.25 to account for their very likely secondary binder nature.

### Human protein-protein interaction network

This PPI network was obtained by integrating data found in the IntAct, MINT, BioGRID, HPRD, and DIP databases as of October 2011. All the protein identification codes were mapped to UniProtKB accession codes. The resultant PPI network contained 13461 distinct proteins and 90363 pairwise interactions. We performed subsequent network computations restricting to the largest connected component that was comprised of 13350 distinct proteins and 90292 interactions. 

### Cell Proliferation Assays

Cells were placed into 96 well plates (1x10^5^ cells/well) and incubated in RPMI 1640 medium in the presence or absence of various concentrations of kinase inhibitors at 37°C for 48 hours. Then, ^3^H-thymidine (0.5 µCi/well) was added. After twelve hours, plates were recovered, frozen and stored at -20°C until use. After thawing, cells were harvested on filter membranes (Packard Bioscience, Meriden, CT) in a Filtermate 196 harvester (Packard Bioscience). Filters were air-dried, and the bound radioactivity was measured in a beta-counter (Top-Count NXT, Packard Bioscience). All experiments were performed in triplicates.

### Target specificity P-values

Comparing the target profiles of the 4 compounds in BV-173 and Z-119 cells we reported specific, significant identifications of kinases for a cell type or a compound. In every case, the reported P-values was computed exploiting the biological and technical replicates available (4 in total for each compound in the cell lines; 2 for dasatinib in the patient pool). We computed the abundance score for each replicate and compared the resulting values with a (non parametric) one-sided Kolmogorov-Smirnov test. We only performed “coherent” comparisons in the tests. For instance, assessing FGR presence in dasatinib patient pulldowns we compared the 2 values available for these samples with the 8 values, i.e. 0, available for dasatinib in the cell lines (data for the other drugs were not used). Similarly, assessing CDK specificity for nilotinib, we compared the 8 values available for this compound in the 2 cell lines with the 24 values (3 drugs, quadruplicates, 2 cell lines) corresponding to the other drugs (again 0 in this example).

## Results

### Cellular target profiling by chemical proteomics identifies 79 protein kinases binding to dasatinib, nilotinib, bosutinib, and bafetinib in Ph+ ALL cells

Dasatinib, nilotinib, bosutinib, and bafetinib were chosen, since they were engaged in clinical trials in various phases or used in practice for Ph+ ALL when this study was initiated. In order to perform a comprehensive comparison of these TKI in this specific context, we investigated two widely used human BCR-ABL-positive ALL cell lines, BV-173 and Z-119. While Z-119 expresses the BCR-ABL^p190^ isoform, which accounts for approximately two thirds of Ph+ ALL cases, BV-173 features the BCR-ABL^p210^ isoform that is found in the remaining Ph+ ALL patient population, as well as in CML [11]. We further collected PBMCs from six Ph+ ALL patients at diagnosis (Table S1 in File S1), which due to the small overall sample size available were only used for experiments with dasatinib. Although not included in later computations, this experiment provides an appreciation of the degree of similarity between target profiles from cell lines and Ph+ ALL patients. In a next step, we determined the cell-specific target profiles of the four TKI by immobilizing previously established and validated drug analogues as bait molecules and performing subsequent shotgun chemical proteomics (Figure 1) [26-29]. For optimal comparability, we also generated a coupleable nilotinib analogue (c-nilotinib) (Figure 1B; Figure S1A in File S1), which should bind to the ABL kinase domain in a fashion similar to the other compounds with the linker moiety protruding into solvent space from the hinge region [31,32]. In *in vitro* ABL kinase activity assays, pc-nilotinib proved to be as potent as nilotinib itself supporting its suitability as a probe compound (Figure S1B in File S1).

This target profiling analysis showed 79 kinases to bind to the four drugs across all cell types examined (Table 1; Tables S2-S4 in File S1). In spite of significant overlap of the observed profiles there were a number of notable differences, particularly between the various drugs, but also between the cell types, which were likely due to differences in the expression patterns. Thus, some kinases were exclusively observed in the patient pool (e.g. FGR, P<0.041 see Materials and Methods), in the Z-119 (e.g. TESK2, P<0.019) or in the BV-173 cells (e.g. CSNK2A1, P<0.0012; CSNK2A2, P<0.0022; EPHB3, P<0.00034). Among the four drugs tested, nilotinib was most selective displaying 19 kinase targets, while bosutinib was most promiscuous showing 54 kinase targets. BCR-ABL was identified with all four drugs albeit to different extent, which reflected known differences in affinity for BCR-ABL [5-8]. Only few other kinases interacted with all four drugs. These included the mixed lineage kinase MLTK (ZAK) and the SRC family kinases (SFK) LYN and LCK, which were expected with the pan-SFK inhibitors dasatinib and bosutinib. However, they also have been associated previously with bafetinib and nilotinib [26,33,34]. Conversely, many kinases were interacting only with individual drugs. Most noteworthy, as they have not been identified in CML cell lines [24,26-28], were PDGFRB (P<6.2E-6), ILK (P<6.2E-6), and TESK1 (P<0.035) as specific targets of dasatinib, and CHEK2 (P<0.0012), MAP2K1 (P<6.2E-6), and MAP2K2 (P<6.2E-6) specific for bosutinib, CDK9 (nilotinib, P<0.0093) and MAPK8, MAPK9 and MAPK10 (bafetinib and nilotinib, all 3 P<2.59E-5). In addition, MAP3K2, which was found binding with dasatinib and bosutinib, appeared as stronger bosutinib target in Z-119 and BV-173 cells (P<0.019). The majority of the observed kinases have been validated previously (Figure S2 in File S1). For instance, PDGFRB, ILK, TESK1, CHEK2, MAP2K1, MAP2K2, MAP3K2 and MAPK8 are known targets of the respective drugs. We also determined the IC_50_ value of nilotinib for MAPK9 by *in vitro* kinase assays to be 12.9 μM thereby validating this interaction. The relatively weak IC_50_’s for MAP2K1 (bosutinib) and MAPK9 (nilotinib) suggest that the strong enrichment in our proteomics analysis may in part be due to high protein abundance and/or co-purification of these kinases with their known interaction partners MAP2K2 and MAPK8, which are much stronger inhibited by bosutinib and nilotinib, respectively [34].

**Table 1:** Protein kinase targets of dasatinib, nilotinib, bosutinib and bafetinib in Ph+ ALL cells.

		**Dasatinib**			**Nilotinib**		**Bosutinib**		**Bafetinib**	
**Gene name**	**AC**	**BV-173**	**Z-119**	**Patient Pool**	**BV-173**	**Z-119**	**BV-173**	**Z-119**	**BV-173**	**Z-119**	
AAK1	Q2M2I8	-/-	-/-	-/-	-/-	-/-	21/34	27/40	-/-	-/-	
ABL1	P00519	34/42	(15/17)	2/3	3/3	-/-	27/33	(11/14)	20/25	(3/3)	
ABL2 (ARG)	P42684	25/29	16/20	-/-	-/-	-/-*	16/21	8/9	13/16	4/4	
BCR-ABL	N/A	66/42	22/13	(2/2)	7/4	-/-*	56/35	15/10	39/25	4/2	
BLK	P51451	14/29	10/22	-/-	-/-	-/-	7/17	3/6	3/6	-/-	
BMP2K (BIKE)	Q9NSY1	-/-	-/-	-/-	-/-	-/-	22/26	21/24	-/-	-/-	
BTK	Q06187	43/66	43/70	19/36	13/21	7/10	40/65	36/59	11/19	-/-	
CABC1 (ADCK3)	Q8NI60	11/21	5/9	9/16	-/-	-/-	-/-	-/-	-/-	-/-	
CAMK2B	Q13554	-/-	-/-	-/-	2/6	-/-	8/26	-/-	-/-	-/-	
CAMK2D	Q13557	-/-	-/-	-/-	4/11	-/-	24/54	-/-	-/-	-/-	
CAMK2G	Q13555	-/-	-/-	-/-	2/6	-/-	18/42	13/30	-/-	-/-	
CDK5	Q00535	-/-	-/-	-/-	2/7	-/-	-/-	-/-	-/-	-/-	
CDK9	P50750	-/-	-/-	-/-	5/15	2/7	-/-	-/-	-/-	-/-	
CDK13 (CDC2L5)	Q14004	2/1	2/1	2/1	-/-	-/-	-/-	-/-	-/-	-/-	
CDK18 (PCTK3)	Q07002	-/-	2/5	-/-	-/-	-/-	-/-	-/-	-/-	-/-	
CHEK2	O96017	-/-	-/-	-/-	-/-	-/-	6/13	9/21	-/-	-/-	
CSK	P41240	26/65	16/41	12/30	11/31	6/15	16/41	10/29	9/25	-/-	
CSNK2A1	P68400	8/31	-/-	-/-	-/-	-/-	5/17	-/-	2/5	-/-	
CSNK2A2	P19784	4/19	-/-	-/-	-/-	-/-	3/12	-/-	-/-	-/-	
EPHB3	P54753	8/10	-/-	-/-	-/-	-/-	5/6	-/-	-/-	-/-	
EPHB4	P54760	22/27	27/36	-/-	-/-	-/-	25/35	22/30	-/-	-/-	
FER	P16591	-/-	-/-	-/-	-/-	-/-	14/17	6/7	-/-	-/-	
FES	P07332	-/-	-/-	-/-	-/-	-/-	-/-	2/3	-/-	-/-	
FGR	P09769	-/-	-/-	5/13							
FLT3	P36888	-/-	2/2	-/-	-/-	-/-	-/-	-/-	-/-	-/-	
FRK	P42685	2/3	2/3	-/-	-/-	-/-	2/3	2/3	-/-	-/-	
FYN	P06241	12/25	15/29	-/-	-/-	-/-	12/25	10/20	2/3	-/-	
GAK	O14976	24/23	19/17	4/4	-/-	-/-	40/39	33/32	-/-	-/-	
HCK	P08631	8/16	4/6	3/6	2/3	-/-	10/21	5/9	2/3	2/3	
IKBKE	Q14164	-/-	-/-	-/-	-/-	-/-	13/23	2/3	-/-	-/-	
ILK	Q13418	15/36	20/45	13/25	-/-	-/-	3/6	-/-	-/-	-/-	
IRAK3	Q9Y616	-/-	3/7	-/-	-/-	-/-	-/-	-/-	-/-	-/-	
IRAK4	Q9NWZ3	2/5	4/9	-/-	-/-	-/-	6/13	8/18	-/-	-/-	
KDR (VEGFR2)	P35968	-/-	2/1	-/-	-/-	-/-	-/-	-/-	-/-	-/-	
LCK	P06239	17/47	14/36	6/14	5/11	-/-	15/39	13/32	14/36	7/18	
LIMK2	P53671	8/14	3/6	-/-	-/-	-/-	-/-	-/-	-/-	-/-	
LYN	P07948	29/65	31/65	11/25	9/19	2/4	29/63	27/65	17/39	16/33	
MAP2K1 (MEK1)	Q02750	-/-	-/-	-/-	-/-	-/-	15/39	13/37	-/-	-/-	
MAP2K2 (MEK2)	P36507	-/-	-/-	-/-	-/-	-/-	19/44	16/46	-/-	-/-	
MAP2K5	Q13163	-/-	-/-	-/-	-/-	-/-	5/13	-/-	-/-	-/-	
MAP3K1	Q13233	15/12	-/-	-/-	-/-	-/-	21/19	7/5	-/-	-/-	
MAP3K2	Q9Y2U5	6/10	4/6	2/3	-/-	-/-	15/26	14/26	-/-	-/-	
MAP3K3	Q99759	5/9	2/3	-/-	-/-	-/-	9/16	9/15	-/-	-/-	
MAP4K1 (HPK1)	Q92918	-/-	-/-	-/-	-/-	-/-	13/18	9/13	-/-	-/-	
MAP4K2 (GCK)	Q12851	-/-	-/-	-/-	-/-	-/-	22/35	10/16	-/-	-/-	
MAP4K3	Q8IVH8	-/-	-/-	-/-	-/-	-/-	3/4	-/-	-/-	-/-	
MAP4K4	O95819	-/-	-/-	-/-	-/-	-/-	6/5	4/3	-/-	-/-	
MAP4K5 (KHS)	Q9Y4K4	13/16	11/15	-/-	-/-	-/-	23/33	20/26	-/-	-/-	
MAPK8 (JNK)	P45983	-/-	-/-	-/-	17/56	13/41	-/-	-/-	5/12	3/8	
MAPK9 (JNK2)	P45984	-/-	-/-	-/-	16/43	13/35	-/-	-/-	8/24	5/12	
MAPK10 (JNK3)	P53779	-/-	-/-	-/-	10/20	-/-	-/-	-/-	4/7	-/-	
MAPK14 (p38a)	Q16539	14/48	16/64	8/18	13/48	11/42	-/-	3/7	14/51	15/57	
MAPKAPK2	P49137	-/-	-/-	-/-	-/-	-/-	-/-	-/-	2/6	-/-	
MAPKAPK3	Q16644	-/-	-/-	-/-	3/10	-/-	-/-	-/-	6/23	2/5	
MLKL	Q8NB16	-/-	-/-	3/8							
MLTK (ZAK)	Q9NYL2	19/29	12/17	-/-	11/17	7/10	15/23	10/14	16/25	11/16	
MST4	Q9P289	-/-	-/-	-/-	-/-	-/-	5/17	5/17	-/-	-/-	
PDGFRB	P09619	16/16	25/25	-/-	-/-	-/-	-/-	-/-	-/-	-/-	
PKMYT1	Q99640	11/31	5/14	-/-	-/-	-/-	11/31	10/29	-/-	-/-	
PRKAA1 (AMPK1)	Q13131	-/-	-/-	-/-	-/-	-/-	9/18	11/24	-/-	-/-	
PTK2 (FAK)	Q05397	-/-	-/-	-/-	-/-	-/-	16/17	7/8	-/-	-/-	
PTK2B (PYK2)	Q14289	-/-	-/-	-/-	12/14	5/5	18/22	22/27	24/30	20/23	
RIPK2	O43353	17/37	12/25	-/-	-/-	-/-	-/-	-/-	-/-	-/-	
SGK223	Q86YV5	12/11	-/-	-/-	-/-	-/-	8/7	-/-	-/-	-/-	
SIK2 (QIK)	Q9H0K1	11/15	5/6	-/-	-/-	-/-	8/10	2/2	-/-	-/-	
SIK3 (QSK)	Q9Y2K2	-/-	5/5	-/-	-/-	-/-	-/-	-/-	-/-	-/-	
SLK	Q9H2G2	-/-	-/-	-/-	-/-	-/-	19/18	16/12	-/-	-/-	
SRC	P12931	11/23	11/24	-/-	-/-	-/-	10/21	9/19	-/-	-/-	
STK10 (LOK)	O94804	-/-	-/-	-/-	-/-	-/-	26/30	19/23	-/-	-/-	
STK24 (MST3)	Q9Y6E0	-/-	-/-	-/-	-/-	-/-	-/-	3/8	-/-	-/-	
STRADA (LYK5)	Q7RTN6	2/3	-/-	-/-	-/-	-/-	-/-	-/-	-/-	-/-	
SYK	P43405	-/-	-/-	2/3							
TBK1	Q9UHD2	-/-	-/-	-/-	-/-	-/-	16/26	15/24	-/-	-/-	
TEC	P42680	8/14	17/29	2/3	-/-	-/-	-/-	-/-	-/-	-/-	
TESK1	Q15569	5/8	-/-	-/-	-/-	-/-	-/-	-/-	-/-	-/-	
TESK2	Q96S53	-/-	3/6	-/-	-/-	-/-	-/-	-/-	-/-	-/-	
TYK2	P29597	3/2	-/-	-/-	-/-	-/-	-/-	-/-	-/-	-/-	
ULK3	Q6PHR2	-/-	-/-	-/-	-/-	-/-	14/31	9/21	-/-	-/-	
YES1	P07947	13/26	15/29	-/-	-/-	-/-	9/15	10/18	2/3	-/-	

* The presence of BCR-ABL and ABL2 in the Z-119 c-nilotinib eluate was confirmed by quantitative mass spectrometry across the four different drugs using 4-plex isobaric tag for relative and absolute quantitation (iTRAQ) (data not shown).

Values in front of the slash indicate the number of unique peptides identified, values following the slash represent the overall amino acid sequence coverage in percent (individual values for all the replicates available are reported in Tables S2-S4 in File S1). Values in parenthesis indicate that this protein was unambiquously identified in another cell type used in this study, but that it was grouped in this cell type with a different reporter protein that shares the same peptides, but displays in total more peptides. Therefore, it cannot be decided by MS, if the protein is present in this sample or not. AC: SwissProt accession code.

### Differential drug profile analysis reveals 144 specific drug-binding proteins and distinct protein complexes

**Figure 2:**
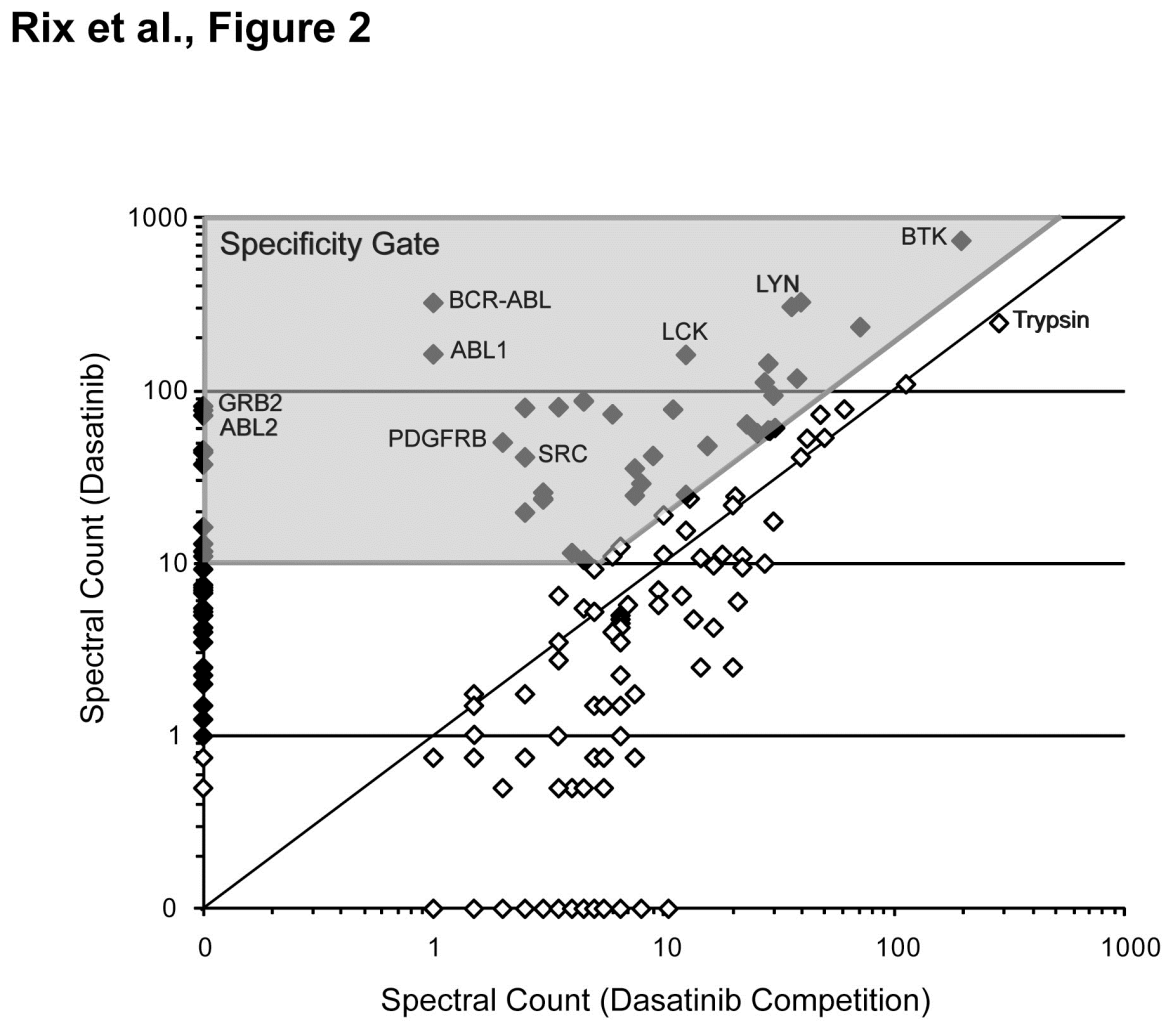
Graphical representation of binding specificity assessment. Using the example of dasatinib and BV-173 cells, the average spectral counts obtained from chemical proteomics were compared with the respective competition experiments in the presence of 20 µM free drug in a double-logarithmic plot. Specific ( ♦) and non-specific (◊) binders were identified by definition of a specificity gate (grey area) with a ratio threshold of 2 and a minimum average spectral count of 10. For proteins that were not identified in the competition experiment, the minimum average spectral count was lowered to 1.

**Figure 3:**
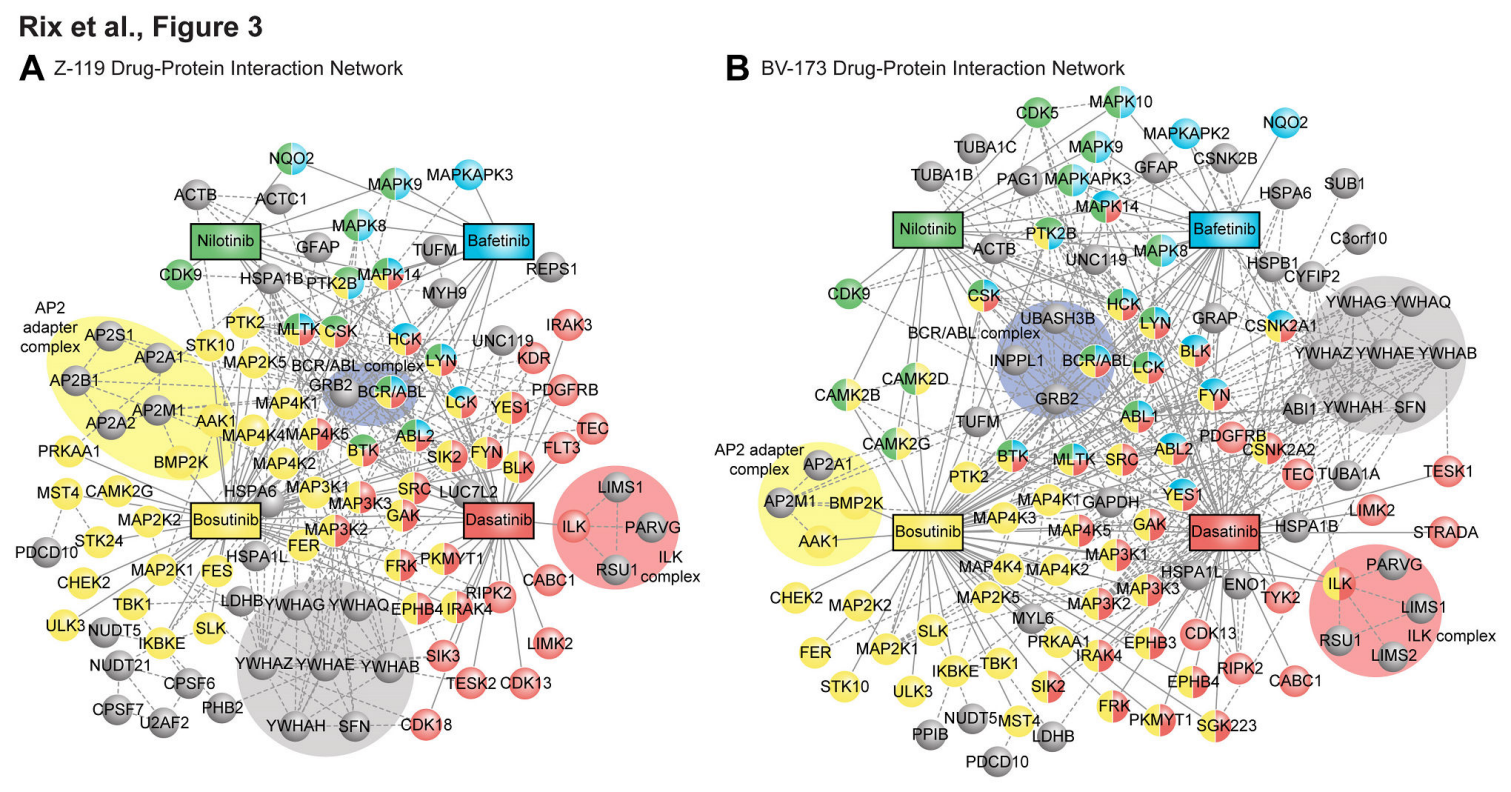
Hybrid drug-protein/protein-protein interaction networks of specific drug binding proteins. Individual cellular target profiles of nilotinib (green), dasatinib (red), bosutinib (yellow) and bafetinib (blue) were intersected with each other and overlaid with PPI data from public databases. Protein kinases and the oxidoreductase NQO2, as a validated target of nilotinib and to lesser extent of bafetinib, were considered to be direct drug binders (solid lines) and color-coded according to the drug they were interacting with. Shared kinase targets display a split color code. All other non-kinase proteins were assumed to be indirect binders (dashed lines) and displayed in grey. The analysis reveals distinct protein complexes, which are enriched by particular drugs and which are highlighted with the respectively colored background. **A**. Z-119 drug-protein interaction network. **B**. BV-173 drug-protein interaction network.

For a proteome-wide understanding of the respective drug-protein interaction networks, we broadened our analysis by including non-kinase targets. Therefore, we performed drug affinity chromatography experiments in the presence of soluble drug (Tables S5 and S6 in File S1), which competes with the respective drug matrix for specific targets and their interaction partners while non-specific proteins remain unaffected. Next, we compared the average spectral counts of regular (uncompeted) and competition experiments and determined proteins that were specific for each drug (Figure 2). Proteins, which were not sufficiently competed by the respective soluble drug, but were otherwise specific with regard to other TKI, were rescued for this analysis. We thus identified 144 proteins, including the 79 mentioned kinases, that were specifically binding to any one or more given TKI in BV-173 and Z-119 cells. Assuming that protein kinases were direct drug binders, we generated hybrid drug-protein/protein-protein interaction networks for each cell type with these 144 proteins by mining of publicly available PPI databases (IntAct, HRPD, MINT, BioGRID, DIP) (Figure 3, Figure S3 in File S1). The majority of non-kinase proteins displayed at least one known interaction with another selected protein, which might serve as validation of the specificity assessment and support the assumption that these proteins are indirect drug binders. To confirm this, we considered that true indirect drug interactors should be in interaction with affinity-enriched kinases more frequently than random. Considering the targets we could map on the PPI network only, the 75 mapped kinases featured 4020 direct interactions. Forty-four of the 63 mapped non-kinases were among the kinase interactors. Given that the interactome contained 13350 proteins, we found a hypergeometric P-value of 2.57e-7. 

This analysis also revealed several distinct protein complexes, such as the well characterized integrin-linked kinase (ILK) complex [35], which interacted with dasatinib. We also prominently observed several members of the AP2 adapter complex [36,37], which was specifically enriched by bosutinib (Figure 3A). A noteworthy protein complex involved BCR-ABL and the adaptor protein GRB2, the phosphatidylinositol-5-phosphatase INPPL1 (SHIP-2) and the tyrosine phosphatase UBASH3B (STS-1), which have been previously described in CML (Figure 3B) [37]. Thus, we were able to capture relevant PPIs underlining the appropriateness to incorporate this information into the network model.

### Correlating drug targets and genetic alterations

In order to estimate protein abundance in the drug eluates and thus the impact of the individual, cell-specific drug-protein interactions in the chemical proteomics analyses, we applied an eluate abundance score *A* based on the product of the observed amino acid sequence coverage and the number of spectral counts for each identified specific protein (Tables S7 and S8 in File S1) [38]. The *A* score is similar to other global abundance scores, in particular emPAI [39], that demonstrated reasonable correlation with true abundance. It allows for a semi-quantitative comparison of proteins within and across samples independent of size, enzymatic function or if they are direct or indirect drug binders. 

**Figure 4:**
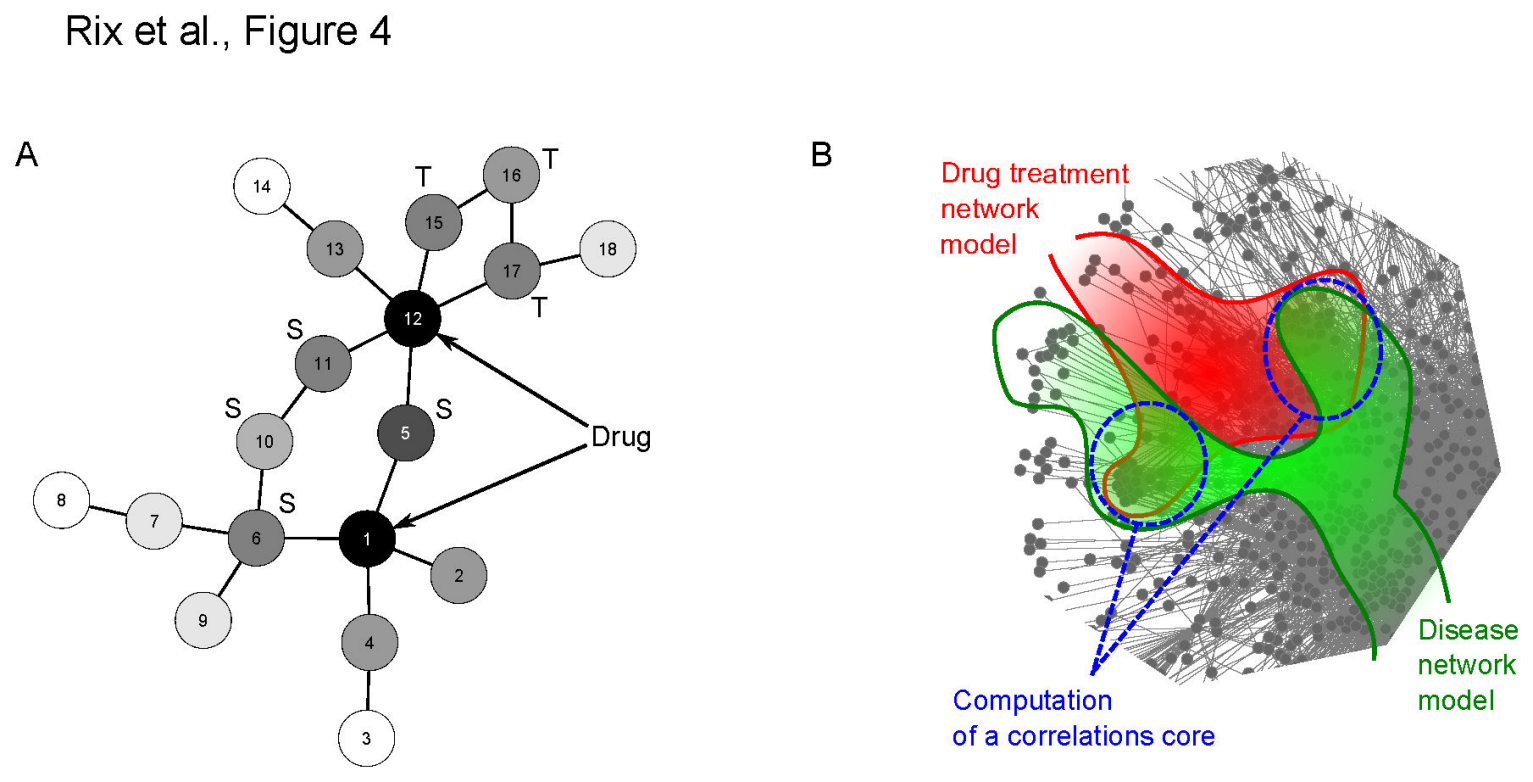
Diffusion and scoring methods. **A**. Schematic representation of a diffusion process in a “toy” network. The proteins (nodes) labeled 1 and 12 are drug targets (in practice they could have distinct weights based on the abundance score but here they have the same for simplicity). After completion of the diffusion process, the entire network is assigned probabilities (black=maximum, white=minimum). Nodes close to the targets typically receive higher probabilities and, due to the network topology, synergistic effects are obtained for nodes close to several targets (marked as “S”) or linked in multiple ways to a single target (marked as “T”). **B**. Principle of scoring illustrated in part of the network: two diffusion processes are performed separately on the PPI network (partially featured): one yields a model of the drug treatment effect (red) and the second one yields a model of the influence of the disease (green). Combining the two set of scores allows for the computation of a correlation score (blue) that measures the adequacy of a drug treatment for a disease.

Given a drug and a set of protein targets, we determined a drug treatment model over the human PPI network by means of a diffusion process. Briefly, the so-called drug treatment model is a probability distribution over all the proteins (nodes) of the PPI network and these probabilities account for the likelihood of being influenced by the drug treatment. To compute the latter probability distribution the drug targets were mapped on the PPI network and assigned initial probabilities proportional to the abundance score *A* defined above. Non drug targets were assigned an initial probability of 0. A diffusion process propagated those initial probabilities to the entire network integrating direct protein interactions of the drug targets and the global network topology (interactions between non targets) (Figure 4A, S1 in File S2). The diffusion was implemented as a modified random walk with restart [40,41]. The initial probabilities were written as a vector *x*_0_ (one component for each protein in the PPI network) and the diffusion was computed by the iteration *x*_i+1_=*(*1-α)*Px*_*i*_+*αx*_0_, where *P* was a diffusion matrix derived from the PPI topology, α was set to 0.3, and the iteration was run until convergence. The limit (asymptotic) probability distribution, covering the whole PPI network, defined the treatment network model. This procedure has been shown to efficiently associate proteins to functionally related other proteins [42,43] and it has the potential to capture synergistic effects arising from multiple targets of a single compound, which is a desirable characteristic for promiscuous small molecules such as the four kinase inhibitors considered here, Additional details, precise mathematical definitions, and proof of convergence are provided in Supplementary Methods (Figures S2-S3 in File S2, Table S1 in File S2).

An average Ph+ ALL disease network model was computed following the same principles: From Mullighan et al., we extracted the 11 potential deletions to compute initial probabilities proportional to deletion frequencies (*IKZF1*, 84%; *CDKN2A*, 53%; *PAX5*, 51%; *C20orf94*, 23%; *RB1*, 19%; *MEF2C*, 14%; *EBF1*, 14%; *BTG1*, 14%; *DLEU*, 9%; *FHIT*, 9%; *ETV6*, 7%), with non-deleted genes having 0 probability [17]. BCR-ABL was added to this list with a probability corresponding to 100% frequency. Considering future utilization of genomic information for personalized therapy, for cell lines where detailed genomic information on the copy numbers of above disease genes was available in the recently released Cancer Cell Line Encyclopedia (CCLE, www.broadinstitute.org/ccle), we used this information to generate cell line-specific Ph+ ALL disease network models instead of the average patient model (Table S9 in File S1). For this purpose, we considered genes to be deleted, if the copy number was below -0.5, and amplified, if the copy number was above +0.5. All the deleted genes were given the same weight in the initial node probabilities.

To measure the correlation between a drug and a disease network, we introduced a correlation score (Figure 4B). Let be the probability of protein *i* in the disease network model, i.e. after diffusion of the initial node probabilities, and the equivalent in the drug treatment network model. We define

where *c*_*i*_ is a coefficient equal to 1 for non-deleted genes and 1-percentage/100 for the 11 deleted genes. The set *S* represents the proteins (network nodes) to consider in the summation. We determine *S* to contain the *n* largest products . Results in Table 2 were obtained with *n*=500, i.e. approximately 5% of the nodes. Other values of *n* gave very similar relative correlation scores.

**Table 2:** Network correlation scores P-values.

**Cell line**	**Average patient**	**BV-173**	**Z-119**	**SUP-B15**
**BCR-ABL**	-	p210	p190	p190
**Profile**	Max	BV-173	Z-119	Max
**Dis.Model**	Average	CCLE	Average	CCLE
**Nilotinib**	3.2E-1	2.7E-1	5.0E-1	4.4E-1
**Dasatinib**	**2.9E-3**	**5.3E-4**	4.9E-1	**2.1E-2**
**Bosutinib**	**2.9E-2**	**1.3E-2**	3.2E-1	7.7E-2
**Bafetinib**	**1.8E-2**	**5.0E-3**	6.1E-1	7.0E-2

Scores for correlation of drug-protein and disease-specific protein-protein interaction networks are dimension-less numbers. To bring all the scores of the 4 different drugs to a common scale we report their significance compared to a list of nonrelated diseases, the higher the impact of a drug on the disease network, the lower the reported P-value. Significant results (P-values ≤ 5%) are in bold and best significant result is underlined. BCR-ABL: Indicates BCR-ABL mutant. Profile: Target profile applied for correlation analysis as determined by chemical proteomics (Max stands for the maximum of the BV-173 and Z-119 profiles). Dis.Model: Disease-model applied for correlation analysis (Average stands for an average Ph+ ALL patient disease model with relative probabilities of disease gene deletions as reported by Mullighan et al.); CCLE stands for Cell line-specific Ph+ ALL disease-model taking into account the disease gene copy number as extracted from the Cancer Cell Line Encyclopedia (CCLE). The average patient model is used when no gene copy number information was deposited in CCLE.

Finally, since every drug targeted a different number of proteins with different abundance scores, it was necessary to operate normalization. This was achieved by scoring the treatment network models against a list of diseases[42] different from Ph+ ALL to obtain a null distribution for each kinase inhibitor. P-values corresponding to the actual Ph+ ALL cell types provided the normalized correlation scores (see File S2). 

 The correlation of the drug and disease networks produced scores for each TKI that allowed us to postulate which one of them would be the most efficient in the Ph+ ALL disease setting. Of all four drugs, dasatinib displayed the highest probability to affect the average Ph+ ALL disease network (dasatinib > bafetinib > bosutinib > nilotinib), as well as in the individual cell lines (Table 2). 

### Dasatinib shows the strongest antiproliferative effects on Ph+ ALL cell lines

**Figure 5:**
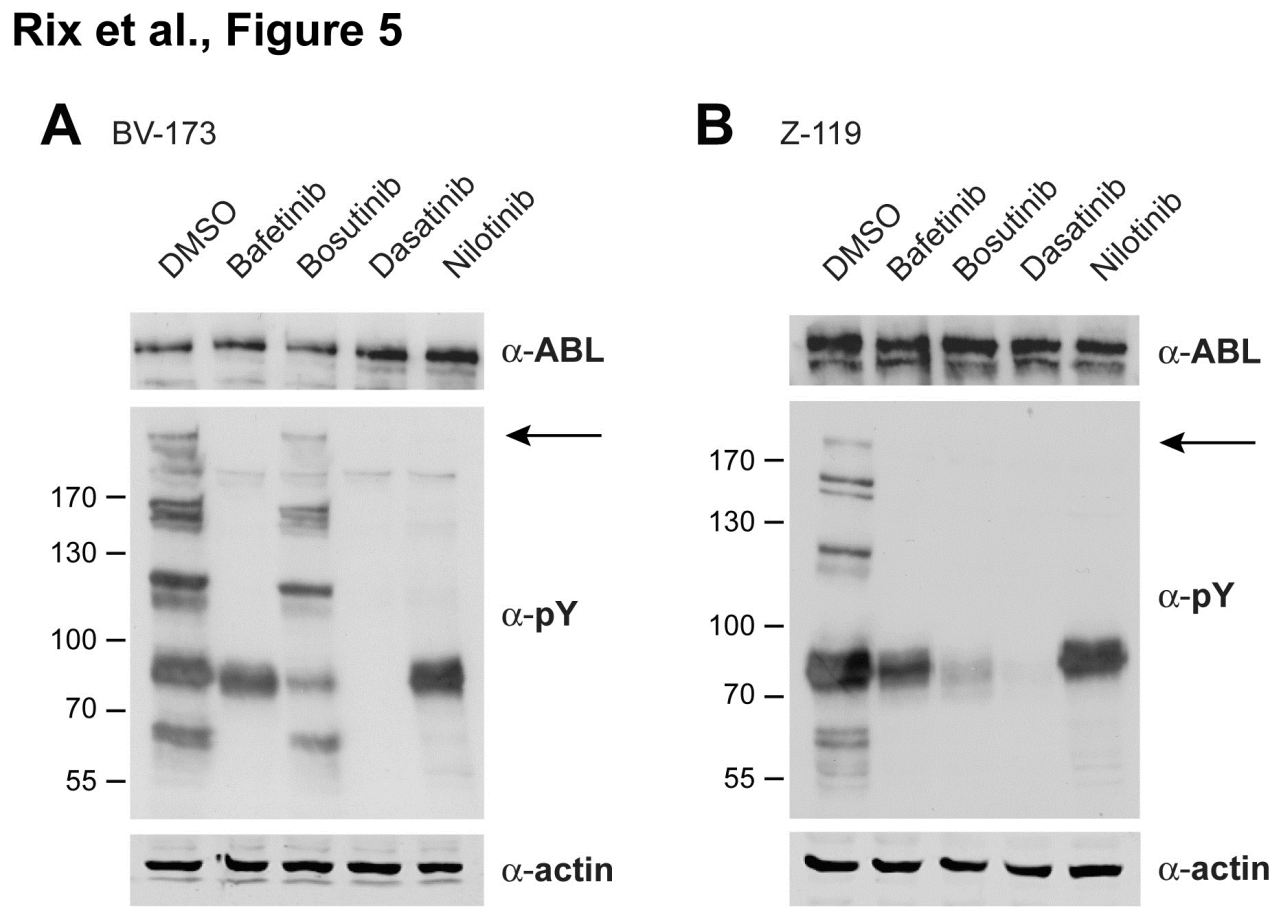
Differential drug effects on cellular tyrosine phosphorylation. Cells were treated for 30 min with bafetinib (800 nM), bosutinib (400 nM), dasatinib (100 nM) and nilotinib (4 µM), which are concentrations equivalent to reported maximum patient plasma concentrations, and DMSO control. Effects of individual drugs were determined by immunoblot analysis for BCR-ABL (α-ABL) and total phosphotyrosine (α-pY). Actin served as loading control. **A**. Dasatinib had the strongest impact on cellular tyrosine phosphorylation in BV-173 cells while the effects of bafetinib, nilotinib and particularly bosutinib were less pronounced. **B**. Dasatinib completely abolished cellular tyrosine phosphorylation in Z-119 cells. BCR-ABL levels were not appreciably affected, but it’s phosphorylation (marked by arrow) was inhibited by the drugs in either cell line.

Next, we asked whether our target and disease network-based prediction would correlate with cellular drug effects in Ph+ ALL cell lines. In a first step, we determined the drug-dependent phosphotyrosine signatures of BV-173 and Z-119 cells by immunoblot analysis when applying each drug at the respective maximal plasma concentration observed in patients. In correspondence to our prediction scores, dasatinib showed the strongest impact on tyrosine phosphorylation (Figure 5). Although all four drugs equally effectively abrogated phosphorylation of BCR-ABL in both cell lines (with the exception of bosutinib in BV-173 cells), several other signals that were lost upon dasatinib treatment were not affected to the same extent by bafetinib, bosutinib and nilotinib. This was particularly the case for the phosphotyrosine signature of bosutinib in BV-173 cells (Figure 5A), which suggests either a weak overall potency or that much of the net effect of bosutinib in these cells can be attributed to inhibition of serine/threonine kinases, which is supported by previous phosphoproteomics studies in CML (Bantscheff, Nat.Biotech., 2007; Winter, NCB, 2012). Interestingly, also dasatinib has a strong impact on the serine/threonine phosphorylation landscape (Bantscheff, Nat.Biotech., 2007; Pan, MCP, 2009), although this is different from bosutinib as these two drugs target very different serine/threonine kinases. 

To investigate the effects of TKI treatment on cell fate, we furthermore performed proliferation assays using [^3^H]thymidine incorporation and determined the IC_50_ values for each drug in a small panel of Ph+ ALL cell lines. Dasatinib was the most potent inhibitor of proliferation of BV-173 and Z-119 cells displaying subnanomolar IC_50_ values (Table 3, Figures S4-S6 in File S1). The remaining TKI were less effective than dasatinib in their antiproliferative effects. However, with the exception of bosutinib all second-generation TKI tested were more potent than imatinib. These tendencies and drug rankings also translated to another Ph+ ALL cell line, SUP-B15, which expresses the BCR-ABL^p190^ isoform. Superior dasatinib potency was further confirmed in a cell apoptosis assay in triplicates by means of Annexin V/PI staining for BV-173 and Z-119 cells (Fig. S7AB in File S1).

**Table 3:** Effects of various targeted drugs on Ph+ ALL cell lines*.

	**BV-173**	**Z-119**	**SUP-B15**
**Imatinib**	23.5	65.2	338.4
**Nilotinib**	1.0	5.3	3.5
**Dasatinib**	0.1	0.2	0.4
**Bosutinib**	22.0	86.9	706.5
**Bafetinib**	0.6	0.6	0.9

* Results were obtained through measurement of ^3^H-thymidine uptake of the cell lines; IC_50_ values are given in nM (detailed cell proliferation curves in Figures S4-S6 in File S1).

## Discussion

In this study we have addressed the question, which of the four second-generation TKI in clinial trials, nilotinib, dasatinib, bosutinib or bafetinib, has the most advantageous target profile in the context of Ph+ ALL. Ph+ ALL features a complex genetic background on top of the expression of the oncogenic tyrosine kinase BCR-ABL [17]. This genetic complexity supposedly reduces the long-term effectiveness of BCR-ABL-directed therapy with imatinib [13]. We therefore compared the four TKI in light of their impact on a Ph+ ALL PPI network, taking into account the additional gene copy number alterations that distinguish Ph+ ALL from CML rather than focussing on classical BCR-ABL signaling alone [17].

We performed a network analysis based on a diffusion procedure. A similar approach has been employed recently to predict drug side effects with regard to heart arrhythmias [44]. To this end, the authors were successful by focussing on the cognate drug targets. However, the well-documented pleiotropic nature of kinase inhibitors, which were the focus of interest here, made it necessary for our study to first determine the proteome-wide Ph+ ALL target profiles of each drug by chemical proteomics. 

Some of the described genetic lesions in Ph+ ALL have been previously demonstrated to be also of functional relevance. For instance, IKAROS, encoded by *IKZF1*, has been shown to redirect BCR-ABL signaling from SFK activation to SLP65, which is downstream of the pre-B cell receptor tumor suppressor [45]. In this way, loss of IKAROS promotes oncogenic signaling of BCR-ABL in part by phosphorylation and activation of the SRC family kinases LYN, HCK and FGR. These kinases have been previously demonstrated to be required for induction of Ph+ ALL while being dispensable for CML [46]. In a similar way, BTK has been shown to be constitutively activated by BCR-ABL in Ph+ ALL cells thereby bypassing the pre-B cell receptor and providing a continuous survival signal [47].

LYN and BTK have been identified with all four TKI in the present chemical proteomics screen. However, there were pronounced differences in the purification yields as indicated by the abundance score *A*. In fact, the scores for dasatinib and bosutinib for these kinases were among the highest that were observed for all drug-protein interactions and yet higher than the ones for BCR-ABL. Consistenly, dasatinib and bosutinib are known to be highly potent inhibitors of all SFK and BTK with *in vitro* kinase assays showing single-digit nanomolar IC_50_’s [6,7,24,48]. Bafetinib, although also a LYN inhibitor (IC_50_ = 51 nM), is less potent than these while nilotinib displays only micromolar inhibition of LYN [26,34]. Neither bafetinib nor nilotinib have been implicated as significant BTK inhibitors. However, as identification of proteins by chemical proteomics depends not only on affinity, but also on abundance, BTK purification by these drugs might be due to the combination of high BTK expression levels and low drug affinity. Post-translational modifications, such as phosphorylation or ubiquitination, mutations and differential splicing might also have an impact on protein conformation and drug affinity. It is notable that BV-173 cells have been described previously to feature BTK phosphorylation as well as truncated BTK isoforms with altered biochemical properties [47,49]. The ‘*A*’ scores, which were developed to represent target abundance in the eluate, are directly incorporated in the subsequent random walk analysis. In addition, LYN in particular is strongly connected to the reported gene deletions as it is separated from four deleted nodes (CDKN2A, RB1, FHIT, ETV6) by only one other protein (Table S10 in File S1), i.e. it is interacting with proteins that are directly affected by the loss of one of the disease genes. Although the overall network correlation scores cannot be fully explained by effects on single nodes, but are the sum of the global drug effects, the observed LYN and BTK enrichment patterns may explain to some extent the score differences between dasatinib on the one hand and nilotinib and bafetinib on the other hand. Bosutinib, however, has only slightly lower ‘*A*’ scores for LYN and BTK than dasatinib. The difference between dasatinib and bosutinib might therefore be attributed to the contribution that is made by serine/threonine kinases to the bosutinib network score, which would be in line with the relatively weak effect of bosutinib on tyrosine phosphorylation in BV-173 cells. Additionally, this could be at least in part due to other dasatinib targets, such as TEC or ILK, which are not or only marginally seen with bosutinib. For instance, the entire ILK/LIMS1/PARVIN/RSU1 complex is engaged specifically by dasatinib, produces relatively high ‘*A*’ scores and is well connected to disease genes through ILK and LIMS1 (e.g. *FHIT, RB1, IKZF1*) (Table S10 in File S1). Interestingly, we have identified BCR-ABL, LYN, BTK, TEC and ILK as dasatinib interactors also in the pool of Ph+ ALL patient PBMCs and subsequently confirmed their expression in each individual patient from the pool by qPCR (Table S11 in File S1), which highlights their potential relevance in the context of mediating network-wide drug effects in Ph+ ALL.

One would intuitively expect that the drug with the widest target spectrum, in this case bosutinib, would produce the strongest network effects. However, the network correlation analysis suggested dasatinib to have the most favorable drug-protein interaction profile in Ph+ ALL. This was consistent with the demonstrated important role of, for instance, BCR-ABL, LYN and BTK in Ph+ ALL and the fact that dasatinib displayed the strongest impact on these kinases.

Overall, nilotinib, bosutinib and bafetinib were predicted to be inferior to dasatinib. This prediction was well reflected by the IC_50_’s in cellular proliferation assays and was further improved when based on more detailed genomic information as accessible in the CCLE database. This suggests that incorporation of patient gene signatures, as they will become available in the future, has the potential to produce valuable predictions for individual Ph+ ALL patients. Notably, these observations also correlated well with published, in part preliminary reports from clinical trials with the individual drugs applied as monotherapies or in combination with chemotherapy [12,13,50-53].

Being critical about the correlation analysis also revealed two points worth discussing for future applications. First, although nilotinib is a potent kinase inhibitor, as e.g. observed in Table 3 and Figure S7C in File S1, and its couplable derivate pc-nilotinib showed well preserved potency in a c-ABL kinase assay, when linked to beads this compound might have modified binding abilities as indicated by low BCR-ABL spectral counts in Table 1 and poor scores in Tables 2 and S1 in File S2. In addition, post-translational modifications on BCR-ABL and its interaction partners in Ph+ ALL cells, as well as the different BCR-ABL isoforms themselves, may influence drug binding properties compared to c-ABL. This highlights the importance of performing experiments in the correct cell type, ideally from patient biopsies, and having detailed information about genetic alterations is likely to be essential as well. As a matter of fact, our correlation analysis performed better with BV-173 than with Z-119 cells (Tables 2 and 3). Z-119 cells respond to kinase inhibitors very differently compared to BV-173 cells, as can be for instance appreciated from Figure S7C in File S1, and their genetic alterations were not mapped in detail (we used an average patient model) whereas for BV-173 the CCLE database provided detailed genetic data. To use the correct cell type has the potential to reveal changes at the compound-target interaction level and the genetic alterations can inform on possible downstream signalling changes when mapped onto the appropriate network.

In summary, we here present a systems biology-derived network model for assisting implementation of personalized therapy in Ph+ ALL with second-generation BCR-ABL inhibitors. This model is based on the comprehensive, proteome-wide survey of the drug-target profiles of nilotinib, dasatinib, bosutinib and bafetinib in the context of the complex Ph+ ALL-specific protein-protein interaction network. Correlation analysis elected dasatinib as the most effective network drug for Ph+ ALL. This prediction was validated by cellular proliferation assays. First clinical reports show that dasatinib indeed has favorable efficacy. This type of study was designed to serve the community to evaluate these drugs based on their cellular target profile. In future, as it will not always be feasible to test their effects directly on patient cells, it should be useful to annotate these networks with mutation and expression data to derive a patient-specific simulation.

## Supporting Information

File S1 Figure S1. ABL in vitro kinase assay. Figure S2. Ph+ ALL-specific kinome-wide drug-target maps of nilotinib, dasatinib, bosutinib and bafetinib displaying new and known targets. Figure S3. Hybrid drug-protein/protein-protein interaction networks of specific dasatinib binding proteins in the patient pool sample. Figure S4. Measurement of 3H-thymidine uptake in BV-173 cells. Figure S5. Measurement of 3H-thymidine uptake in Z-119 cells. Figure S6. Measurement of 3H-thymidine uptake in SUP-B15 cells. Figure S7. Measurement of cell apoptosis in BV-173 and Z-119 cells. Table S1. Molecular Characteristics of patient samples. Table S2. Mass spectrometry data for drug affinity pulldowns in BV-173 cells. Table S3. Mass spectrometry data for drug affinity pulldowns in Z-119 cells. Table S4. Mass spectrometry data for dasatinib affinity pulldowns in primary cells from Ph+ ALL patient pool. Table S5. IC50 values (in nM) of selected new drug-kinase interactions determined by in vitro kinase assays. Table S6. Mass spectrometry data for competitive drug affinity pulldowns in BV-173 cells. Table S7. Mass spectrometry data for competitive drug affinity pulldowns in Z-119 cells. Table S8. Eluate abundance scores A for each specific drug-binding protein identified in BV-173 cells. Table S9. Eluate abundance scores A for each specific drug-binding protein identified in Z-119 cells. Table S10. Gene copy numbers of the 11 Ph+ ALL disease genes as reported in the Cancer Cell Line Encyclopedia (CCLE). Table S11. Distances between each specific drug-binding protein and the deleted disease nodes within the Ph+ ALL disease-modified PPI network. Table S12. Expression analysis of BTK, LYN, ILK and TEC by qPCR in individual Ph+ ALL patient samples and cell lines.
(PDF)

File S2 Figure S1. The example of how a single seed diffuses information towards its neighbors. Figure S2. Three variants of a random walk. Figure S3. Computation of the disease P-values. Table S1. Disease network model P-values sorted in ascending order for dasatinib.
(PDF)
