# Supplementary material for: A Target-Disease Network Model of Second-Generation BCR-ABL Inhibitor Action in Ph+ ALL
Source: PLoS One. 2013 Oct 10;8(10):e77155. doi: 10.1371/journal.pone.0077155 (PMC3795025; doi:10.1371/journal.pone.0077155)
Supplement: File S1 — Figure S1. ABL in vitro kinase assay. Figure S2. Ph+ ALL-specific kinome-wide drug-target maps of nilotinib, dasatinib, bosutinib and bafetinib displaying new and known targets. Figure S3. Hybrid drug-protein/protein-protein interaction networks of specific dasatinib binding proteins in the patient pool sample. Figure S4. Measurement of 3H-thymidine uptake in BV-173 cells. Figure S5. Measurement of 3H-thymidine uptake in Z-119 cells. Figure S6. Measurement of 3H-thymidine uptake in SUP-B15 cells. Figure S7. Measurement of cell apoptosis in BV-173 and Z-119 cells. Table S1. Molecular Characteristics of patient samples. Table S2. Mass spectrometry data for drug affinity pulldowns in BV-173 cells. Table S3. Mass spectrometry data for drug affinity pulldowns in Z-119 cells. Table S4. Mass spectrometry data for dasatinib affinity pulldowns in primary cells from Ph+ ALL patient pool. Table S5. IC50 values (in nM) of selected new drug-kinase interactions determined by in vitro kinase assays. Table S6. Mass spectrometry data for competitive drug affinity pulldowns in BV-173 cells. Table S7. Mass spectrometry data for competitive drug affinity pulldowns in Z-119 cells. Table S8. Eluate abundance scores A for each specific drug-binding protein identified in BV-173 cells. Table S9. Eluate abundance scores A for each specific drug-binding protein identified in Z-119 cells. Table S10. Gene copy numbers of the 11 Ph+ ALL disease genes as reported in the Cancer Cell Line Encyclopedia (CCLE). Table S11. Distances between each specific drug-binding protein and the deleted disease nodes within the Ph+ ALL disease-modified PPI network. Table S12. Expression analysis of BTK, LYN, ILK and TEC by qPCR in individual Ph+ ALL patient samples and cell lines. (PDF) [file pone.0077155.s001.pdf]

Supplementary Information

A target-disease network model of second-generation BCR-ABL inhibitor action in Ph+ ALL

Uwe Rix[1*¶], Jacques Colinge[1*], Katharina Blatt[2], Manuela Gridling[1], Lily L. Remsing Rix,[1¶] Katja Parapatics[1], Sabine Cerny-Reiterer[2,3], Thomas R. Burkard[1#], Ulrich Jäger[2], Junia V. Melo[4], Keiryn L. Bennett[1], Peter Valent[2,3] and Giulio Superti-Furga[1]

[1]CeMM – Research Center for Molecular Medicine of the Austrian Academy of Sciences, Vienna, Austria; [2]Department of Internal Medicine I, Division of Hematology and Hemostaseology, Medical University of Vienna, Vienna, Austria; [3]Ludwig Boltzmann Cluster Oncology, Vienna, Austria; [4]Department of Haematology, SA Pathology Centre for Cancer Biology, Adelaide, Australia

*These two authors contributed equally

¶Current address: H. Lee Moffitt Cancer Center & Research Institute, Tampa, FL, USA

#Current address: IMBA - Institute for Molecular Biotechnology of the Austrian Academy of Sciences, Vienna, Austria

Figure S1. ABL *in vitro* kinase assay. A. Chemical structures of nilotinib, pc-nilotinib and c-nilotinib and synthetic modification of pc-nilotinib generating c-nilotinib. Boc-Gly-OH: *N*-(*tert*-Butoxycarbonyl)glycine, DCC: *N,N'*-Dicyclohexylcarbodiimide, DMAP: 4-(Dimethylamino)pyridine, TFA: Trifluoroacetic acid. **B.** *In vitro* kinase inhibition assay of nilotinib and pre-c-nilotinib (pc-nilotinib), the non-esterified precursor of c-nilotinib, using recombinant full-length c-ABL determines similarly potent inhibition of ABL by nilotinib and pc-nilotinib.

Figure S2. Ph+ ALL-specific kinome-wide drug-target maps of nilotinib, dasatinib, bosutinib and bafetinib displaying new and known targets. Ph+ ALL kinase targets of the TKI nilotinib (A), dasatinib (B), bosutinib (C) and bafetinib (D) that have been identified in this study were mapped to the phylogenetic tree of human protein kinases. Known kinase targets of the respective drugs are shown in blue, new targets in grey. Color intensities indicate the degree of amino acid (aa) sequence

coverage observed for each protein. A black dotted rim indicates independent validation by various *in vitro* assays that have been reported previously, a red dotted rim indicates validation of drug-protein interactions performed in the course of this study (see Table S4 in this file), selecting the most prominent kinase targets (based on the aa sequence coverage) without previous independent validation. The Human Kinome Map was adapted with permission from Cell Signaling Technology (www.cellsignal.com).

Figure S3. Hybrid drug-protein/protein-protein interaction networks of specific dasatinib binding proteins in the patient pool sample. The primary cellular target profile of dasatinib (red) was overlaid with PPI data from public databases. Protein kinases were considered to be direct drug binders (solid lines) and displayed in red. All other non-kinase proteins were assumed to be indirect binders (dashed lines) and are displayed in grey.

Figure S4. Measurement of ^{3}H-thymidine uptake in BV-173 cells.

Figure S5. Measurement of ^{3}H-thymidine uptake in Z-119 cells.

Figure S6 Measurement of ^{3}H-thymidine uptake in SUP-B15 cells.

Figure S7. Measurement of cell apoptosis in BV-173 and Z-119 cells.

Table S1. Molecular Characteristics of patient samples. WBC: White blood cell count; Hb: Hemoglobin; Plt: Platelet count.

Table S2. Mass spectrometry data for drug affinity pulldowns in BV-173 cells. AC: Swissprot accession code. Entrez-ID: Entez accession code. Entrez_GN: alternative gene names. PC: number of unique peptides (peptide count). SC: number of mass spectra to sequence matches (spectral count). SeqCov: percentage (1.00=100%) of amino acid sequence for individual protein that is cumulatively covered by all assigned unique peptides (sequence coverage). Numbers following drug names are indicating the individual replicates (1,2: technical replicates of experimental replicate 1; 3,4: technical replicates of experimental replicate 2). 'grp' indicates the overall peptide counts, spectral

counts or sequence coverage across all replicates. Each row represents one group of proteins, the representative of which is listed in detail.

Table S3. Mass spectrometry data for drug affinity pulldowns in Z-119 cells. For detailed description *see* Table S2 in this file.

Table S4. Mass spectrometry data for dasatinib affinity pulldowns in primary cells from Ph+ ALL patient pool. For detailed description *see* Table S2 in this file.

Table S5. IC_{50} values (in nM) of selected new drug-kinase interactions determined by *in vitro* kinase assays.

Table S6. **Mass spectrometry data for competitive drug affinity pulldowns in BV-173 cells.** Competition was performed with concomitant incubation of drug beads with the respective original drug at a concentration of 20 µM. For detailed description *see* Table S3 in this file.

Table S7. **Mass spectrometry data for competitive drug affinity pulldowns in Z-119 cells.** Competition was performed with concomitant incubation of drug beads with the respective original drug at a concentration of 20 µM. For detailed description *see* Table S3 in this file.

Table S8. Eluate abundance scores A for each specific drug-binding protein identified in BV-173 cells. AC: Swissprot accsession code. ID: Uniprot identifier. Individual scores were calculated as the product of the mean spectral count and the 'grp' sequence coverage extracted from Table S3 in this file.

Table S9. Eluate abundance scores A for each specific drug-binding protein identified in Z-119 cells. AC: Swissprot accsession code. ID: Uniprot identifier. Individual scores were calculated as the product of the mean spectral count and the 'grp' sequence coverage extracted from Table S4 in this file.

Table S10. Gene copy numbers of the 11 Ph+ ALL disease genes as reported in the Cancer Cell Line Encyclopedia (CCLE).

Table S11. Distances between each specific drug-binding protein and the deleted disease nodes within the Ph+ ALL disease-modified PPI network. AC: Swissprot accsession code. ID: Uniprot identifier. 'NA' indicates proteins that were not represented in any PPI database and were therefore not mapped onto the network.

Table S12. Expression analysis of BTK, LYN, ILK and TEC by qPCR in individual Ph+ ALL patient samples and cell lines.

Structural characterization of coupleable drug analogues

pc-Nilotinib

M = 587.59 g/mol ($C_{31}H_{28}F_3N_7O_2$)

LC/MS Purity: 99%

MS (pos.-ESI) m/z 588.0 $[M+H]^+$(66), 294.6 $[M+2H]^{2+}$ (100).

^{1}H-NMR (400 MHz, CD_3OD): δ□ 9.09 (bs, 1H), 8.55 (s, 1H), 8.50 (s, 1H), 8.49 (d, 1H), 8.46 (s, 1H), 8.25 (s, 1H), 8.13 (s, 1H), 8.11 (s, 1H), 7.71 (d, 1H), 7.61 (s, 1H), 7.43 (d, 1H), 7.41 (d, 1H), 7.37 (s, 1H), 3.52 (t, 2H), 2.81 (t, 2H), 2.43 (s, 3H), 2.27 (s, 3H), 1.85 (m, 2H).

c-Dasatinib

M = 487.02 g/mol ($C_{22}H_{27}ClN_8OS$)

LC/MS Purity: 96%

MS (pos.-ESI) *m/z* 487.3/489.2 $[M+H]^+$(100/35).

^{1}H-NMR (400 MHz, CD_3OD): δ□ 8.19 (s, 1H), 7.35 (dd, 1H), 7.26-7.22 (m, 1H), 7.24-7.20 (m, 1H), 6.34 (s, 1H), 3.94 (b, 4H), 3.35-3.29 (m, 2H), 3.21-3.14 (m, 6H), 2.54 (s, 3H), 2.31 (s, 3H).

c-Bosutinib

M = 696.93 g/mol ($C_{29}H_{36}Cl_2N_6O_3$*3HCl), free base 587.54 g/mol ($C_{29}H_{36}Cl_2N_6O_3$)

LC/MS Purity: 96%

MS (pos.-ESI) *m/z* 587.1/589.0 $[M+H]^+$(56/41).

^{1}H-NMR (300 MHz, DMSO-d_6): δ□ 12 (bs, 1H), 11.15 (bs, 1H), 8.97 (s, 1H), 8. 31 (s, 1H), 8.03 (bs, 2H), 7.84 (s, 1H), 7.59 (s, 1H), 7.51 (s,1H), 6.1-5.1(b,2H), 4.34 (t,2H), 4.04 (s, 3H), 3.88 (s,3H), 3.66 (bs, 6H), 3.56 (bs,4H), 3.35 (m,2H), 2.81 (m,2H), 2.36 (bs,2H), 1.81 (bs,2H), 1.64 (bs,2H).

<u>pc-Bafetinib</u>

M = 633.71 g/mol ($C_{34}H_{38}F_3N_7O_2$)

LC/MS Purity: 96%

MS (pos.-ESI) *m/z* 634.3 $[M+H]^+$(37), 317.7 $[M+2H]^{2+}$ (100).

^{1}H-NMR (400 MHz, $CDCl_3$): δ□ 9.00 (d, 1H), 8.68 (bs, 1H), 8.53 (d, 1H), 8.50 (d, 1H), 8.40 (bs, 1H), 8.16 (s, 1H), 8.07 (s, 1H)*, 8.02 (d, 1H), 7.92 (d, 1H), 7.28-7.20 (m, 2H), 7.18 (d, 1H), 7.06 (s, 1H)*, 3.82 (dd, 1H), 3.71 (dd, 1H), 3.47 (m,2H), 2.81 (m, 1H), 2.79 (m, 2H), 2.73-2.60 (m, 3H), 2.48 (m, 1H), 2.29 (s, 3H), 2.17 (s, 6H), 2.05-1.90 (m, 1H), 1.88-1.75 (m, 3H).

Rix et al., Supplementary Figure 2

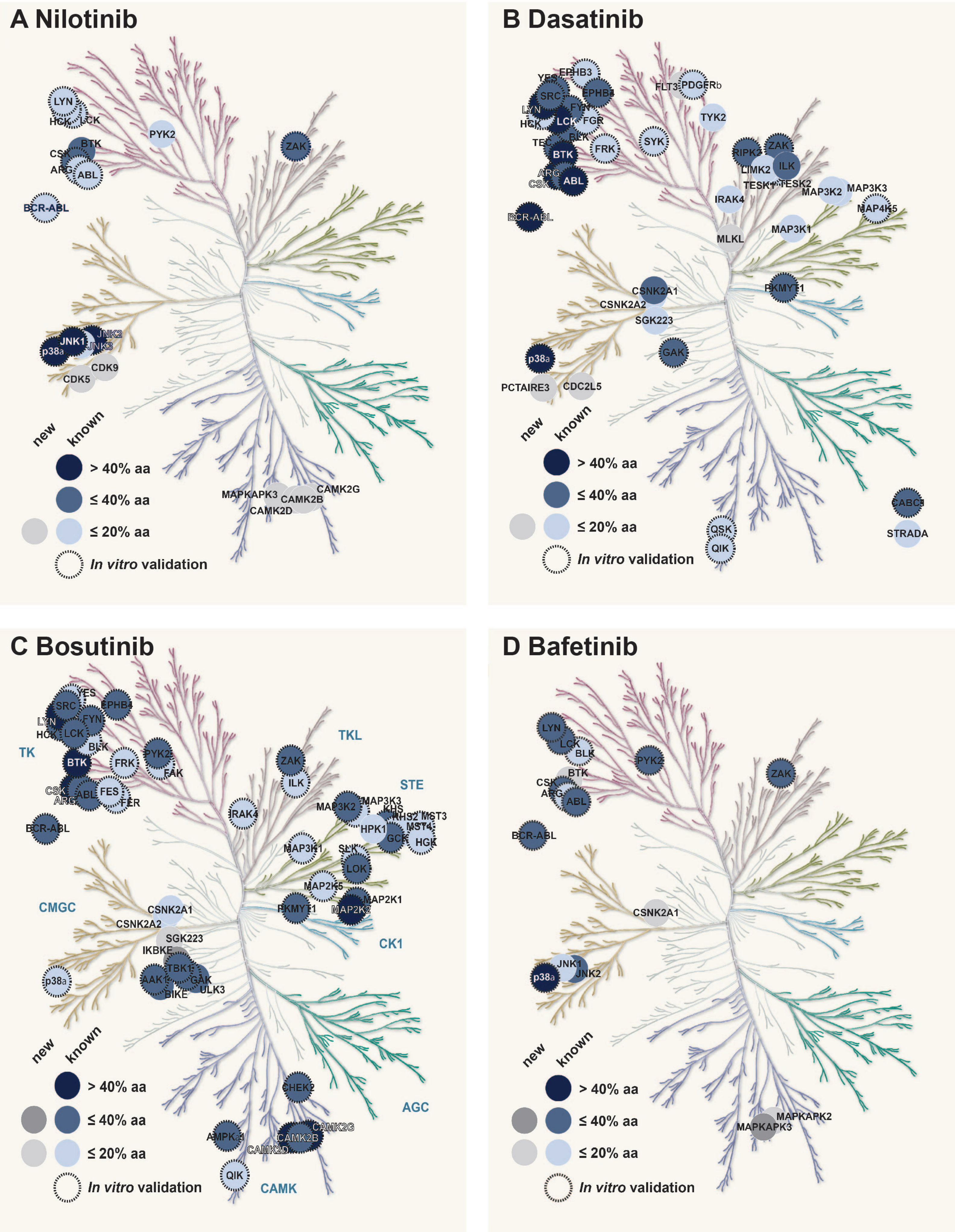

Rix et al., Supplementary Figure 3

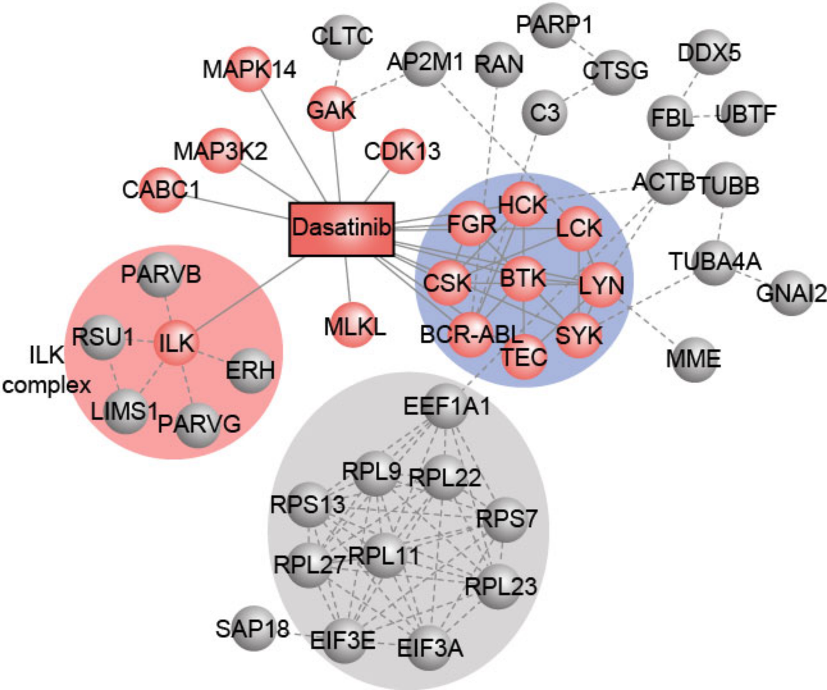

Rix et al., Figure S4

Effect of Imatinib on ^{3}H-Thymidine Incorporation by BV173 Cells

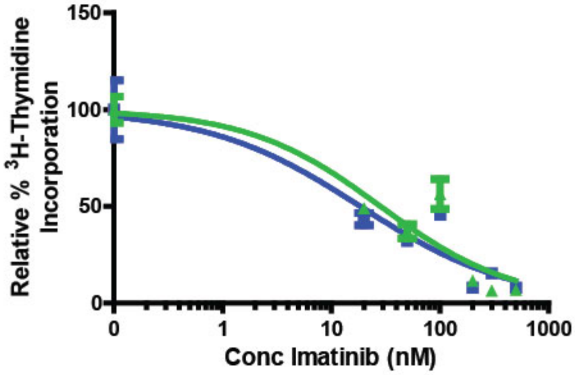

	Exp #1	Exp #2
EC50	18.50	28.41

Effect of Nilotinib on ^{3}H-Thymidine Incorporation by BV173 Cells

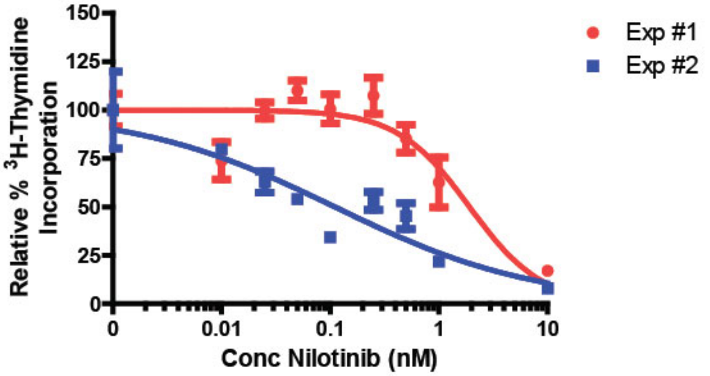

	Exp #1	Exp #2
EC50	1.921	0.1108

Effect of Dasatinib on ^{3}H-Thymidine Incorporation by BV173 Cells

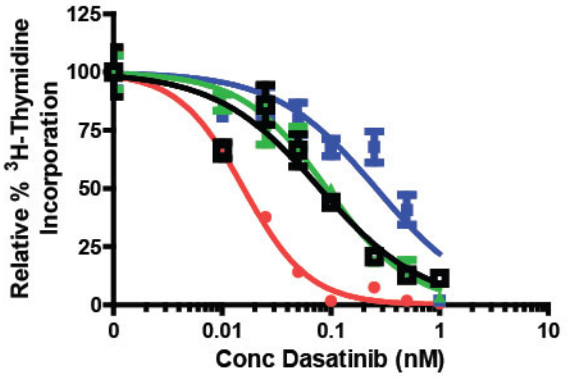

	Exp #1	Exp #2	Exp #3	Exp #4
EC50	0.01577	0.2615	0.09191	0.07953

Effect of Bosutinib on ^{3}H-Thymidine Incorporation by BV173 Cells

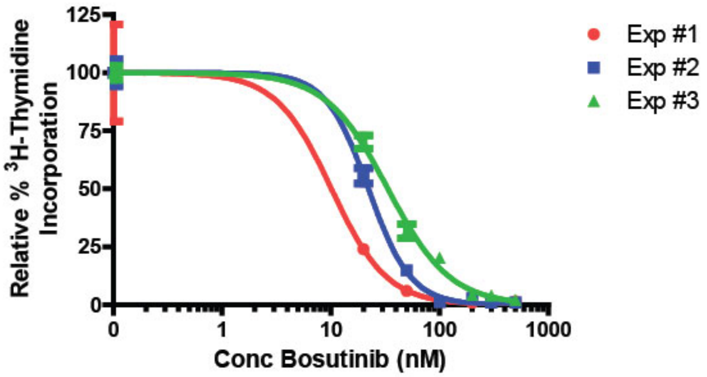

	Exp #1	Exp #2	Exp #3
EC50	10.14	22.20	33.55

Effect of Bafetinib on ^{3}H-Thymidine Incorporation by BV173 Cells

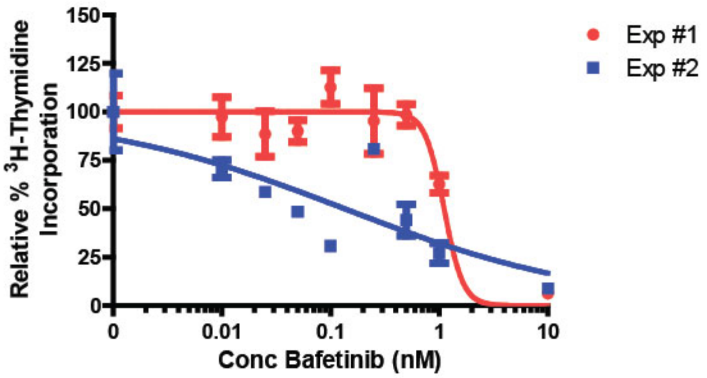

	Exp #1	Exp #2
EC50	1.111	0.1355

Rix et al., Figure S5

Effect of Imatinib on ^{3}H-Thymidine Incorporation by Z119 Cells

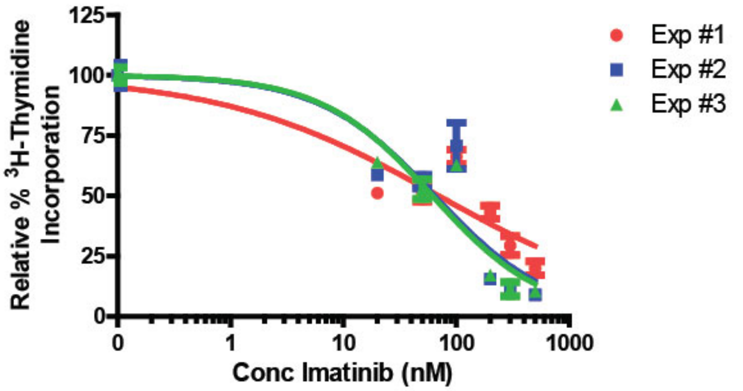

	Exp #1	Exp #2	Exp #3
EC50	69.98	64.23	61.44

Effect of Nilotinib on ^{3}H-Thymidine Incorporation by Z119 Cells

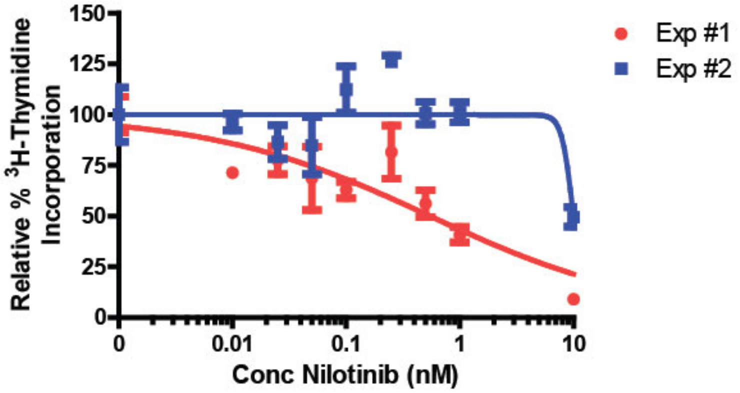

	Exp #1	Exp #2
EC50	0.5506	~ 9.982

Effect of Dasatinib on ^{3}H-Thymidine Incorporation by Z119 Cells

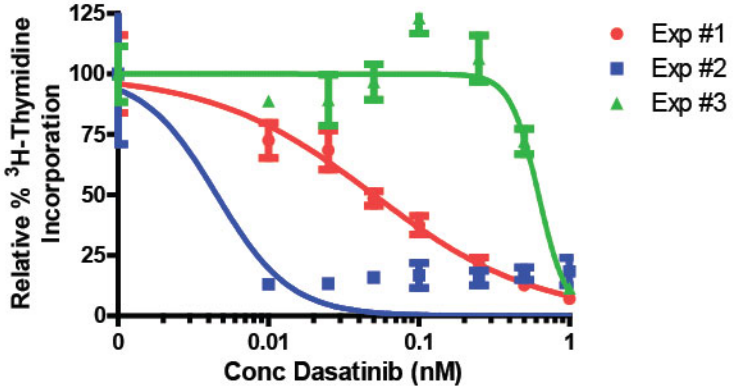

	Exp #1	Exp #2	Exp #3
EC50	0.05004	0.004533	0.6217

Effect of Bosutinib on ^{3}H-Thymidine Incorporation by Z119 Cells

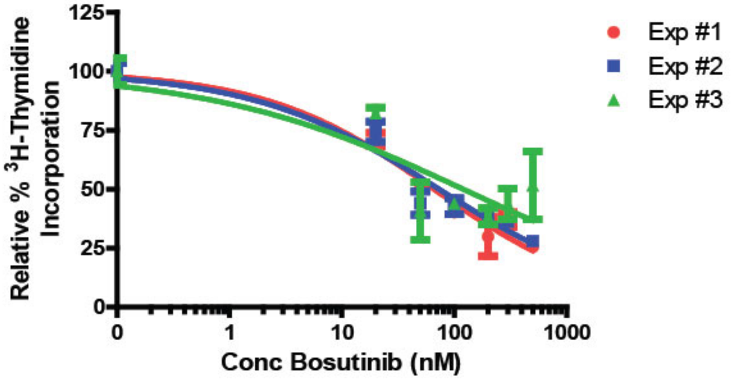

	Exp #1	Exp #2	Exp #3
EC50	66.46	73.47	120.8

Effect of Bafetinib on ^{3}H-Thymidine Incorporation by Z119 Cells

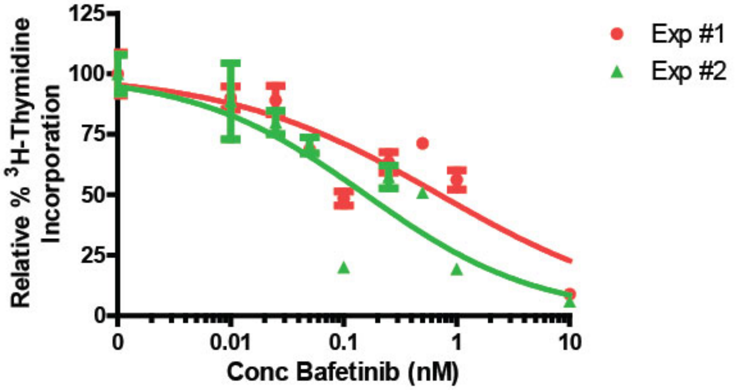

	Exp #1	Exp #2
EC50	0.9293	0.2781

Rix et al., Figure S6

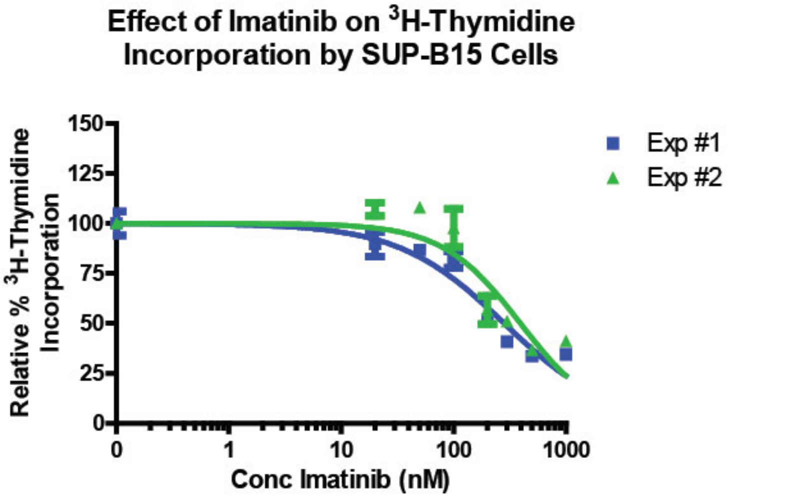

	Exp #1	Exp #2
EC50	282.5	394.2

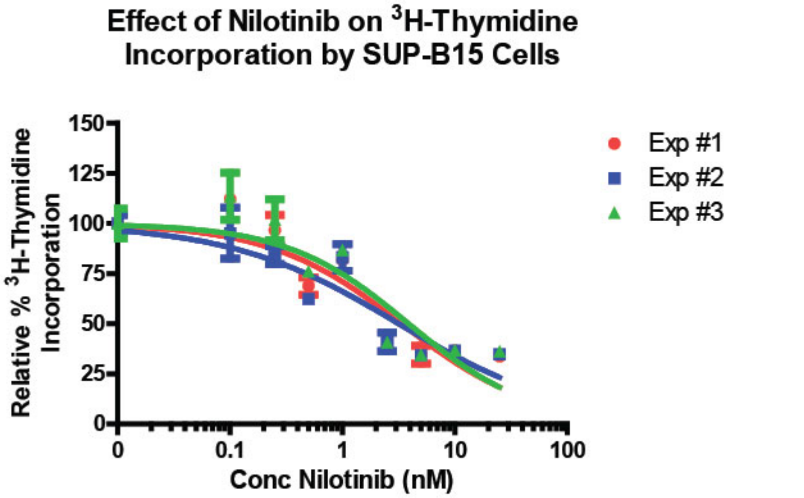

	Exp #1	Exp #2	Exp #3
EC50	3.367	3.193	3.901

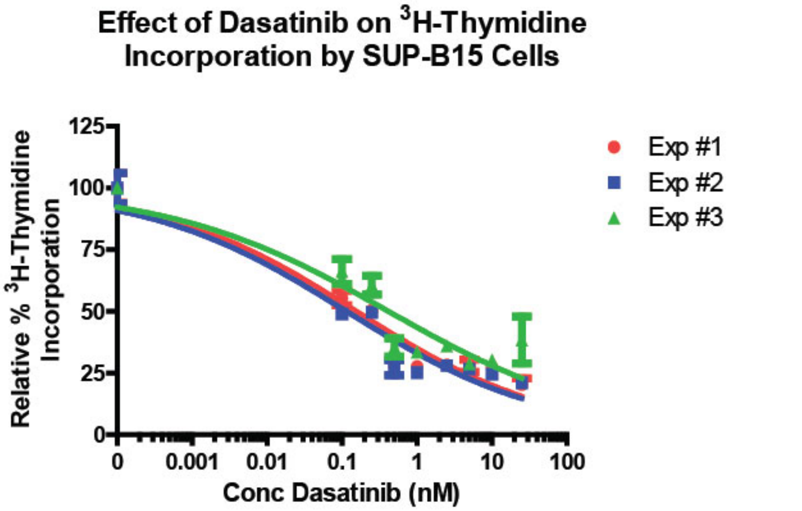

	Exp #1	Exp #2	Exp #3
EC50	0.2619	0.2158	0.5736

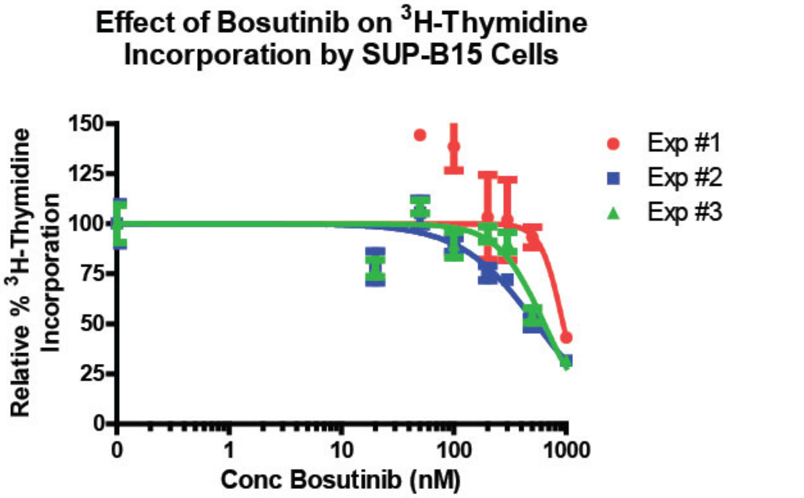

	Exp #1	Exp #2	Exp #3
EC50	939.1	541.5	638.9

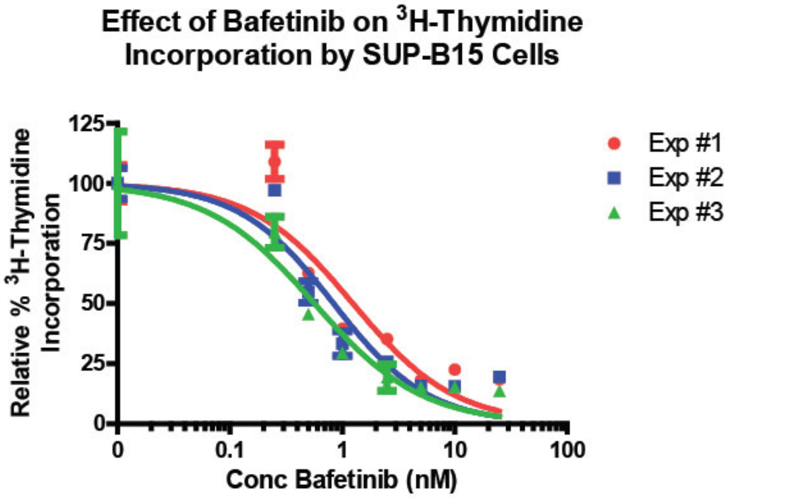

	Exp #1	Exp #2	Exp #3
EC50	1.246	0.8384	0.5664

Rix et al., Figure S7

A BV173

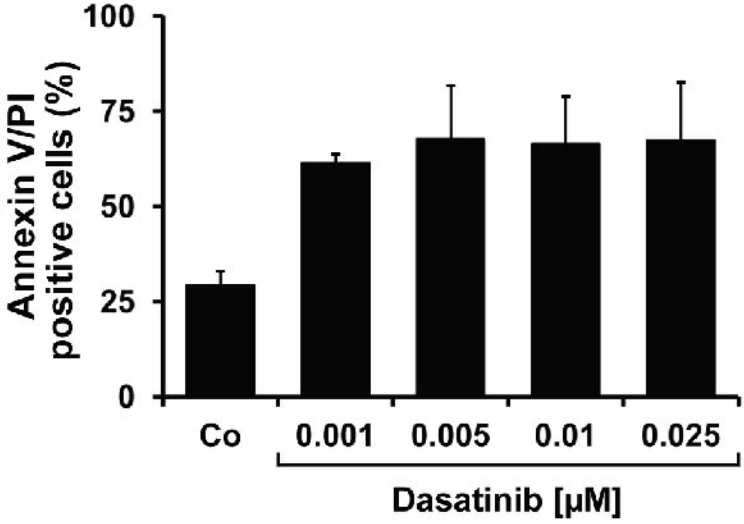

B Z119

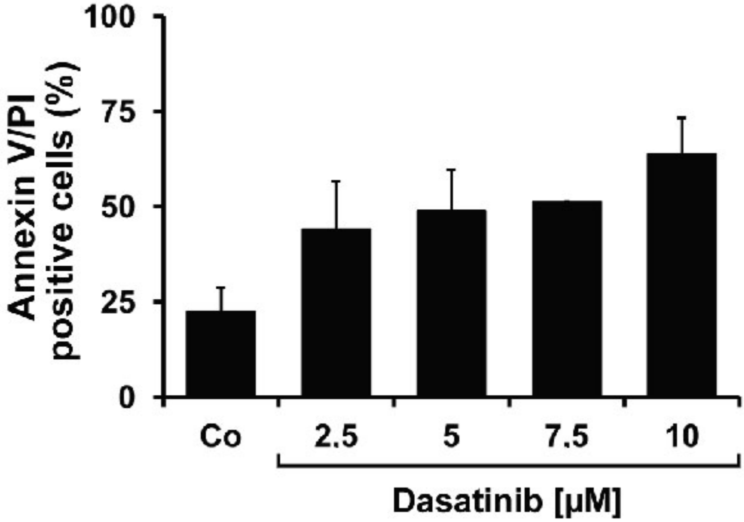

C

ED_{50}	BV173	Z119
**Imatinib**	0.1 µM	>10 µM
**Nilotinib**	0.005 µM	>10 µM
**Dasatinib**	<0.001 µM	7.5 µM
**Bosutinib**	0.1 µM	2.5 µM

Patient	Gender	Age (y)	Diagnosis	BCR-ABL	WBC (G/L)	Hb (g/dL)	Plt (G/L)	Cytogenetics
1	m	73	pre-B-ALL	y	140	10	19	46, XY, t(9;22)(q34;q11)
2	m	39	c-ALL	y	61.6	9.4	56	46, XY, t(9;22)(q34;q11)
3	f	35	c-ALL	y	24.72	13.1	177	complex
4	f	56	pre-B-ALL	y	2.3	7.4	86	46, XX, t(9;22)(q34;q11)
5	f	55	c-ALL	y	71.3	13.7	73	46, XX, t(9;22)(q34;q11)
6	f	37	pre-B-ALL	y	4.4	7.6	3	46, XX

AC	Entrez_ID	Gene_symbol	Descr	Entrez_GN	pc_Dasatinib_1	pc_Dasatinib_2	pc_Dasatinib_3	pc_Dasatinib_4	pc_Dasatinib_grp
Q04446	2632	GBE1	1,4-alpha-glucan-branching enzyme	GBE1,GBE	0	0	0	0	0
P61604	3336	HSPE1	10 kDa heat shock protein, mitochondrial	HSPE1,CPN10,GROES,HSP10	0	0	0	0	0
P31946-1	7529	YWHAB	14-3-3 protein beta/alpha	YWHAB,KCIP-1,GW128,HS1	7	5	5	5	7
P62258	7531	YWHAE	14-3-3 protein epsilon	YWHAE,MDS,MDCR,KCIP-1,14-3-3E,FLJ45465	8	6	7	7	8
Q04917	7533	YWHAH	14-3-3 protein eta	YWHAH,YWHA1	5	4	5	4	5
P61981	7532	YWHAG	14-3-3 protein gamma	YWHAG,14-3-3GAMMA	9	6	8	6	10
P31947-1	2810	SFN	14-3-3 protein sigma	SFN,YWHAS	3	3	3	3	3
P27348	10971	YWHAQ	14-3-3 protein theta	YWHAQ,1C5,HS1,14-3-3	8	4	7	6	9
P63104	7534	YWHAZ	14-3-3 protein zeta/delta	YWHAZ,KCIP-1,MGC111427,MGC126532,MGC138156,14-3-3-zeta	10	9	9	7	12
P62263	6208	RPS14	40S ribosomal protein S14	RPS14,EMTB	0	0	0	0	0
P62269	6222	RPS18	40S ribosomal protein S18	RPS18,KE3,HKE3,KE-3,D6S218E,MGC117351,MGC126835,MGC126837	0	0	0	0	0
P39019	6223	RPS19	40S ribosomal protein S19	RPS19,DBA	0	0	0	0	0
P62851	6230	RPS25	40S ribosomal protein S25	RPS25	0	0	2	2	2
P62854	6231	RPS26	40S ribosomal protein S26	RPS26,MGC104292	2	0	2	2	2
P23396	6188	RPS3	40S ribosomal protein S3	RPS3,FLJ26283,FLJ27450,MGC87870	0	0	0	0	0
P61247	6189	RPS3A	40S ribosomal protein S3a	RPS3A,FTE1,MFTL,MGC23240	0	0	0	0	0
P62701	6191	RPS4X	40S ribosomal protein S4, X isoform	RPS4X,CCG2,SCAR,SCR10,DXS306,FLJ40595	0	0	0	0	0
Q13131-1	5562	PRKAA1	5'-AMP-activated protein kinase catalytic subunit alpha-1	PRKAA1,MGC57364,MGC33776,AMPKa1,AMPK	0	0	0	0	0
P54619	5571	PRKAG1	5'-AMP-activated protein kinase subunit gamma-1	PRKAG1,AMPKG,MGC8666	0	0	0	0	0
P10809	3329	HSPD1	60 kDa heat shock protein, mitochondrial	HSPD1,HLD4,CPN60,GROEL,HSP60,HSP65,SPG13,HuCHA60	0	0	0	0	0
P10155-1	6738	TROVE2	60 kDa SS-A/Ro ribonucleoprotein	TROVE2,SSA2,RO60	0	0	0	0	0
P35268	6146	RPL22	60S ribosomal protein L22	RPL22,EAP,HBP15,HBP15/L22	0	0	0	0	0
P61513	6168	RPL37A	60S ribosomal protein L37a	RPL37A,MGC74786	0	0	0	0	0
P36639-1	4521	NUDT1	7,8-dihydro-8-oxoguanine triphosphatase	NUDT1,MTH1	0	0	0	0	0
P11021	3309	HSPA5	78 kDa glucose-regulated protein	HSPA5,BIP,MIF2,GRP78,FLJ26106	4	4	4	4	6
Q8IZP0-1	10006	ABI1	Abl interactor 1	ABI1,SSH3BP1,SSH3BP,NAP1BP,ABI-1,E3B1	3	4	4	4	6
P39687	8125	ANP32A	Acidic leucine-rich nuclear phosphoprotein 32 family member A	ANP32A,LANP,MAPM,PP32,PHAP1,PHAPI,I1PP2A,C15orf1,MGC119787,MGC150373	0	0	0	0	0
P60709	60	ACTB	Actin, cytoplasmic 1	ACTB,PS1TP5BP1	2	3	3	3	3
P53999	10923	SUB1	Activated RNA polymerase II transcriptional coactivator p15	SUB1,P15,PC4,p14,MGC102747	3	5	2	4	7
P07741	353	APRT	Adenine phosphoribosyltransferase	APRT,AMP,MGC125856,MGC125857,MGC129961,DKFZp686D13177	0	0	0	0	0
P55263-1	132	ADK	Adenosine kinase	ADK,AK	11	10	12	13	14
P12236	293	SLC25A6	ADP/ATP translocase 3	SLC25A6,AAC3,ANT3,ANT3Y,MGC17525	3	3	2	2	4
Q9UKK9	11164	NUDT5	ADP-sugar pyrophosphatase	NUDT5,YSA1,YSA1H,hYSAH1	3	4	6	5	6
162648 NA	NA		albumin [Bos taurus]	NA	2	2	2	0	3
P06733-1	2023	ENO1	Alpha-enolase	ENO1,ENO1L1,MBP-1,MPB1,PPH,NNE	0	0	0	0	0
Q06203	5471	PPAT	Amidophosphoribosyltransferase	PPAT,GPAT,PRAT,ATASE	11	12	13	11	17
Q12904	9255	AIMP1	Aminoacyl tRNA synthetase complex-interacting multifunctional protein 1	AIMP1,p43,EMAP2,SCYE1,EMAPII	0	0	0	0	0
O95782-1	160	AP2A1	AP-2 complex subunit alpha-1	AP2A1,AP2-ALPHA,CLAPA1,ADTAA	0	0	0	0	0

AC	Entrez_ID	Gene_symbol	Descr	Entrez_GN	pc_Dasatinib_1	pc_Dasatinib_2	[illegible]
Q04446	2632	GBE1	1,4-alpha-glucan-branching enzyme	GBE1,GBE	0	0	[illegible]
P61604	3336	HSPE1	10 kDa heat shock protein, mitochondrial	HSPE1,CPN10,GROES,HSP10	0	0	[illegible]
Q15029	9343	EFTUD2	116 kDa U5 small nuclear ribonucleoprotein component	EFTUD2,Snu114,Snrp116,FLJ44695,KIAA0031,U5-116KD,DKFZp686E24196	0	0	[illegible]
P31946-1	7529	YWHAB	14-3-3 protein beta/alpha	YWHAB,KCIP-1,GW128,HS1	3	2	[illegible]
P62258	7531	YWHAE	14-3-3 protein epsilon	YWHAE,MDS,MDCR,KCIP-1,14-3-3E,FLJ45465	4	6	[illegible]
Q04917	7533	YWHAH	14-3-3 protein eta	YWHAH,YWHA1	3	3	[illegible]
P61981	7532	YWHAG	14-3-3 protein gamma	YWHAG,14-3-3GAMMA	4	4	[illegible]
P31947-1	2810	SFN	14-3-3 protein sigma	SFN,YWHAS	0	2	[illegible]
P27348	10971	YWHAQ	14-3-3 protein theta	YWHAQ,1C5,HS1,14-3-3	3	3	[illegible]
P63104	7534	YWHAZ	14-3-3 protein zeta/delta	YWHAZ,KCIP-1,MGC111427,MGC126532,MGC138156,14-3-3-zeta	6	5	[illegible]
P25398	6206	RPS12	40S ribosomal protein S12	RPS12	0	0	[illegible]
P62263	6208	RPS14	40S ribosomal protein S14	RPS14,EMTB	0	0	[illegible]
P62269	6222	RPS18	40S ribosomal protein S18	RPS18,KE3,HKE3,KE-3,D6S218E,MGC117351,MGC126835,MGC126837	0	0	[illegible]
P39019	6223	RPS19	40S ribosomal protein S19	RPS19,DBA	0	0	[illegible]
P62851	6230	RPS25	40S ribosomal protein S25	RPS25	2	0	[illegible]
P62854	6231	RPS26	40S ribosomal protein S26	RPS26,MGC104292	2	0	[illegible]
P23396	6188	RPS3	40S ribosomal protein S3	RPS3,FLJ26283,FLJ27450,MGC87870	0	0	[illegible]
P62861	NA	FAU	40S ribosomal protein S30	NA	0	0	[illegible]
P62701	6191	RPS4X	40S ribosomal protein S4, X isoform	RPS4X,CCG2,SCAR,SCR10,DXS306,FLJ40595	2	2	[illegible]
P62081	6201	RPS7	40S ribosomal protein S7	RPS7,DBA8	0	0	[illegible]
P08865	3921	RPSA	40S ribosomal protein SA	RPSA,LBP,LRP,p40,67LR,37LRP,LAMBR,LAMR	0	0	[illegible]
P08195-1	6520	SLC3A2	4F2 cell-surface antigen heavy chain	SLC3A2,CD98HC,NACAE,4T2HC,4F2HC,MDU1,CD98,4F2	0	0	[illegible]
Q13131-1	5562	PRKAA1	5'-AMP-activated protein kinase catalytic subunit alpha-1	PRKAA1,MGC57364,MGC33776,AMPKa1,AMPK	0	0	[illegible]
P54619	5571	PRKAG1	5'-AMP-activated protein kinase subunit gamma-1	PRKAG1,AMPKG,MGC8666	0	0	[illegible]
P10809	3329	HSPD1	60 kDa heat shock protein, mitochondrial	HSPD1,HLD4,CPN60,GROEL,HSP60,HSP65,SPG13,HuCHA60	0	0	[illegible]
P10155-1	6738	TROVE2	60 kDa SS-A/Ro ribonucleoprotein	TROVE2,SSA2,RO60	0	0	[illegible]
P35268	6146	RPL22	60S ribosomal protein L22	RPL22,EAP,HBP15,HBP15/L22	0	0	[illegible]
P62750	6147	RPL23A	60S ribosomal protein L23a	RPL23A,MDA20,FLJ27455	0	0	[illegible]
P83881	6173	RPL36A	60S ribosomal protein L36a	RPL36A,L44L,MIG6,RPL44,MGC72020	0	0	[illegible]
P36639-1	4521	NUDT1	7,8-dihydro-8-oxoguanine triphosphatase	NUDT1,MTH1	0	0	[illegible]
P11021	3309	HSPA5	78 kDa glucose-regulated protein	HSPA5,BIP,MIF2,GRP78,FLJ26106	6	8	[illegible]
Q8IZP0-1	10006	ABI1	Abl interactor 1	ABI1,SSH3BP1,SSH3BP,NAP1BP,ABI-1,E3B1	0	0	[illegible]
P24752	38	ACAT1	Acetyl-CoA acetyltransferase, mitochondrial	ACAT1,T2,MAT,ACAT,THIL	0	0	[illegible]
P39687	8125	ANP32A	Acidic leucine-rich nuclear phosphoprotein 32 family member A	ANP32A,LANP,MAPM,PP32,PHAP1,PHAPI,I1PP2A,C15orf1,MGC119787,MGC150373	0	0	[illegible]
P68032	70	ACTC1	Actin, alpha cardiac muscle 1	ACTC1,ACTC,ASD5,CMD1R,CMH11	4	4	[illegible]
P60709	60	ACTB	Actin, cytoplasmic 1	ACTB,PS1TP5BP1	6	6	[illegible]
P61158	10096	ACTR3	Actin-related protein 3	ACTR3,ARP3	0	0	[illegible]
P53999	10923	SUB1	Activated RNA polymerase II transcriptional coactivator p15	SUB1,P15,PC4,p14,MGC102747	3	3	[illegible]
P07741	353	APRT	Adenine phosphoribosyltransferase	APRT,AMP,MGC125856,MGC125857,MGC129961,DKFZp686D13177	0	0	[illegible]
P55263-1	132	ADK	Adenosine kinase	ADK,AK	5	6	[illegible]
Q01518-1	10487	CAP1	Adenylyl cyclase-associated protein 1	CAP1,CAP1-PEN,CAP	0	0	[illegible]
P12236	293	SLC25A6	ADP/ATP translocase 3	SLC25A6,AAC3,ANT3,ANT3Y,MGC17525	2	3	[illegible]
Q9UKK9	11164	NUDT5	ADP-sugar pyrophosphatase	NUDT5,YSA1,YSA1H,hYSAH1	6	6	[illegible]
162648	NA	NA	albumin [Bos taurus]	NA	0	0	[illegible]
P06733-1	2023	ENO1	Alpha-enolase	ENO1,ENO1L1,MBP-1,MPB1,PPH,NNE	0	0	[illegible]
Q06203	5471	PPAT	Amidophosphoribosyltransferase	PPAT,GPAT,PRAT,ATASE	11	10	[illegible]
P04083	301	ANXA1	Annexin A1	ANXA1,ANX1,LPC1	0	0	[illegible]
O95782-1	160	AP2A1	AP-2 complex subunit alpha-1	AP2A1,AP2-ALPHA,CLAPA1,ADTAA	0	0	[illegible]
O94973-1	161	AP2A2	AP-2 complex subunit alpha-2	AP2A2,CLAPA2,ADTAB,HYPJ,HIP9	0	0	[illegible]
P63010-1	163	AP2B1	AP-2 complex subunit beta	AP2B1,DKFZp781K0743,AP2-BETA,CLAPB1,AP105B,ADTB2	0	0	[illegible]
Q96CW1-1	1173	AP2M1	AP-2 complex subunit mu	AP2M1,CLAPM1,AP50,mu2	0	0	[illegible]
P53680-1	1175	AP2S1	AP-2 complex subunit sigma	AP2S1,AP17-DELTA,CLAPS2,AP17	0	0	[illegible]
Q2M2I8	22848	AAK1	AP2-associated protein kinase 1	AAK1,FLJ23712,FLJ25931,FLJ31060,FLJ42882,FLJ45252,KIAA1048,MGC138170,MGC164568,MGC1	0	0	[illegible]
Q92572	1176	AP3S1	AP-3 complex subunit sigma-1	AP3S1,CLAPS3,Sigma3A	0	0	[illegible]
P14868	1615	DARS	Aspartyl-tRNA synthetase, cytoplasmic	DARS,MGC111579,DKFZp781B11202	0	0	[illegible]
P25705	498	ATP5A1	ATP synthase subunit alpha, mitochondrial	ATP5A1,OMR,ORM,ATPM,MOM2,ATP5A,hATP1,ATP5AL2	6	6	[illegible]
P24539	515	ATP5F1	ATP synthase subunit b, mitochondrial	ATP5F1,PIG47,MGC24431	0	0	[illegible]
P06576	506	ATP5B	ATP synthase subunit beta, mitochondrial	ATP5B,ATPMB,ATPSB,MGC5231	2	2	[illegible]
O75947-1	10476	ATP5H	ATP synthase subunit d, mitochondrial	ATP5H,ATP5JD,ATPQ	0	0	[illegible]
P30049	513	ATP5D	ATP synthase subunit delta, mitochondrial	ATP5D	0	0	[illegible]
P56385	521	ATP5I	ATP synthase subunit e, mitochondrial	ATP5I,ATP5K,MGC12532	0	0	[illegible]
O75964	10632	ATP5L	ATP synthase subunit g, mitochondrial	ATP5L,ATP5JG	0	0	[illegible]
P36542-1	509	ATP5C1	ATP synthase subunit gamma, mitochondrial	ATP5C1,ATP5CL1,ATP5C	0	0	[illegible]
P48047	539	ATP5O	ATP synthase subunit O, mitochondrial	ATP5O,ATPO,OSCP	4	4	[illegible]
P12956	2547	XRCC6	ATP-dependent DNA helicase 2 subunit 1	XRCC6,ML8,KU70,TLAA,CTC75,CTCBF,G22P1	0	0	[illegible]
Q86XP3-1	11325	DDX42	ATP-dependent RNA helicase DDX42	DDX42,FLJ43179,SF3B125,RNAHP,RHELP	0	0	[illegible]
bcrabl	NA	NA	BCR-ABL fusion protein	NA	10	9	[illegible]
[illegible]	[illegible]	[illegible]	[illegible]	[illegible]	[illegible]	[illegible]	[illegible]

AC	Entrez_ID	Gene_symbol	Description	Entrez_GN	pc_1	pc_2	pc_grp	sc_1	sc_2	sc_grp	seqcov_1	seqcov_2	seqcov_grp
162648	NA	NA	albumin [Bos taurus]	NA	23	22	26	75	58	133	0.44	0.42	0.51
Q08211	1660	DHX9	ATP-dependent RNA helicase A	DHX9,LKP,RHA,DDX9,NDHI	20	13	22	43	21	64	0.17	0.12	0.19
P17213	671	BPI	Bactericidal permeability-increasing protein	BPI	18	16	19	111	67	178	0.56	0.47	0.57
Q06187	695	BTK	Tyrosine-protein kinase BTK	BTK,AT,ATK,BPK,XLA,IMD1,AGMX1,PSCTK1,MGC126261,MGC126262	15	16	19	53	41	94	0.29	0.32	0.36
P46940	8826	IQGAP1	Ras GTPase-activating-like protein IQGAP1	IQGAP1,SAR1,p195,KIAA0051,HUMORFA01	17	12	18	29	16	45	0.12	0.09	0.13
P22830	2235	FECH	Ferrochelatase, mitochondrial	FECH,EPP,FCE	15	13	17	38	23	61	0.49	0.34	0.49
P42704	10128	LRPPRC	Leucine-rich PPR motif-containing protein, mitochondrial	LRPPRC,LSFC,GP130,LRP130,CLONE-2397	15	9	16	23	11	34	0.13	0.09	0.14
P02788	4057	LTF	Lactotransferrin	LTF,LF,HLF2,GIG12	13	14	16	22	21	43	0.24	0.27	0.31
P20700	4001	LMNB1	Lamin-B1	LMNB1,LMN,ADLD,LMN2,LMNB,MGC111419	12	14	15	29	26	55	0.17	0.21	0.23
Q13724	7841	MOGS	Mannosyl-oligosaccharide glucosidase	MOGS,GCS1	13	12	15	23	20	43	0.22	0.19	0.25
P49748-1	37	ACADVL	Very long-chain specific acyl-CoA dehydrogenase, mitochondrial	ACADVL,VLCAD,LCACD,ACAD6	12	11	14	24	21	45	0.25	0.23	0.28
Q13418	3611	ILK	Integrin-linked protein kinase	ILK,P59,DKFZp686F1765	13	10	13	31	15	46	0.25	0.21	0.25
P10809	3329	HSPD1	60 kDa heat shock protein, mitochondrial	HSPD1,HLD4,CPN60,GROEL,HSP60,HSP65,SPG13,HuCHA6	11	7	12	21	12	33	0.27	0.17	0.29
P41240	1445	CSK	Tyrosine-protein kinase CSK	CSK,MGC117393	10	10	12	38	24	62	0.24	0.25	0.30
P60709	60	ACTB	Actin, cytoplasmic 1	ACTB,PS1TP5BP1	8	9	11	23	34	57	0.31	0.28	0.39
P07948-2	4067	LYN	Tyrosine-protein kinase Lyn	LYN,FLJ26625,JTK8	11	9	11	23	26	49	0.25	0.20	0.25
P22087	2091	FBL	rRNA 2'-O-methyltransferase fibrillarin	FBL,FIB,FLRN,RNU3IP1	6	8	10	13	13	26	0.27	0.32	0.46
P68104	1915	EEF1A1	Elongation factor 1-alpha 1	EEF1A1,CCS3,EF1A,PTI1,CCS-3,EEF-1,EEF1A,EF-Tu,LENG7,eEF1A-1,FLJ25721,GRAF-1EF,MGC16224,MGC102687,MGC131894,HNGC:16303	9	6	9	21	22	43	0.22	0.16	0.22
Q8WWP7	170575	GIMAP1	GTPase IMAP family member 1	GIMAP1,IMAP1,HIMAP1,IMAP38	7	5	9	11	6	17	0.30	0.18	0.36
P61626	4069	LYZ	Lysozyme C	LYZ,LZM,lysozyme	8	9	9	50	34	84	0.61	0.68	0.68
Q99623	11331	PHB2	Prohibitin-2	PHB2,BAP,REA,p22,Bap37,BCAP37,PNAS-141,MGC117268	9	7	9	20	9	29	0.37	0.29	0.37
P68363	10376	TUBA1B	Tubulin alpha-1B chain	TUBA1B,K-ALPHA-1	9	8	9	13	14	27	0.27	0.25	0.27
Q9Y3Z3-1	25939	SAMHD1	SAM domain and HD domain-containing protein 1	SAMHD1,SBBI88,MOP-5,HDDC1,DCIP,AGS5	5	7	9	10	10	20	0.08	0.13	0.16
Q8NI60-1	56997	CABC1	Chaperone activity of bc1 complex-like, mitochondrial	CABC1,MGC4849,SCAR9,ARCA2,ADCK3,COQ8	7	8	9	10	11	21	0.14	0.14	0.16
Q14980-1	4926	NUMA1	Nuclear mitotic apparatus protein 1	NUMA1,NUMA	6	5	9	9	8	17	0.03	0.02	0.04
P40939	3030	HADHA	Trifunctional enzyme subunit alpha, mitochondrial	HADHA,GBP,ECHA,HADH,MTPA,LCHAD,MGC1728,TP-ALPHA	5	5	8	7	7	14	0.07	0.09	0.13
Q9GZT3	81892	C14orf156	SRA stem-loop-interacting RNA-binding protein, mitochondrial	C14orf156,DC50,SLIRP,PD04872	8	7	8	34	17	51	0.75	0.63	0.75
Q9Y6N5	58472	SQRDL	Sulfide:quinone oxidoreductase, mitochondrial	SQRDL,CGI-44	7	4	8	16	9	25	0.21	0.12	0.25
Q8NBX0	51097	SCCPDH	Probable saccharopine dehydrogenase	SCCPDH,NET11,CGI-49,FLJ43187,RP11-439E19.2	7	6	8	12	10	22	0.19	0.20	0.25
Q9UH99	25777	UNC84B	Protein unc-84 homolog B	UNC84B,SUN2,KIAA0668,MGC133055,MGC133056	7	7	8	17	11	28	0.15	0.14	0.17
P50416-1	1374	CPT1A	Carnitine O-palmitoyltransferase 1, liver isoform	CPT1A,L-CPT1,CPT1-L,CPT1	8	5	8	15	7	22	0.11	0.08	0.11
P35232	5245	PHB	Prohibitin	PHB,PHB1	5	6	8	6	7	13	0.28	0.31	0.43
P38919	9775	EIF4A3	Eukaryotic initiation factor 4A-III	EIF4A3,DDX48,NMP265,NUK-34,hNMP265,KIAA0111,MGC10862,eIF4AIII	8	7	8	16	8	24	0.23	0.18	0.23
O43809	11051	NUDT21	Cleavage and polyadenylation specificity factor subunit 5	NUDT21,CPSF5,CFIM25,DKFZp686H1588	7	7	8	20	17	37	0.45	0.48	0.52
Q16539-1	1432	MAPK14	Mitogen-activated protein kinase 14	MAPK14,p38ALPHA,SAPK2A,PRKM15,PRKM14,CSPB1,CSBP2,CSBP1,Mxi2,EXIP,p38,RK	5	7	8	10	10	20	0.13	0.18	0.18
P55084	3032	HADHB	Trifunctional enzyme subunit beta, mitochondrial	HADHB,ECHB,MSTP029,TP-BETA,MGC87480	5	5	7	13	9	22	0.12	0.10	0.17
Q16539-2	1432	MAPK14	Mitogen-activated protein kinase 14	MAPK14,p38ALPHA,SAPK2A,PRKM15,PRKM14,CSPB1,CSBP2,CSBP1,Mxi2,EXIP,p38,RK	4	7	7	7	10	17	0.13	0.18	0.18
P59665	1667.728358	DEFA1B	Neutrophil defensin 1	DEFA1B	7	7	7	180	162	342	0.35	0.35	0.35
P05141	292	SLC25A5	ADP/ATP translocase 2	SLC25A5,T2,T3,2F1,AAC2,ANT2	7	4	7	17	7	24	0.32	0.13	0.32
P07437	203068	TUBB	Tubulin beta chain	TUBB,M40,TUBB1,TUBB5,MGC16435,MGC117247,OK/SW-cl.56	5	5	7	7	6	13	0.18	0.17	0.22
P49411	7284	TUFM	Elongation factor Tu, mitochondrial	TUFM,P43,EFTU,COXPD4,EF-TuMT	5	6	7	9	8	17	0.14	0.17	0.19
P25705	498	ATP5A1	ATP synthase subunit alpha, mitochondrial	ATP5A1,OMR,ORM,ATPM,MOM2,ATP5A,hATP1,ATP5AL2	5	6	7	10	9	19	0.12	0.15	0.16
P23284	5479	PPIB	Peptidyl-prolyl cis-trans isomerase B	PPIB,OI9,CYPB,SCYLP,CYP-S1,MGC2224,MGC14109	7	6	7	13	15	28	0.29	0.25	0.29
P59666	1668	DEFA3	Neutrophil defensin 3	DEFA3,DEF3,HNP3,HP-3,HNP-3	6	7	7	178	162	340	0.35	0.35	0.35
Q03252	84823	LMNB2	Lamin-B2	LMNB2,LMN2,LAMB2,MGC2721	7	4	7	12	9	21	0.09	0.04	0.09
P08311	1511	CTSG	Cathepsin G	CTSG,CG,MGC23078	7	3	7	9	6	15	0.24	0.08	0.24
P12236	293	SLC25A6	ADP/ATP translocase 3	SLC25A6,AAC3,ANT3,ANT3Y,MGC17525	6	3	6	14	6	20	0.27	0.09	0.27
P16402	3007	HIST1H1D	Histone H1.3	HIST1H1D,H1.3,H1F3,MGC13817	6	6	6	42	30	72	0.19	0.19	0.19
P37108	6727	SRP14	Signal recognition particle 14 kDa protein	SRP14,ALURBP,MGC14326	6	6	6	21	13	34	0.41	0.41	0.41
Q8N163-1	57805	KIAA1967	Protein KIAA1967	KIAA1967,NET35,DBC-1,DBC1	4	5	6	4	6	10	0.05	0.05	0.07
P68371	10383	TUBB2C	Tubulin beta-2C chain	TUBB2C,TUBB2	4	4	6	6	5	11	0.15	0.13	0.19
P68366	7277	TUBA4A	Tubulin alpha-4A chain	TUBA4A,TUBA1,FLJ30169,H2-ALPHA	6	4	6	6	7	13	0.18	0.13	0.18
P06239-1	3932	LCK	Proto-oncogene tyrosine-protein kinase LCK	LCK,pp58lck,p56lck,YT16	4	5	6	5	11	16	0.08	0.12	0.14
O60814	85236	HIST1H2BK	Histone H2B type 1-K	HIST1H2BK,H2B/S,H2BFT,H2BFAiii,MGC131989	4	4	5	17	9	26	0.29	0.25	0.34
Q15404	6251	RSU1	Ras suppressor protein 1	RSU1,RSP-1,FLJ31034	5	3	5	13	6	19	0.31	0.16	0.31
P09429	3146	HMGB1	High mobility group protein B1	HMGB1,HMG1,HMG3,SBP-1,DKFZp686A0423	4	4	5	7	8	15	0.22	0.26	0.29
P62081	6201	RPS7	40S ribosomal protein S7	RPS7,DBA8	3	4	5	5	6	11	0.14	0.09	0.15
Q9NVP1	8886	DDX18	ATP-dependent RNA helicase DDX18	DDX18,MrDb,FLJ33908	5	0	5	7	0	7	0.09	0.00	0.09
O60762	8813	DPM1	Dolichol-phosphate mannosyltransferase	DPM1,MPDS,CDGIE	5	2	5	8	2	10	0.28	0.10	0.28
P08246	1991	ELANE	Neutrophil elastase	ELANE,GE,NE,HLE,HNE,ELA2,SCN1,PMN-E	4	4	5	13	15	28	0.10	0.16	0.16
P04843	6184	RPN1	Dolichyl-diphosphooligosaccharide--protein glycosyltransferase subunit 1	RPN1,OST1,RBPH1,DKFZp686B16177	4	2	5	4	2	6	0.10	0.04	0.12
Q92522	8971	H1FX	Histone H1x	H1FX,H1X,MGC8350,MGC15959	5	4	5	8	5	13	0.23	0.23	0.23
P07910-1	3183	HNRNPC	Heterogeneous nuclear ribonucleoproteins C1/C2	HNRNPC,MGC131677,MGC117353,MGC105117,MGC104306,SNRPC,HNRPC,HNRNP,C2,C1	3	5	5	10	17	27	0.09	0.10	0.10
P24158	5657	PRTN3	Myeloblastin	PRTN3,MBN,MBT,NP4,P29,PR3,ACPA,AGP7,PR-3,CANCA,C-ANCA	5	5	5	20	19	39	0.31	0.31	0.31
P09769	2268	FGR	Proto-oncogene tyrosine-protein kinase FGR	FGR,SRC2,c-fgr,c-src2,FLJ43153,MGC75096,p55c-fgr,p58c-fg	4	4	5	7	5	12	0.11	0.11	0.13
O00567	10528	NOP56	Nucleolar protein 56	NOP56,NOL5A	4	5	5	4	6	10	0.07	0.09	0.09
Q86YZ3	388697	HRNR	Hornerin	HRNR,S100A16,S100a18	5	0	5	6	0	6	0.10	0.00	0.10
Q1KMD3	221092	HNRNPUL2	Heterogeneous nuclear ribonucleoprotein U-like protein 2	HNRNPUL2,HNRPUL2,DKFZp762N1910	4	3	5	4	3	7	0.05	0.06	0.09
Q15233	4841	NONO	Non-POU domain-containing octamer-binding protein	NONO,P54,NMT55,NRB54,P54NRB	4	2	5	6	2	8	0.12	0.04	0.13
P62913-1	6135	RPL11	60S ribosomal protein L11	RPL11,GIG34,DBA7	2	4	4	4	7	11	0.10	0.22	0.22
P04233-2	972	CD74	HLA class II histocompatibility antigen gamma chain	CD74,Ia-GAMMA,HLADG,DHLAG	4	0	4	7	0	7	0.20	0.00	0.20
Q00325-1	5250	SLC25A3	Phosphate carrier protein, mitochondrial	SLC25A3,OK/SW-cl.48,PHC	4	4	4	12	9	21	0.09	0.09	0.09
P48059	3987	LIMS1	LIM and senescent cell antigen-like-containing domain protein 1	LIMS1,PINCH,PINCH1	4	2	4	6	3	9	0.14	0.08	0.14
Q99714-1	3028	HSD17B10	3-hydroxyacyl-CoA dehydrogenase type-2	HSD17B10,DUPXp11.22,17b-HSD10,SDR5C1,MRXS10,SCHAD,MRX31,MRX17,MRPP2,HADH2,MHBD,HCD2,ERAB,CAMR,ABAD	2	3	4	3	3	6	0.16	0.19	0.26
Q9HBI0-1	64098	PARVG	Gamma-parvin	PARVG	4	2	4	9	5	14	0.14	0.07	0.14
Q9Y3E5	51651	PTRH2	Peptidyl-tRNA hydrolase 2, mitochondrial	PTRH2,BIT1,PTH2,CGI-147,FLJ32471	2	3	4	3	3	6	0.16	0.25	0.33
P26368-1	11338	U2AF2	Splicing factor U2AF 65 kDa subunit	U2AF2,U2AF65	4	3	4	8	3	11	0.09	0.07	0.09
Q96AG4	55379	LRRC59	Leucine-rich repeat-containing protein 59	LRRC59,PRO1855,FLJ21675	2	2	4	2	2	4	0.08	0.09	0.18
O14976	2580	GAK	Cyclin G-associated kinase	GAK,FLJ16629,FLJ40395,MGC99654	4	0	4	4	0	4	0.04	0.00	0.04
O75367-1	9555	H2AFY	Core histone macro-H2A.1	H2AFY,macroH2A1.2,MACROH2A1.1,H2AF12M,mH2A1,H2AFJ,H2A/y,H2A.y	4	0	4	9	0	9	0.13	0.00	0.13
Q9HCU5	10113	PREB	Prolactin regulatory element-binding protein	PREB,SEC12,MGC3467	3	3	4	5	5	10	0.11	0.09	0.15
P20160	566	AZU1	Azurocidin	AZU1,AZU,HBP,NAZC,AZAMP,CAP37,HUMAZUR	4	3	4	8	6	14	0.16	0.12	0.16
P12724	6037	RNASE3	Eosinophil cationic protein	RNASE3,ECP,RNS3	4	3	4	12	7	19	0.24	0.17	0.24
P53007	6576	SLC25A1	Tricarboxylate transport protein, mitochondrial	SLC25A1,CTP,SLC20A3	4	0	4	4	0	4	0.13	0.00	0.13
136429	NA	NA	trypsin [Sus scrofa]	NA	4	4	4	119	95	214	0.24	0.24	0.24
P23246-1	6421	SFPQ	Splicing factor, proline- and glutamine-rich	SFPQ,POMP100,PSF	3	2	3	4	4	8	0.06	0.03	0.06
P35268	6146	RPL22	60S ribosomal protein L22	RPL22,EAP,HBP15,HBP15/L22	0	3	3	0	5	5	0.00	0.30	0.30
P08631-1	3055	HCK	Tyrosine-protein kinase HCK	HCK,JTK9	3	2	3	5	6	11	0.06	0.03	0.06
P51571	6748	SSR4	Translocon-associated protein subunit delta	SSR4,TRAPD	3	0	3	3	0	3	0.23	0.00	0.23
Q8IV04	374403	TBC1D10C	Carabin	TBC1D10C,CARABIN,FLJ00332,MGC46488	3	0	3	3	0	3	0.11	0.00	0.11
O00422	10284	SAP18	Histone deacetylase complex subunit SAP18	SAP18,SAP18P,2HOR0202,MGC27131	3	2	3	5	4	9	0.21	0.15	0.21
P04839	1536	CYBB	Cytochrome b-245 heavy chain	CYBB,CGD,NOX2,GP91-1,GP91PHOX,p91-PHOX,GP91-PHOX	2	3	3	2	3	5	0.05	0.06	0.06
Q8TAQ2-1	6601	SMARCC2	SWI/SNF complex subunit SMARCC2	SMARCC2,CRACC2,BAF170,Rsc8	2	2	3	2	2	4	0.02	0.02	0.03
P61326	4116	MAGOH	Protein mago nashi homolog	MAGOH,MAGOHA	2	2	3	2	3	5	0.19	0.14	0.27
P49458	653226.6726	SRP9	Signal recognition particle 9 kDa protein	SRP9,ALURBP,MGC110981	3	3	3	11	6	17	0.36	0.36	0.36
Q9HBI1	29780	PARVB	Beta-parvin	PARVB,CGI-56	2	2	3	3	3	6	0.08	0.08	0.12
P62826	5901	RAN	GTP-binding nuclear protein Ran	RAN,TC4,Gsp1,ARA24	2	2	3	4	3	7	0.09	0.08	0.13
Q08J23	54888	NSUN2	tRNA (cytosine-5-)-methyltransferase NSUN2	NSUN2,MISU,SAKI,TRM4,FLJ20303	3	2	3	4	2	6	0.05	0.03	0.05
Q96GQ7	55661	DDX27	Probable ATP-dependent RNA helicase DDX27	DDX27,RHLP,Rrp3p,PP3241,HSPC259,MGC1018,FLJ12917,FLJ20596,FLJ22238,MGC163147,dJ686N3.1,DKFZp667N05	3	0	3	3	0	3	0.05	0.00	0.05
P17844	1655	DDX5	Probable ATP-dependent RNA helicase DDX5	DDX5,p68,HLR1,G17P1,HUMP68,DKFZp434E109,DKFZp686J0119	0	3	3	0	3	3	0.00	0.06	0.06
P27707	1633	DCK	Deoxycytidine kinase	DCK,MGC117410,MGC138632	3	0	3	5	0	5	0.12	0.00	0.12
P28799-1	2896	GRN	Granulins	GRN,PCDGF,PGRN,PEPI,GP88,GEP	3	2	3	5	2	7	0.06	0.05	0.06
P16401	3009	HIST1H1B	Histone H1.5	HIST1H1B,H1,H1.5,H1F5,MGC126630,MGC12663	3	2	3	11	10	21	0.15	0.10	0.15
P55769	4809	NHP2L1	NHP2-like protein 1	NHP2L1,FA1,FA-1,NHPX,15.5K,OTK27,SNU13,SSFA1,SPAG12,SNRNP15-	3	0	3	5	0	5	0.30	0.00	0.30
P62829	9349	RPL23	60S ribosomal protein L23	RPL23,rpL17,MGC72008,MGC111167,MGC117346	3	3	3	4	4	8	0.28	0.28	0.28
O94826	9868	TOMM70A	Mitochondrial import receptor subunit TOM70	TOMM70A,FLJ90470	2	3	3	2	3	5	0.04	0.06	0.06
Q8NB16-1	197259	MLKL	Mixed lineage kinase domain-like protein	MLKL,FLJ34389	3	0	3	3	0	3	0.08	0.00	0.08
P53999	10923	SUB1	Activated RNA polymerase II transcriptional coactivator p15	SUB1,P15,PC4,p14,MGC102747	3	0	3	8	0	8	0.27	0.00	0.27
P06576	506	ATP5B	ATP synthase subunit beta, mitochondrial	ATP5B,ATPMB,ATPSB,MGC5231	3	2	3	4	2	6	0.09	0.06	0.09
Q5JTV8-1	26092	TOR1AIP1	Torsin-1A-interacting protein 1	TOR1AIP1,RP11-533E19.1,DKFZp586G011,FLJ13142,MGC3413,LAP1	0	2	2	0	2	2	0.00	0.04	0.04
O60506-1	10492	SYNCRIP	Heterogeneous nuclear ribonucleoprotein Q	SYNCRIP,dJ3J17.2,FLJ31626,hnRNP-Q,GRY-RBP,HNRPQ1,GRYRBP,NSAP1,PP68	2	0	2	2	0	2	0.04	0.00	0.04
P14136-1	2670	GFAP	Glial fibrillary acidic protein	GFAP,FLJ45472	2	0	2	18	0	18	0.04	0.00	0.04
P08473	4311	MME	Neprilysin	MME,NEP,CD10,CALLA,MGC126681,MGC126707,DKFZp686O16152	2	2	2	2	2	4	0.03	0.03	0.03
Q9H3N1	81542	TMX1	Thioredoxin-related transmembrane protein 1	TMX1,TMX,TXNDC,PDIA11,TXNDC1,DKFZp564E1962	0	2	2	0	2	2	0.00	0.09	0.09
Q14004-1	8621	CDK13	Cell division cycle 2-like protein kinase 5	CDK13,KIAA1791,FLJ35215,CDC2L5,CDC2L,CHED	2	0	2	3	0	3	0.01	0.00	0.01
Q14152	8661	EIF3A	Eukaryotic translation initiation factor 3 subunit A	EIF3A,EIF3,P167,p180,p185,TIF32,EIF3S10,KIAA0139,eIF3-p170,eIF3-theta	2	0	2	3	0	3	0.02	0.00	0.02
Q9H0E3-1	79595	SAP130	Histone deacetylase complex subunit SAP130	SAP130,FLJ12761	0	2	2	0	2	2	0.00	0.02	0.02
P07305	3005	H1F0	Histone H1.0	H1F0,H10,H1FV,MGC5241	2	0	2	4	0	4	0.10	0.00	0.10
O75533	23451	SF3B1	Splicing factor 3B subunit 1	SF3B1,PRP10,PRPF10,SAP155,SF3b155	2	0	2	2	0	2	0.02	0.00	0.02
Q8WY22	140707	BRI3BP	BRI3-binding protein	BRI3BP,KG19,BNAS1,HCCR-2	2	2	2	2	4	6	0.14	0.14	0.14
P09874	142	PARP1	Poly [ADP-ribose] polymerase 1	PARP1,PARP,PPOL,ADPRT,ADPRT1,PARP-1,pADPRT-1	0	2	2	0	3	3	0.00	0.03	0.03
P54803-1	2581	GALC	Galactocerebrosidase	GALC	2	0	2	2	0	2	0.05	0.00	0.05
P42285	23517	SKIV2L2	Superkiller viralicidic activity 2-like 2	SKIV2L2,Dob1,Mtr4,fSAP118,KIAA0052,MGC142069	0	2	2	0	2	2	0.00	0.02	0.02
P43405-1	6850	SYK	Tyrosine-protein kinase SYK	SYK,DKFZp313N1010,FLJ37489,FLJ2504	0	2	2	0	3	3	0.00	0.03	0.03
P60228	3646	EIF3E	Eukaryotic translation initiation factor 3 subunit E	EIF3E,INT6,EIF3S6,EIF3-P48,eIF3-p46	0	2	2	0	2	2	0.00	0.04	0.04
Q99442	7095	SEC62	Translocation protein SEC62	SEC62,HTP1,TP-1,Dtrp1,TLOC1,FLJ32803	2	2	2	4	2	6	0.06	0.06	0.06
P17480-1	7343	UBTF	Nucleolar transcription factor 1	UBTF,NOR-90,UBF	0	2	2	0	3	3	0.00	0.03	0.03
Q96CW1-1	1173	AP2M1	AP-2 complex subunit mu	AP2M1,CLAPM1,AP50,mu2	2	0	2	2	0	2	0.04	0.00	0.04
P32969	6133	RPL9	60S ribosomal protein L9	RPL9,FLJ27456,MGC15545,NPC-A-16,DKFZp313J151	2	0	2	2	0	2	0.14	0.00	0.14
Q9H078-1	81570	CLPB	Caseinolytic peptidase B protein homolog	CLPB,FLJ13152,HSP78,SKD	2	0	2	2	0	2	0.04	0.00	0.04
P02042	3045	HBD	Hemoglobin subunit delta	HBD	2	0	2	2	0	2	0.15	0.00	0.15
P52298-1	22916	NCBP2	Nuclear cap-binding protein subunit 2	NCBP2,PIG55,CBP20,NIP1,CBC2	0	2	2	0	2	2	0.00	0.13	0.13
P01024	718	C3	Complement C3	C3,ASP,AHUS5,ARMD9,CPAMD1	0	2	2	0	3	3	0.00	0.01	0.01
Q9BQG0-1	10514	MYBBP1A	Myb-binding protein 1A	MYBBP1A,FLJ37886,PAP2,P160	0	2	2	0	3	3	0.00	0.02	0.02
P04899-1	2771	GNAI2	Guanine nucleotide-binding protein G(i), alpha-2 subunit	GNAI2,H_LUCA16.1,H_LUCA15.1,GNAI2B,GIP	2	0	2	2	0	2	0.07	0.00	0.07
Q9BVC6	79073	TMEM109	Transmembrane protein 109	TMEM109,MGC5508	2	0	2	4	0	4	0.09	0.00	0.09
P08670	7431	VIM	Vimentin	VIM,FLJ36605	2	0	2	4	0	4	0.04	0.00	0.04
Q16630-1	11052	CPSF6	Cleavage and polyadenylation specificity factor subunit 6	CPSF6,HPBRII-7,HPBRII-4,CFIM68,CFIM	2	0	2	2	0	2	0.05	0.00	0.05
P01903	3122	HLA-DRA	HLA class II histocompatibility antigen, DR alpha chain	HLA-DRA,HLA-DRA1	2	0	2	5	0	5	0.11	0.00	0.11
P00519-1	25	ABL1	Proto-oncogene tyrosine-protein kinase ABL1	ABL1,bcr/abl,v-abl,c-ABL,p150,JTK7,ABL	0	2	2	0	2	2	0.00	0.03	0.03
Q9Y5M8	58477	SRPRB	Signal recognition particle receptor subunit beta	SRPRB,APMCF1	2	0	2	2	0	2	0.10	0.00	0.10
Q00610-1	1213	CLTC	Clathrin heavy chain 1	CLTC,KIAA0034,CLTCL2,CLH-17,CHC17,CHC,Hc	2	0	2	2	0	2	0.01	0.00	0.01
P62277	6207	RPS13	40S ribosomal protein S13	RPS13	2	2	2	2	4	6	0.15	0.15	0.15
P61353	6155	RPL27	60S ribosomal protein L27	RPL27	0	2	2	0	3	3	0.00	0.10	0.10
O43390-1	10236	HNRNPR	Heterogeneous nuclear ribonucleoprotein R	HNRNPR,FLJ25714,hnRNP-R,HNRPR	2	0	2	2	0	2	0.04	0.00	0.04
Q9H4G4	152007	GLIPR2	Golgi-associated plant pathogenesis-related protein 1	GLIPR2,GAPR-1,C9orf19	0	2	2	0	2	2	0.00	0.17	0.17
Q9Y4P3	26608	TBL2	Transducin beta-like protein 2	TBL2,WBSCR13,MGC134739,WS-betaTRP,DKFZp434N024	2	0	2	2	0	2	0.05	0.00	0.05
O75323	2631	GBAS	Protein NipSnap homolog 2	GBAS,NIPSNAP2	0	2	2	0	2	2	0.00	0.09	0.09
P04908	3012.8335	HIST1H2AB	Histone H2A type 1-B/E	HIST1H2AB,H2A/m,H2AFM	2	0	2	12	0	12	0.29	0.00	0.29
Q9Y2U5	10746	MAP3K2	Mitogen-activated protein kinase kinase kinase 2	MAP3K2,MEKK2,MEKK2B	0	2	2	0	2	2	0.00	0.03	0.03
Q92925-1	6603	SMARCD2	SWI/SNF-related matrix-associated actin-dependent regulator of chromatin subfamily D member 2	SMARCD2,PRO2451,CRACD2,BAF60B,Rsc6p	0	2	2	0	2	2	0.00	0.04	0.04
O14602	9086	EIF1AY	Eukaryotic translation initiation factor 1A, Y-chromosomal	EIF1AY	0	2	2	0	4	4	0.00	0.15	0.15
P42680	7006	TEC	Tyrosine-protein kinase Tec	TEC,PSCTK4,MGC126760,MGC126762	2	0	2	2	0	2	0.03	0.00	0.03
P84090	2079	ERH	Enhancer of rudimentary homolog	ERH,DROER,FLJ27340	2	0	2	2	0	2	0.14	0.00	0.14
Q16718	4698	NDUFA5	NADH dehydrogenase [ubiquinone] 1 alpha subcomplex subunit 5	NDUFA5,B13,NUFM,UQOR13,FLJ12147,CI-13KD-B,DKFZp781K135	2	0	2	2	0	2	0.17	0.00	0.17
Q15910	NA	EZH2	Histone-lysine N-methyltransferase EZH2	NA	0	2	2	0	2	2	0.00	0.04	0.04
P05164-1	4353	MPO	Myeloperoxidase	MPO	2	0	2	2	0	2	0.04	0.00	0.04
O14773-1	1200	TPP1	Tripeptidyl-peptidase 1	TPP1,MGC21297,LPIC,GIG1,CLN2	2	2	2	2	2	4	0.06	0.06	0.06
Q9Y2X3	51602	NOP58	Nucleolar protein 58	NOP58,NOP5,HSPC120,NOP5/NOP58	2	0	2	3	0	3	0.06	0.00	0.06
Q13576-1	10788	IQGAP2	Ras GTPase-activating-like protein IQGAP2	IQGAP2	2	2	2	2	2	4	0.02	0.02	0.02

Drug	Kinase	IC_{50} [nM]
Bosutinib	MAP2K1 (MEK1)	2480
Bosutinib	MAP2K2 (MEK2)	143
Bosutinib	MAP3K2 (MEKK2)	68
Nilotinib	MAPK9 (JNK2)	12900

AC	Entrez_ID	Gene_symbol	Descr	Entrez_GN	pc_Dasatinib_1	pc_Dasatinib_2	pc_Dasatinib_grp	pc_Bosutinib_1	pc_Bosutinib_2	pc_Bosutinib_grp	pc_Bafetinib_1	pc_Bafetinib_2	pc_Bafetinib_grp	pc_Nilotinib_1	pc_Nilotinib_2	pc_Nilotinib_grp	sc_Dasatinib_1	sc_Dasatinib_2	sc_Dasatinib_grp	sc_Dasatinib_mean	sc_Bosutinib_1	sc_Bosutinib_2	sc_Bosutinib_grp	sc_Bosutinib_mean	sc_Bafetinib_1	sc_Bafetinib_2	sc_Bafetinib_grp	sc_Bafetinib_mean	sc_Nilotinib_1	sc_Nilotinib_2	sc_Nilotinib_grp	sc_Nilotinib_mean	seqcov_Dasatinib_1	seqcov_Dasatinib_2	seqcov_Dasatinib_grp	seqcov_Bosutinib_1	seqcov_Bosutinib_2	seqcov_Bosutinib_grp	seqcov_Bafetinib_1	seqcov_Bafetinib_2	seqcov_Bafetinib_grp	seqcov_Nilotinib_1	seqcov_Nilotinib_2	seqcov_Nilotinib_grp
Q04446	2632	GBE1	1,4-alpha-glucan-branching enzyme	GBE1,GBE	3	2	5	5	7	8	3	3	3	4	5	6	3.00	2.00	5.00	2.50	11.00	13.00	24.00	12.00	7.00	6.00	13.00	6.50	5.00	7.00	12.00	6.00	0.05	0.03	0.08	0.09	0.12	0.14	0.06	0.06	0.06	0.07	0.10	0.11
P61604	3336	HSPE1	10 kDa heat shock protein, mitochondrial	HSPE1,CPN10,GROES,HSP10	0	0	0	0	0	0	0	0	0	0	0	0	0.00	0.00	0.00	0.00	0.00	0.00	0.00	0.00	0.00	0.00	0.00	0.00	0.00	0.00	0.00	0.00	0.00	0.00	0.00	0.00	0.00	0.00	0.00	0.00	0.00	0.00	0.00	0.00
P31946-1	7529	YWHAB	14-3-3 protein beta/alpha	YWHAB,KCIP-1,GW128,HS1	3	0	3	4	4	4	0	0	0	0	0	0	6.00	0.00	6.00	3.00	13.00	10.00	23.00	11.50	0.00	0.00	0.00	0.00	0.00	0.00	0.00	0.00	0.12	0.00	0.12	0.15	0.15	0.15	0.00	0.00	0.00	0.00	0.00	0.00
P62258	7531	YWHAE	14-3-3 protein epsilon	YWHAE,MDS,MDCR,KCIP-1,14-3-3E,FLJ45465	3	2	3	6	5	6	3	2	3	3	2	3	8.00	8.00	16.00	8.00	13.00	15.00	28.00	14.00	7.00	7.00	14.00	7.00	7.00	6.00	13.00	6.50	0.12	0.07	0.12	0.24	0.19	0.24	0.12	0.07	0.12	0.12	0.07	0.12
Q04917	7533	YWHAH	14-3-3 protein eta	YWHAH,YWHA1	3	0	3	4	3	4	0	0	0	0	0	0	6.00	0.00	6.00	3.00	13.00	9.00	22.00	11.00	0.00	0.00	0.00	0.00	0.00	0.00	0.00	0.00	0.12	0.00	0.12	0.15	0.09	0.15	0.00	0.00	0.00	0.00	0.00	0.00
P61981	7532	YWHAG	14-3-3 protein gamma	YWHAG,14-3-3GAMMA	4	2	4	6	5	6	3	2	3	2	2	2	7.00	8.00	15.00	7.50	20.00	12.00	32.00	16.00	10.00	7.00	17.00	8.50	7.00	6.00	13.00	6.50	0.16	0.09	0.16	0.27	0.19	0.27	0.12	0.09	0.12	0.09	0.09	0.09
P31947-1	2810	SFN	14-3-3 protein sigma	SFN,YWHAS	2	0	2	3	3	3	0	0	0	0	0	0	5.00	0.00	5.00	2.50	12.00	9.00	21.00	10.50	0.00	0.00	0.00	0.00	0.00	0.00	0.00	0.00	0.06	0.00	0.06	0.09	0.09	0.09	0.00	0.00	0.00	0.00	0.00	0.00
P27348	10971	YWHAQ	14-3-3 protein theta	YWHAQ,1C5,HS1,14-3-3	3	2	3	5	4	5	0	0	0	0	2	2	6.00	9.00	15.00	7.50	14.00	10.00	24.00	12.00	0.00	0.00	0.00	0.00	0.00	4.00	4.00	2.00	0.11	0.08	0.11	0.18	0.13	0.18	0.00	0.00	0.00	0.00	0.08	0.08
P63104	7534	YWHAZ	14-3-3 protein zeta/delta	YWHAZ,KCIP-1,MGC111427,MGC126532,MGC138156,14-3-3-zeta	5	4	5	7	6	7	4	4	4	4	4	4	8.00	10.00	18.00	9.00	16.00	18.00	34.00	17.00	9.00	10.00	19.00	9.50	7.00	7.00	14.00	7.00	0.26	0.21	0.26	0.34	0.29	0.34	0.21	0.21	0.21	0.21	0.21	0.21
P62263	6208	RPS14	40S ribosomal protein S14	RPS14,EMTB	0	0	0	0	0	0	0	2	2	0	0	0	0.00	0.00	0.00	0.00	0.00	0.00	0.00	0.00	0.00	4.00	4.00	2.00	0.00	0.00	0.00	0.00	0.00	0.00	0.00	0.00	0.00	0.00	0.00	0.16	0.16	0.00	0.00	0.00
P62269	6222	RPS18	40S ribosomal protein S18	RPS18,KE3,HKE3,KE-3,D6S218E,MGC117351,MGC126835,MGC126837	0	2	2	0	0	0	2	0	2	0	0	0	0.00	3.00	3.00	1.50	0.00	0.00	0.00	0.00	4.00	0.00	4.00	2.00	0.00	0.00	0.00	0.00	0.00	0.12	0.12	0.00	0.00	0.00	0.12	0.00	0.12	0.00	0.00	0.00
P39019	6223	RPS19	40S ribosomal protein S19	RPS19,DBA	0	0	0	0	0	0	0	0	0	0	0	0	0.00	0.00	0.00	0.00	0.00	0.00	0.00	0.00	0.00	0.00	0.00	0.00	0.00	0.00	0.00	0.00	0.00	0.00	0.00	0.00	0.00	0.00	0.00	0.00	0.00	0.00	0.00	0.00
P62851	6230	RPS25	40S ribosomal protein S25	RPS25	2	0	2	2	2	2	2	2	2	3	3	3	13.00	0.00	13.00	6.50	12.00	9.00	21.00	10.50	16.00	17.00	33.00	16.50	14.00	15.00	29.00	14.50	0.15	0.00	0.15	0.15	0.15	0.15	0.15	0.15	0.15	0.24	0.24	0.24
P62854	6231	RPS26	40S ribosomal protein S26	RPS26,MGC104292	3	2	3	2	0	2	3	2	3	3	2	3	4.00	3.00	7.00	3.50	2.00	0.00	2.00	1.00	3.00	2.00	5.00	2.50	5.00	3.00	8.00	4.00	0.27	0.21	0.27	0.14	0.00	0.14	0.27	0.14	0.27	0.27	0.14	0.27
P23396	6188	RPS3	40S ribosomal protein S3	RPS3,FLJ26283,FLJ27450,MGC87870	0	0	0	0	0	0	2	3	4	0	0	0	0.00	0.00	0.00	0.00	0.00	0.00	0.00	0.00	2.00	3.00	5.00	2.50	0.00	0.00	0.00	0.00	0.00	0.00	0.00	0.00	0.00	0.00	0.10	0.14	0.19	0.00	0.00	0.00
P61247	6189	RPS3A	40S ribosomal protein S3a	RPS3A,FTE1,MFTL,MGC23240	0	0	0	0	0	0	0	0	0	3	0	3	0.00	0.00	0.00	0.00	0.00	0.00	0.00	0.00	0.00	0.00	0.00	0.00	3.00	0.00	3.00	1.50	0.00	0.00	0.00	0.00	0.00	0.00	0.00	0.00	0.00	0.11	0.00	0.11
P62701	6191	RPS4X	40S ribosomal protein S4, X isoform	RPS4X,CCG2,SCAR,SCR10,DXS306,FLJ40595	0	0	0	0	0	0	2	0	2	3	3	4	0.00	0.00	0.00	0.00	0.00	0.00	0.00	0.00	2.00	0.00	2.00	1.00	4.00	5.00	9.00	4.50	0.00	0.00	0.00	0.00	0.00	0.00	0.07	0.00	0.07	0.12	0.12	0.16
Q13131-1	5562	PRKAA1	5'-AMP-activated protein kinase catalytic subunit alpha-1	PRKAA1,MGC57364,MGC33776,AMPKa1,AMPK	0	0	0	6	6	7	0	0	0	0	0	0	0.00	0.00	0.00	0.00	20.00	22.00	42.00	21.00	0.00	0.00	0.00	0.00	0.00	0.00	0.00	0.00	0.00	0.00	0.00	0.14	0.12	0.15	0.00	0.00	0.00	0.00	0.00	0.00
P54619	5571	PRKAG1	5'-AMP-activated protein kinase subunit gamma-1	PRKAG1,AMPKG,MGC8666	0	0	0	6	5	7	0	0	0	0	0	0	0.00	0.00	0.00	0.00	15.00	9.00	24.00	12.00	0.00	0.00	0.00	0.00	0.00	0.00	0.00	0.00	0.00	0.00	0.00	0.18	0.18	0.24	0.00	0.00	0.00	0.00	0.00	0.00
P10809	3329	HSPD1	60 kDa heat shock protein, mitochondrial	HSPD1,HLD4,CPN60,GROEL,HSP60,HSP65,SPG13,HuCHA60	4	3	4	3	2	3	3	3	4	5	4	5	4.00	4.00	8.00	4.00	3.00	3.00	6.00	3.00	3.00	7.00	10.00	5.00	7.00	5.00	12.00	6.00	0.11	0.07	0.11	0.07	0.04	0.07	0.07	0.06	0.08	0.14	0.11	0.14
P10155-1	6738	TROVE2	60 kDa SS-A/Ro ribonucleoprotein	TROVE2,SSA2,RO60	0	0	0	0	0	0	0	0	0	0	0	0	0.00	0.00	0.00	0.00	0.00	0.00	0.00	0.00	0.00	0.00	0.00	0.00	0.00	0.00	0.00	0.00	0.00	0.00	0.00	0.00	0.00	0.00	0.00	0.00	0.00	0.00	0.00	0.00
P35268	6146	RPL22	60S ribosomal protein L22	RPL22,EAP,HBP15,HBP15/L22	2	0	2	0	2	2	2	2	2	2	2	2	4.00	0.00	4.00	2.00	0.00	4.00	4.00	2.00	6.00	5.00	11.00	5.50	5.00	7.00	12.00	6.00	0.20	0.00	0.20	0.00	0.20	0.20	0.20	0.20	0.20	0.20	0.20	0.20
P61513	6168	RPL37A	60S ribosomal protein L37a	RPL37A,MGC74786	0	0	0	0	0	0	0	0	0	0	0	0	0.00	0.00	0.00	0.00	0.00	0.00	0.00	0.00	0.00	0.00	0.00	0.00	0.00	0.00	0.00	0.00	0.00	0.00	0.00	0.00	0.00	0.00	0.00	0.00	0.00	0.00	0.00	0.00
P36639-1	4521	NUDT1	7,8-dihydro-8-oxoguanine triphosphatase	NUDT1,MTH1	0	0	0	4	3	4	0	0	0	0	0	0	0.00	0.00	0.00	0.00	6.00	9.00	15.00	7.50	0.00	0.00	0.00	0.00	0.00	0.00	0.00	0.00	0.00	0.00	0.00	0.26	0.20	0.26	0.00	0.00	0.00	0.00	0.00	0.00
P11021	3309	HSPA5	78 kDa glucose-regulated protein	HSPA5,BIP,MIF2,GRP78,FLJ26106	3	4	4	6	8	9	3	5	5	4	7	7	7.00	5.00	12.00	6.00	17.00	23.00	40.00	20.00	11.00	14.00	25.00	12.50	7.00	19.00	26.00	13.00	0.06	0.08	0.08	0.12	0.18	0.19	0.06	0.11	0.11	0.09	0.14	0.14
Q8IZP0-1	10006	ABI1	Abl interactor 1	ABI1,SSH3BP1,SSH3BP,NAP1BP,ABI-1,E3B1	0	0	0	0	0	0	0	0	0	0	0	0	0.00	0.00	0.00	0.00	0.00	0.00	0.00	0.00	0.00	0.00	0.00	0.00	0.00	0.00	0.00	0.00	0.00	0.00	0.00	0.00	0.00	0.00	0.00	0.00	0.00	0.00	0.00	0.00
P39687	8125	ANP32A	Acidic leucine-rich nuclear phosphoprotein 32 family member A	ANP32A,LANP,MAPM,PP32,PHAP1,PHAPI,I1PP2A,C15orf1,MGC119787,MGC150373	0	0	0	2	2	2	0	0	0	0	0	0	0.00	0.00	0.00	0.00	4.00	5.00	9.00	4.50	0.00	0.00	0.00	0.00	0.00	0.00	0.00	0.00	0.00	0.00	0.00	0.08	0.08	0.08	0.00	0.00	0.00	0.00	0.00	0.00
[illegible]	[illegible]	[illegible]	[illegible]	[illegible]	[illegible]	[illegible]	[illegible]	[illegible]	[illegible]	[illegible]	[illegible]	[illegible]	[illegible]	[illegible]	[illegible]	[illegible]	[illegible]	[illegible]	[illegible]	[illegible]	[illegible]	[illegible]	[illegible]	[illegible]	[illegible]	[illegible]	[illegible]	[illegible]	[illegible]	[illegible]	[illegible]	[illegible]	[illegible]	[illegible]	[illegible]	[illegible]	[illegible]	[illegible]	[illegible]	[illegible]	[illegible]	[illegible]	[illegible]	[illegible]

AC	Entrez_ID	Gene_symbol	Descr	Entrez_GN	pc_Dasatinib_1	pc_Dasatinib_2	pc_Dasatinib_grp	pc_Bosutinib_1	pc_Bosutinib_2	pc_Bosutinib_grp	pc_Bafetinib_1	pc_Bafetinib_2	pc_Bafetinib_grp	pc_Nilotinib_1	pc_Nilotinib_2	pc_Nilotinib_grp	sc_Dasatinib_1	sc_Dasatinib_2	sc_Dasatinib_grp	sc_Dasatinib_mean	sc_Bosutinib_1	sc_Bosutinib_2	sc_Bosutinib_grp	sc_Bosutinib_mean	sc_Bafetinib_1	sc_Bafetinib_2	sc_Bafetinib_grp	sc_Bafetinib_mean	sc_Nilotinib_1	sc_Nilotinib_2	sc_Nilotinib_grp	sc_Nilotinib_mean	seqcov_Dasatinib_1	seqcov_Dasatinib_2	seqcov_Dasatinib_grp	seqcov_Bosutinib_1	seqcov_Bosutinib_2	seqcov_Bosutinib_grp	seqcov_Bafetinib_1	seqcov_Bafetinib_2	seqcov_Bafetinib_grp	seqcov_Nilotinib_1	seqcov_Nilotinib_2	seqcov_Nilotinib_grp
Q04446	2632	GBE1	1,4-alpha-glucan-branching enzyme	GBE1,GBE	0	0	0	4	0	4	4	3	4	0	0	0	0	0	0	0	5	0	5	2.5	4	3	7	3.5	0	0	0	0	0.00	0.00	0.00	0.07	0.00	0.07	0.08	0.06	0.08	0.00	0.00	0.00
P61604	3336	HSPE1	10 kDa heat shock protein, mitochondrial	HSPE1,CPN10,GROES,HSP10	0	0	0	0	0	0	5	5	6	0	0	0	0	0	0	0	0	0	0	0	17	10	27	13.5	0	0	0	0	0.00	0.00	0.00	0.00	0.00	0.00	0.44	0.47	0.54	0.00	0.00	0.00
Q15029	9343	EFTUD2	116 kDa U5 small nuclear ribonucleoprotein component	EFTUD2,Snu114,Snrp116,FLJ44695,KIAA0031,U5-116KD,DKFZp686E24196	0	0	0	2	0	2	0	0	0	0	0	0	0	0	0	0	2	0	2	1	0	0	0	0	0	0	0	0	0.00	0.00	0.00	0.03	0.00	0.03	0.00	0.00	0.00	0.00	0.00	0.00
P31946-1	7529	YWHAB	14-3-3 protein beta/alpha	YWHAB,KCIP-1,GW128,HS1	0	0	0	0	0	0	3	3	3	0	0	0	0	0	0	0	0	0	0	0	8	13	21	10.5	0	0	0	0	0.00	0.00	0.00	0.00	0.00	0.00	0.12	0.12	0.12	0.00	0.00	0.00
P62258	7531	YWHAE	14-3-3 protein epsilon	YWHAE,MDS,MDCR,KCIP-1,14-3-3E,FLJ45465	4	2	4	3	2	3	5	4	5	0	2	2	10	7	17	8.5	12	10	22	11	14	15	29	14.5	0	5	5	2.5	0.17	0.07	0.17	0.12	0.07	0.12	0.21	0.16	0.21	0.00	0.07	0.07
Q04917	7533	YWHAH	14-3-3 protein eta	YWHAH,YWHA1	0	2	2	0	0	0	3	2	3	0	0	0	0	4	4	2	0	0	0	0	8	11	19	9.5	0	0	0	0	0.00	0.09	0.09	0.00	0.00	0.00	0.12	0.07	0.12	0.00	0.00	0.00
P61981	7532	YWHAG	14-3-3 protein gamma	YWHAG,14-3-3GAMMA	4	3	4	3	3	3	4	4	4	2	2	2	9	6	15	7.5	9	9	18	9	11	16	27	13.5	3	5	8	4	0.16	0.13	0.16	0.13	0.13	0.13	0.16	0.16	0.16	0.09	0.09	0.09
P31947-1	2810	SFN	14-3-3 protein sigma	SFN,YWHAS	0	0	0	0	0	0	2	2	2	0	0	0	0	0	0	0	0	0	0	0	7	11	18	9	0	0	0	0	0.00	0.00	0.00	0.00	0.00	0.00	0.06	0.06	0.06	0.00	0.00	0.00
P27348	10971	YWHAQ	14-3-3 protein theta	YWHAQ,1C5,HS1,14-3-3	3	2	4	4	2	4	5	5	5	0	0	0	4	4	8	4	11	7	18	9	11	14	25	12.5	0	0	0	0	0.11	0.08	0.16	0.18	0.08	0.18	0.19	0.19	0.19	0.00	0.00	0.00
P63104	7534	YWHAZ	14-3-3 protein zeta/delta	YWHAZ,KCIP-1,MGC111427,MGC126532,MGC138156,14-3-3-zeta	4	4	4	5	5	5	6	5	6	2	0	2	7	7	14	7	13	11	24	12	18	19	37	18.5	3	0	3	1.5	0.21	0.21	0.21	0.26	0.26	0.26	0.31	0.26	0.31	0.09	0.00	0.09
P25398	6206	RPS12	40S ribosomal protein S12	RPS12	0	0	0	0	0	0	0	0	0	0	0	0	0	0	0	0	0	0	0	0	0	0	0	0	0	0	0	0	0.00	0.00	0.00	0.00	0.00	0.00	0.00	0.00	0.00	0.00	0.00	0.00
P62263	6208	RPS14	40S ribosomal protein S14	RPS14,EMTB	0	0	0	0	0	0	0	2	2	0	0	0	0	0	0	0	0	0	0	0	0	3	3	1.5	0	0	0	0	0.00	0.00	0.00	0.00	0.00	0.00	0.00	0.16	0.16	0.00	0.00	0.00
P62269	6222	RPS18	40S ribosomal protein S18	RPS18,KE3,HKE3,KE-3,D6S218E,MGC117351,MGC126835,MGC126837	0	0	0	0	0	0	0	0	0	2	2	2	0	0	0	0	0	0	0	0	0	0	0	0	3	7	10	5	0.00	0.00	0.00	0.00	0.00	0.00	0.00	0.00	0.00	0.12	0.12	0.12
P39019	6223	RPS19	40S ribosomal protein S19	RPS19,DBA	0	0	0	0	0	0	0	0	0	2	2	2	0	0	0	0	0	0	0	0	0	0	0	0	2	3	5	2.5	0.00	0.00	0.00	0.00	0.00	0.00	0.00	0.00	0.00	0.12	0.12	0.12
P62851	6230	RPS25	40S ribosomal protein S25	RPS25	4	3	4	4	3	4	0	2	2	3	4	4	12	6	18	9	16	13	29	14.5	0	6	6	3	23	11	34	17	0.30	0.24	0.30	0.30	0.22	0.30	0.00	0.15	0.15	0.21	0.30	0.30
P62854	6231	RPS26	40S ribosomal protein S26	RPS26,MGC104292	3	3	3	3	0	3	2	2	2	3	3	3	3	5	8	4	5	0	5	2.5	2	3	5	2.5	4	7	11	5.5	0.27	0.27	0.27	0.27	0.00	0.27	0.14	0.14	0.14	0.27	0.27	0.27
P23396	6188	RPS3	40S ribosomal protein S3	RPS3,FLJ26283,FLJ27450,MGC87870	4	3	4	0	0	0	2	0	2	2	0	2	10	4	14	7	0	0	0	0	3	0	3	1.5	5	0	5	2.5	0.19	0.14	0.19	0.00	0.00	0.00	0.09	0.00	0.09	0.09	0.00	0.09
P62861	NA	FAU	40S ribosomal protein S30	NA	0	0	0	2	0	2	0	0	0	0	0	0	0	0	0	0	19	0	19	9.5	0	0	0	0	0	0	0	0	0.00	0.00	0.00	0.19	0.00	0.19	0.00	0.00	0.00	0.00	0.00	0.00
P62701	6191	RPS4X	40S ribosomal protein S4, X isoform	RPS4X,CCG2,SCAR,SCR10,DXS306,FLJ40595	5	2	5	4	0	4	0	0	0	4	5	5	9	5	14	7	7	0	7	3.5	0	0	0	0	7	10	17	8.5	0.16	0.08	0.16	0.13	0.00	0.13	0.00	0.00	0.00	0.16	0.20	0.20
P62081	6201	RPS7	40S ribosomal protein S7	RPS7,DBA8	2	0	2	0	0	0	2	0	2	0	0	0	5	0	5	2.5	0	0	0	0	3	0	3	1.5	0	0	0	0	0.10	0.00	0.10	0.00	0.00	0.00	0.10	0.00	0.10	0.00	0.00	0.00
P08865	3921	RPSA	40S ribosomal protein SA	RPSA,LBP,LRP,p40,67LR,37LRP,LAMBR,LAMR1	0	0	0	0	0	0	0	0	0	2	2	2	0	0	0	0	0	0	0	0	0	0	0	0	2	5	7	3.5	0.00	0.00	0.00	0.00	0.00	0.00	0.00	0.00	0.00	0.10	0.10	0.10
P08195-1	6520	SLC3A2	4F2 cell-surface antigen heavy chain	SLC3A2,CD98HC,NACAE,4T2HC,4F2HC,MDU1,CD98,4F2	0	0	0	2	0	2	2	0	2	0	0	0	0	0	0	0	3	0	3	1.5	2	0	2	1	0	0	0	0	0.00	0.00	0.00	0.03	0.00	0.03	0.03	0.00	0.03	0.00	0.00	0.00
Q13131-1	5562	PRKAA1	5'-AMP-activated protein kinase catalytic subunit alpha-1	PRKAA1,MGC57364,MGC33776,AMPKa1,AMPK	0	0	0	5	5	5	0	0	0	0	0	0	0	0	0	0	18	15	33	16.5	0	0	0	0	0	0	0	0	0.00	0.00	0.00	0.11	0.11	0.11	0.00	0.00	0.00	0.00	0.00	0.00
P54619	5571	PRKAG1	5'-AMP-activated protein kinase subunit gamma-1	PRKAG1,AMPKG,MGC8666	0	0	0	7	6	7	0	0	0	0	0	0	0	0	0	0	9	13	22	11	0	0	0	0	0	0	0	0	0.00	0.00	0.00	0.24	0.18	0.24	0.00	0.00	0.00	0.00	0.00	0.00
P10809	3329	HSPD1	60 kDa heat shock protein, mitochondrial	HSPD1,HLD4,CPN60,GROEL,HSP60,HSP65,SPG13,HuCHA60	7	6	7	7	7	8	11	9	11	5	3	5	10	9	19	9.5	13	11	24	12	36	39	75	37.5	6	4	10	5	0.18	0.16	0.18	0.18	0.17	0.20	0.26	0.18	0.26	0.12	0.06	0.12
P10155-1	6738	TROVE2	60 kDa SS-A/Ro ribonucleoprotein	TROVE2,SSA2,RO60	0	0	0	0	0	0	0	2	2	4	3	5	0	0	0	0	0	0	0	0	0	5	5	2.5	5	4	9	4.5	0.00	0.00	0.00	0.00	0.00	0.00	0.00	0.04	0.04	0.08	0.06	0.11
P35268	6146	RPL22	60S ribosomal protein L22	RPL22,EAP,HBP15,HBP15/L22	2	2	2	0	2	2	0	2	2	0	2	2	11	9	20	10	0	10	10	5	0	3	3	1.5	0	12	12	6	0.20	0.20	0.20	0.00	0.20	0.20	0.00	0.20	0.20	0.00	0.20	0.20
P62750	6147	RPL23A	60S ribosomal protein L23a	RPL23A,MDA20,FLJ27455	2	0	2	0	0	0	0	2	2	0	2	2	2	0	2	1	0	0	0	0	0	3	3	1.5	0	4	4	2	0.15	0.00	0.15	0.00	0.00	0.00	0.00	0.15	0.15	0.00	0.15	0.15
P83881	6173	RPL36A	60S ribosomal protein L36a	RPL36A,L44L,MIG6,RPL44,MGC72020	0	0	0	0	0	0	0	0	0	0	0	0	0	0	0	0	0	0	0	0	0	0	0	0	0	0	0	0	0.00	0.00	0.00	0.00	0.00	0.00	0.00	0.00	0.00	0.00	0.00	0.00
P36639-1	4521	NUDT1	7,8-dihydro-8-oxoguanine triphosphatase	NUDT1,MTH1	0	0	0	3	2	3	0	0	0	0	0	0	0	0	0	0	4	2	6	3	0	0	0	0	0	0	0	0	0.00	0.00	0.00	0.20	0.14	0.20	0.00	0.00	0.00	0.00	0.00	0.00
P11021	3309	HSPA5	78 kDa glucose-regulated protein	HSPA5,BIP,MIF2,GRP78,FLJ26106	8	6	9	9	9	10	9	8	11	5	4	5	16	12	28	14	19	17	36	18	25	21	46	23	7	5	12	6	0.17	0.14	0.19	0.19	0.19	0.22	0.18	0.17	0.23	0.10	0.08	0.10
Q8IZP0-1	10006	ABI1	Abl interactor 1	ABI1,SSH3BP1,SSH3BP,NAP1BP,ABI-1,E3B1	0	0	0	0	0	0	0	0	0	0	0	0	0	0	0	0	0	0	0	0	0	0	0	0	0	0	0	0	0.00	0.00	0.00	0.00	0.00	0.00	0.00	0.00	0.00	0.00	0.00	0.00
P24752	38	ACAT1	Acetyl-CoA acetyltransferase, mitochondrial	ACAT1,T2,MAT,ACAT,THIL	0	0	0	0	0	0	2	2	2	0	0	0	0	0	0	0	0	0	0	0	3	3	6	3	0	0	0	0	0.00	0.00	0.00	0.00	0.00	0.00	0.05	0.05	0.05	0.00	0.00	0.00
P39687	8125	ANP32A	Acidic leucine-rich nuclear phosphoprotein 32 family member A	ANP32A,LANP,MAPM,PP32,PHAP1,PHAPI,I1PP2A,C15orf1,MGC119787,MGC150373	0	0	0	0	0	0	2	3	4	0	0	0	0	0	0	0	0	0	0	0	7	6	13	6.5	0	0	0	0	0.00	0.00	0.00	0.00	0.00	0.00	0.08	0.12	0.15	0.00	0.00	0.00
P68032	70	ACTC1	Actin, alpha cardiac muscle 1	ACTC1,ACTC,ASD5,CMD1R,CMH11	3	2	3	3	3	3	6	7	7	0	0	0	13	7	20	10	7	4	11	5.5	30	41	71	35.5	0	0	0	0	0.10	0.07	0.10	0.10	0.10	0.10	0.18	0.20	0.20	0.00	0.00	0.00
P60709	60	ACTB	Actin, cytoplasmic 1	ACTB,PS1TP5BP1	5	4	5	5	5	5	9	10	10	4	4	4	18	10	28	14	14	10	24	12	63	74	137	68.5	13	9	22	11	0.18	0.15	0.18	0.18	0.18	0.18	0.29	0.31	0.31	0.13	0.13	0.13

AC	ID	Gene_symbol	Descr	Dasatinib score	Bosutinib score	Bafetinib score	Nilotinib score
Q2M2I8	AAK1_HUMAN	AAK1	AP2-associated protein kinase 1	0	49.8325	0	0
Q8IZP0-1	ABI1_HUMAN	ABI1	Abl interactor 1	0.9425	0.45	0	0
P00519-1	ABL1_HUMAN	ABL1	Tyrosine-protein kinase ABL1	68.46	37.125	10.75	0.1725
P42684-1	ABL2_HUMAN	ABL2	Tyrosine-protein kinase ABL2	22.4025	10.1325	5.16	0
P60709	ACTB_HUMAN	ACTB	Actin, cytoplasmic 1	0.525	9.45	0.7875	1.35
P55263-1	ADK_HUMAN	ADK	Adenosine kinase	23.54	0.0525	0	0
O95782-1	AP2A1_HUMAN	AP2A1	AP-2 complex subunit alpha-1	0	5.1975	0	0
Q96CW1-1	AP2M1_HUMAN	AP2M1	AP-2 complex subunit mu	0.105	0.09	0	0
bcrabl	bcrabl	BCR-ABL	BCR-ABL fusion protein	135.975	79.975	24.4375	0.27
P51451	BLK_HUMAN	BLK	Tyrosine-protein kinase Blk	23.1275	7.395	0.405	0
Q9NSY1-1	BMP2K_HUMAN	BMP2K	BMP-2-inducible protein kinase	0	16.64	0	0
Q06187	BTK_HUMAN	BTK	Tyrosine-protein kinase BTK	488.895	393.7375	4.37	11.7075
Q8WUW1-1	BRK1_HUMAN	C3orf10	Probable protein BRICK1	1.5725	0.5625	0	0
Q8NI60-1	ADCK3_HUMAN	CABC1	Chaperone activity of bc1 complex-like, mitochondrial	4.9875	0	0	0
Q13554-3	KCC2B_HUMAN	CAMK2B	Calcium/calmodulin-dependent protein kinase type II subunit beta	0	40.235	0	0.09
Q13557-1	KCC2D_HUMAN	CAMK2D	Calcium/calmodulin-dependent protein kinase type II subunit delta	0	223.02	0	0.5775
Q13555-3	KCC2G_HUMAN	CAMK2G	Calcium/calmodulin-dependent protein kinase type II subunit gamma	0	78.435	0	0.09
Q14004-1	CDK13_HUMAN	CDK13	Cell division protein kinase 13	0.44	0	0	0
Q00535	CDK5_HUMAN	CDK5	Cell division protein kinase 5	0	0	0	0.0525
P50750-1	CDK9_HUMAN	CDK9	Cell division protein kinase 9	0	0	0	1.65
O96017-1	CHK2_HUMAN	CHEK2	Serine/threonine-protein kinase Chk2	0	1.3	0	0
P41240	CSK_HUMAN	CSK	Tyrosine-protein kinase CSK	152.2625	50.43	5.0625	21.3125
P68400	CSK21_HUMAN	CSNK2A1	Casein kinase II subunit alpha	2.325	0.8925	0.025	0
P19784	CSK22_HUMAN	CSNK2A2	Casein kinase II subunit alpha'	0.4275	0.36	0	0
P67870	CSK2B_HUMAN	CSNK2B	Casein kinase II subunit beta	0.225	0.27	0.1125	0
Q96F07-1	CYFP2_HUMAN	CYFIP2	Cytoplasmic FMR1-interacting protein 2	0.54	0.06	0	0
P06733-1	ENOA_HUMAN	ENO1	Alpha-enolase	0	1.485	0	0.2275
P13929-1	ENOB_HUMAN	ENO3	Beta-enolase	0	0.21	0	0.04
P54753	EPHB3_HUMAN	EPHB3	Ephrin type-B receptor 3	7.8	4.44	0	0
P54760	EPHB4_HUMAN	EPHB4	Ephrin type-B receptor 4	38.88	49.9625	0	0
P22830	HEMH_HUMAN	FECH	Ferrochelatase, mitochondrial	0	0	0	3.3
P16591	FER_HUMAN	FER	Tyrosine-protein kinase Fer	0	7.14	0	0
Q8NFF5-1	FAD1_HUMAN	FLAD1	FAD synthase	0	0	18.655	0
P42685	FRK_HUMAN	FRK	Tyrosine-protein kinase FRK	1.3575	0.6675	0	0
P06241-2	FYN_HUMAN	FYN	Tyrosine-protein kinase Fyn	21.75	18.6875	0.24	0
O14976	GAK_HUMAN	GAK	Cyclin-G-associated kinase	8.625	54.1125	0	0
P04406	G3P_HUMAN	GAPDH	Glyceraldehyde-3-phosphate dehydrogenase	0.045	0.42	0	0.99
P14136-1	GFAP_HUMAN	GFAP	Glial fibrillary acidic protein	0.16	0.18	0.27	0.97
Q13588	GRAP_HUMAN	GRAP	GRB2-related adapter protein	4.4	4.2	0.375	0
P62993-1	GRB2_HUMAN	GRB2	Growth factor receptor-bound protein 2	44.01	46.44	19.875	0.09
P08631-1	HCK_HUMAN	HCK	Tyrosine-protein kinase HCK	11.72	16.905	0.405	0.1725
P10412	H14_HUMAN	HIST1H1E	Histone H1.4	0.1125	0.0675	0.77	0.7975
O60814	H2B1K_HUMAN	HIST1H2BK	Histone H2B type 1-K	0	1.575	1.8375	4.64
P08107	HSP71_HUMAN	HSPA1B	Heat shock 70 kDa protein 1A/1B	0.6475	1.785	0.2975	0.6625
P34931	HS71L_HUMAN	HSPA1L	Heat shock 70 kDa protein 1-like	1	1.55	0.8	1.5
P17066	HSP76_HUMAN	HSPA6	Heat shock 70 kDa protein 6	0.6475	1.44	0.2975	0.6625
P04792	HSPB1_HUMAN	HSPB1	Heat shock protein beta-1	0.9775	2.535	0.715	1.0075
Q14164	IKKE_HUMAN	IKBKE	Inhibitor of nuclear factor kappa-B kinase subunit epsilon	0	2.2425	0	0
Q13418	ILK_HUMAN	ILK	Integrin-linked protein kinase	40.32	0.15	0	0
O15357-1	SHIP2_HUMAN	INPPL1	Phosphatidylinositol-3,4,5-trisphosphate 5-phosphatase 2	0.49	0.48	0	0
Q9NWZ3	IRAK4_HUMAN	IRAK4	Interleukin-1 receptor-associated kinase 4	0.0375	3.12	0	0
P06239-1	LCK_HUMAN	LCK	Tyrosine-protein kinase Lck	76.0225	54.795	21.06	0.715
P07195	LDHB_HUMAN	LDHB	L-lactate dehydrogenase B chain	0	1.24	0.03	0
P53671-1	LIMK2_HUMAN	LIMK2	LIM domain kinase 2	0.945	0	0	0
P48059	LIMS1_HUMAN	LIMS1	LIM and senescent cell antigen-like-containing domain protein 1	28.8	0.04	0	0
Q7Z4I7-1	LIMS2_HUMAN	LIMS2	LIM and senescent cell antigen-like-containing domain protein 2	5.875	0	0	0
P07948-2	LYN_HUMAN	LYN	Tyrosine-protein kinase Lyn	212.3875	182.8575	25.0575	3.7525
Q02750	MP2K1_HUMAN	MAP2K1	Dual specificity mitogen-activated protein kinase kinase 1	0	59.5725	0	0
P36507	MP2K2_HUMAN	MAP2K2	Dual specificity mitogen-activated protein kinase kinase 2	0	78.21	0	0
Q13163-1	MP2K5_HUMAN	MAP2K5	Dual specificity mitogen-activated protein kinase kinase 5	0	0.9425	0	0
Q13233	M3K1_HUMAN	MAP3K1	Mitogen-activated protein kinase kinase kinase 1	1.38	6.555	0	0
Q9Y2U5	M3K2_HUMAN	MAP3K2	Mitogen-activated protein kinase kinase kinase 2	0.7	19.955	0	0
Q99759-1	M3K3_HUMAN	MAP3K3	Mitogen-activated protein kinase kinase kinase 3	0.45	6.76	0	0
Q92918	M4K1_HUMAN	MAP4K1	Mitogen-activated protein kinase kinase kinase kinase 1	0	4.68	0	0
Q12851	M4K2_HUMAN	MAP4K2	Mitogen-activated protein kinase kinase kinase kinase 2	0	24.5	0	0
Q8IVH8-1	M4K3_HUMAN	MAP4K3	Mitogen-activated protein kinase kinase kinase kinase 3	0	0.1	0	0
O95819-1	M4K4_HUMAN	MAP4K4	Mitogen-activated protein kinase kinase kinase kinase 4	0	0.35	0	0
Q9Y4K4	M4K5_HUMAN	MAP4K5	Mitogen-activated protein kinase kinase kinase kinase 5	4	31.7625	0	0
P53779-1	MK10_HUMAN	MAPK10	Mitogen-activated protein kinase 10	0	0	3.29	13.5
Q16539-1	MK14_HUMAN	MAPK14	Mitogen-activated protein kinase 14	37.56	0	78.6675	76.8
P45983-3	MK08_HUMAN	MAPK8	Mitogen-activated protein kinase 8	0	0	5.58	55.3
P45984-1	MK09_HUMAN	MAPK9	Mitogen-activated protein kinase 9	0	0	13.98	45.6875
P49137-1	MAPK2_HUMAN	MAPKAPK2	MAP kinase-activated protein kinase 2	0	0	0.06	0
Q16644	MAPK3_HUMAN	MAPKAPK3	MAP kinase-activated protein kinase 3	0	0	3.3925	0.275
Q9NYL2-2	MLTK_HUMAN	MLTK	Mitogen-activated protein kinase kinase kinase MLT	18.81	20.8	36.9075	21.6
Q9P289	MST4_HUMAN	MST4	Serine/threonine-protein kinase MST4	0	2.55	0	0
P60660-1	MYL6_HUMAN	MYL6	Myosin light polypeptide 6	0	0	0.45	0.15
P16083	NQO2_HUMAN	NQO2	Ribosyldihydronicotinamide dehydrogenase [quinone]	0	0	1.0625	0
Q9UKK9	NUDT5_HUMAN	NUDT5	ADP-sugar pyrophosphatase	3.2625	0	0	0
Q9NWQ8	PAG1_HUMAN	PAG1	Phosphoprotein associated with glycosphingolipid-enriched microdomains 1	2.405	0.22	0	0
Q9HBI0-1	PARVG_HUMAN	PARVG	Gamma-parvin	51.92	1.3175	0	0
Q9BUL8	PDC10_HUMAN	PDCD10	Programmed cell death protein 10	0	0.6875	0	0
P09619	PGFRB_HUMAN	PDGFRB	Beta-type platelet-derived growth factor receptor	8.04	0	0	0
Q99640	PMYT1_HUMAN	PKMYT1	Membrane-associated tyrosine- and threonine-specific cdc2-inhibitory kinase	4.03	5.58	0	0
Q06203	PUR1_HUMAN	PPAT	Amidophosphoribosyltransferase	16.96	0	0	0
P23284	PPIB_HUMAN	PPIB	Peptidyl-prolyl cis-trans isomerase B	0.045	0.405	1.35	0.69
Q13131-1	AAPK1_HUMAN	PRKAA1	5'-AMP-activated protein kinase catalytic subunit alpha-1	0	4.23	0	0
Q05397-1	FAK1_HUMAN	PTK2	Focal adhesion kinase 1	0	8.4575	0	0
Q14289-2	FAK2_HUMAN	PTK2B	Protein-tyrosine kinase 2-beta	0	6.93	16.125	1.82
O43353-1	RIPK2_HUMAN	RIPK2	Receptor-interacting serine/threonine-protein kinase 2	29.785	0	0	0
Q15404	RSU1_HUMAN	RSU1	Ras suppressor protein 1	54.52	0.5625	0	0
P31947-1	1433S_HUMAN	SFN	14-3-3 protein sigma	1.7775	1.62	0.495	0.03
Q86YV5	SG223_HUMAN	SGK223	Tyrosine-protein kinase SgK223	1.7875	0.2975	0	0
Q9H0K1	SIK2_HUMAN	SIK2	Serine/threonine-protein kinase SIK2	1.7625	0.55	0	0
Q9H2G2-1	SLK_HUMAN	SLK	STE20-like serine/threonine-protein kinase	0	5.58	0	0
P12931-1	SRC_HUMAN	SRC	Proto-oncogene tyrosine-protein kinase Src	9.4875	5.985	0	0
O94804	STK10_HUMAN	STK10	Serine/threonine-protein kinase 10	0	30.225	0	0
Q7RTN6-1	STRAA_HUMAN	STRADA	STE20-related kinase adapter protein alpha	0.03	0	0	0
P53999	TCP4_HUMAN	SUB1	Activated RNA polymerase II transcriptional coactivator p15	6.65	3.01	5.67	7.21
Q9UHD2	TBK1_HUMAN	TBK1	Serine/threonine-protein kinase TBK1	0	6.045	0	0
P42680	TEC_HUMAN	TEC	Tyrosine-protein kinase Tec	1.47	0	0	0
Q15569	TESK1_HUMAN	TESK1	Dual specificity testis-specific protein kinase 1	0.42	0	0	0
Q71U36	TBA1A_HUMAN	TUBA1A	Tubulin alpha-1A chain	19.3875	29.07	16.74	21.6975
P68363	TBA1B_HUMAN	TUBA1B	Tubulin alpha-1B chain	21.96	30.4	16.92	24.66
Q9BQE3	TBA1C_HUMAN	TUBA1C	Tubulin alpha-1C chain	19.47	29.26	16.74	23.85
P49411	EFTU_HUMAN	TUFM	Elongation factor Tu, mitochondrial	0	0.675	0	0
P29597	TYK2_HUMAN	TYK2	Non-receptor tyrosine-protein kinase TYK2	0.02	0	0	0
Q8TF42	UBS3B_HUMAN	UBASH3B	Ubiquitin-associated and SH3 domain-containing protein B	0.55	0.1575	0	0
Q6PHR2-1	ULK3_HUMAN	ULK3	Serine/threonine-protein kinase ULK3	0	7.9825	0	0
Q13432-1	U119A_HUMAN	UNC119	Protein unc-119 homolog A	1.5	1.92	0.7	0
P07947	YES_HUMAN	YES1	Proto-oncogene tyrosine-protein kinase Yes	22.815	10.3125	0.24	0
P31946-1	1433B_HUMAN	YWHAB	14-3-3 protein beta/alpha	8.4975	10.01	1.75	0.035
P62258	1433E_HUMAN	YWHAE	14-3-3 protein epsilon	10.44	11.7	5.28	0.4375
P61981	1433G_HUMAN	YWHAG	14-3-3 protein gamma	18.815	16.56	5.3475	1.2
Q04917	1433F_HUMAN	YWHAH	14-3-3 protein eta	4.5125	4.4175	2.2875	0.035
P27348	1433T_HUMAN	YWHAQ	14-3-3 protein theta	9.9	9.72	3.78	0.1925
P63104	1433Z_HUMAN	YWHAZ	14-3-3 protein zeta/delta	26.04	29.37	6.545	2.21

AC	ID	Gene_symbol	Descr	Dasatinib score	Bosutinib score	Bafetinib score	Nilotinib score
Q2M2I8	AAK1_HUMAN	AAK1	AP2-associated protein kinase 1	0	42.352	0	0
P42684-1	ABL2_HUMAN	ABL2	Tyrosine-protein kinase ABL2	4.424	1.0242	0.11	0
P60709	ACTB_HUMAN	ACTB	Actin, cytoplasmic 1	5.1875	3.024	3.08	3.3
P68032	ACTC_HUMAN	ACTC1	Actin, alpha cardiac muscle 1	2.655	1.3325	1.182	1.54
P55263-1	ADK_HUMAN	ADK	Adenosine kinase	12.69	1.405	0	0
O95782-1	AP2A1_HUMAN	AP2A1	AP-2 complex subunit alpha-1	0	4.0964	0	0
O94973-1	AP2A2_HUMAN	AP2A2	AP-2 complex subunit alpha-2	0	0.46	0	0
P63010-1	AP2B1_HUMAN	AP2B1	AP-2 complex subunit beta	0	0.9408	0	0
Q96CW1-1	AP2M1_HUMAN	AP2M1	AP-2 complex subunit mu	0	0.3094	0	0
P53680-1	AP2S1_HUMAN	AP2S1	AP-2 complex subunit sigma	0	0.3406	0	0
bcrabl	bcrabl	BCR-ABL	BCR-ABL fusion protein	2.6	1.262	0.025	0
P51451	BLK_HUMAN	BLK	Tyrosine-protein kinase Blk	6.215	0.63	0	0
Q9NSY1-1	BMP2K_HUMAN	BMP2K	BMP-2-inducible protein kinase	0	9.6912	0	0
Q06187	BTK_HUMAN	BTK	Tyrosine-protein kinase BTK	256.725	151.335	0	0.15
Q8NI60-1	ADCK3_HUMAN	CABC1	Chaperone activity of bc1 complex-like, mitochondrial	0.1908	0	0	0
Q13555-1	KCC2G_HUMAN	CAMK2G	Calcium/calmodulin-dependent protein kinase type II subunit gamma	0	8.025	0	0
Q14004-1	CDK13_HUMAN	CDK13	Cell division protein kinase 13	0.0262	0	0	0
Q07002-1	CDK18_HUMAN	CDK18	Cell division protein kinase 18	0.119	0	0	0
P50750-1	CDK9_HUMAN	CDK9	Cell division protein kinase 9	0	0	0	0.0434
O96017-1	CHK2_HUMAN	CHEK2	Serine/threonine-protein kinase Chk2	0	0.6048	0	0
Q16630-1	CPSF6_HUMAN	CPSF6	Cleavage and polyadenylation specificity factor subunit 6	1.032	0.7828	2.2	1.76
Q8N684-1	CPSF7_HUMAN	CPSF7	Cleavage and polyadenylation specificity factor subunit 7	0.35	0.05	0.04	0.212
P41240	CSK_HUMAN	CSK	Tyrosine-protein kinase CSK	21.115	5.1852	0	0.843
P27707	DCK_HUMAN	DCK	Deoxycytidine kinase	0	2.925	0	0
P59665	DEF1_HUMAN	DEFA1B	Neutrophil defensin 1	0	0.38	0	0
P54760	EPHB4_HUMAN	EPHB4	Ephrin type-B receptor 4	38.34	27.114	0	0
P22830	HEMH_HUMAN	FECH	Ferrochelatase, mitochondrial	2.0688	2.2475	0	8.3232
P16591	FER_HUMAN	FER	Tyrosine-protein kinase Fer	0	0.455	0	0
P07332	FES_HUMAN	FES	Tyrosine-protein kinase Fes/Fps	0	0.0186	0	0
Q8NFF5-1	FAD1_HUMAN	FLAD1	FAD synthase	0	0	0.4192	0
P36888	FLT3_HUMAN	FLT3	FL cytokine receptor	0.0424	0	0	0
P42685	FRK_HUMAN	FRK	Tyrosine-protein kinase FRK	0.225	0.0486	0	0
P06241-2	FYN_HUMAN	FYN	Tyrosine-protein kinase Fyn	10.295	5.076	0	0
O14976	GAK_HUMAN	GAK	Cyclin-G-associated kinase	2.38	15.0016	0	0
P14136-1	GFAP_HUMAN	GFAP	Glial fibrillary acidic protein	0.0648	0.4352	0.1448	0.12
P62993-1	GRB2_HUMAN	GRB2	Growth factor receptor-bound protein 2	0.98	0.9802	0	0
P08631-1	HCK_HUMAN	HCK	Tyrosine-protein kinase HCK	1.98	2.4408	0.1386	0
P08107	HSP71_HUMAN	HSPA1B	Heat shock 70 kDa protein 1A/1B	0.225	1.4625	0.194	0.5625
P34931	HS71L_HUMAN	HSPA1L	Heat shock 70 kDa protein 1-like	0.6096	1.238	0.52	0.495
P17066	HSP76_HUMAN	HSPA6	Heat shock 70 kDa protein 6	0.225	0.9792	0.194	0.2925
Q14164	IKKE_HUMAN	IKBKE	Inhibitor of nuclear factor kappa-B kinase subunit epsilon	0	0.0375	0	0
Q13418	ILK_HUMAN	ILK	Integrin-linked protein kinase	23.679	0	0	0
Q9Y616	IRAK3_HUMAN	IRAK3	Interleukin-1 receptor-associated kinase 3	0.0434	0	0	0
Q9NWZ3	IRAK4_HUMAN	IRAK4	Interleukin-1 receptor-associated kinase 4	0.18	2.3184	0	0
P35968	VGFR2_HUMAN	KDR	Vascular endothelial growth factor receptor 2	0.0038	0	0	0
P06239-1	LCK_HUMAN	LCK	Tyrosine-protein kinase Lck	18.1368	13.28	0.72	0
P07195	LDHB_HUMAN	LDHB	L-lactate dehydrogenase B chain	0	0.1696	0	0
Q32P28-1	P3H1_HUMAN	LEPRE1	Prolyl 3-hydroxylase 1	0.3504	0.06	0.5742	0.3696
P53671-1	LIMK2_HUMAN	LIMK2	LIM domain kinase 2	0.0228	0	0	0
P48059	LIMS1_HUMAN	LIMS1	LIM and senescent cell antigen-like-containing domain protein 1	7.7128	0	0	0
Q9Y383-1	LC7L2_HUMAN	LUC7L2	Putative RNA-binding protein Luc7-like 2	1.05	0.9792	1	1.35
P07948-2	LYN_HUMAN	LYN	Tyrosine-protein kinase Lyn	107.328	83.3625	5.8146	0.0152
Q02750	MP2K1_HUMAN	MAP2K1	Dual specificity mitogen-activated protein kinase kinase 1	0	27.935	0	0
P36507	MP2K2_HUMAN	MAP2K2	Dual specificity mitogen-activated protein kinase kinase 2	0	38.6952	0	0
Q13163-1	MP2K5_HUMAN	MAP2K5	Dual specificity mitogen-activated protein kinase kinase 5	0	0.0228	0	0
Q13233	M3K1_HUMAN	MAP3K1	Mitogen-activated protein kinase kinase kinase 1	0	0.131	0	0
Q9Y2U5	M3K2_HUMAN	MAP3K2	Mitogen-activated protein kinase kinase kinase 2	0.21	8.8088	0	0
Q99759-1	M3K3_HUMAN	MAP3K3	Mitogen-activated protein kinase kinase kinase 3	0.0636	2.8125	0	0
Q92918	M4K1_HUMAN	MAP4K1	Mitogen-activated protein kinase kinase kinase kinase 1	0	0.9256	0	0
Q12851	M4K2_HUMAN	MAP4K2	Mitogen-activated protein kinase kinase kinase kinase 2	0	1.5808	0	0
O95819-1	M4K4_HUMAN	MAP4K4	Mitogen-activated protein kinase kinase kinase kinase 4	0	0.0375	0	0
Q9Y4K4	M4K5_HUMAN	MAP4K5	Mitogen-activated protein kinase kinase kinase kinase 5	2.1375	9.035	0	0
Q16539-1	MK14_HUMAN	MAPK14	Mitogen-activated protein kinase 14	33.8432	0.1504	29.9934	19.3704
P45983-3	MK08_HUMAN	MAPK8	Mitogen-activated protein kinase 8	0	0	0.2	13.0708
P45984-1	MK09_HUMAN	MAPK9	Mitogen-activated protein kinase 9	0	0	2.3544	11.767
Q16644	MAPK3_HUMAN	MAPKAPK3	MAP kinase-activated protein kinase 3	0	0	0.0125	0
Q9NYL2-2	MLTK_HUMAN	MLTK	Mitogen-activated protein kinase kinase kinase MLT	13.6648	8.8776	12.5425	5.25
Q9P289	MST4_HUMAN	MST4	Serine/threonine-protein kinase MST4	0	0.9775	0	0
P35579-1	MYH9_HUMAN	MYH9	Myosin-9	2.2232	1.3325	3.5784	1.9125
O14950	ML12B_HUMAN	MYL12B	Myosin regulatory light chain 12B	0.48	0.78	1.38	0.57
Q6ZSR9	YJ005_HUMAN	NA	Uncharacterized protein FLJ45252	0	15.77	0	0
P16083	NQO2_HUMAN	NQO2	Ribosyldihydronicotinamide dehydrogenase [quinone]	0	0	4.7275	2.5978
O43809	CPSF5_HUMAN	NUDT21	Cleavage and polyadenylation specificity factor subunit 5	44.928	47.203	55.497	46.41
Q9UKK9	NUDT5_HUMAN	NUDT5	ADP-sugar pyrophosphatase	7.0744	0	0	0
P13674-1	P4HA1_HUMAN	P4HA1	Prolyl 4-hydroxylase subunit alpha-1	1.55	1.52	3.915	2.6325
Q9HBI0-1	PARVG_HUMAN	PARVG	Gamma-parvin	23.4866	1.5912	0	0
Q9BUL8	PDC10_HUMAN	PDCD10	Programmed cell death protein 10	0	0.855	0	0
P09619	PGFRB_HUMAN	PDGFRB	Beta-type platelet-derived growth factor receptor	19.845	0	0	0
Q99623	PHB2_HUMAN	PHB2	Prohibitin-2	4.0968	16.7024	5.1675	5.679
Q99640	PMYT1_HUMAN	PKMYT1	Membrane-associated tyrosine- and threonine-specific cdc2-inhibitory kinase	0.1932	1.6675	0	0
A5A3E0	POTEF_HUMAN	POTEF	POTE ankyrin domain family member F	0.51	0.3048	0.26	0.2048
Q06203	PUR1_HUMAN	PPAT	Amidophosphoribosyltransferase	16.314	0	0	0
Q13131-1	AAPK1_HUMAN	PRKAA1	5'-AMP-activated protein kinase catalytic subunit alpha-1	0	2.0688	0	0
Q05397-1	FAK1_HUMAN	PTK2	Focal adhesion kinase 1	0	0.36	0	0
Q14289-2	FAK2_HUMAN	PTK2B	Protein-tyrosine kinase 2-beta	0	5.265	4.7725	0.069
Q96D71-1	REPS1_HUMAN	REPS1	RalBP1-associated Eps domain-containing protein 1	0	0.4268	0	0
O43353-1	RIPK2_HUMAN	RIPK2	Receptor-interacting serine/threonine-protein kinase 2	5.8125	0	0	0
Q15404	RSU1_HUMAN	RSU1	Ras suppressor protein 1	30.535	0.2484	0	0
P31947-1	1433S_HUMAN	SFN	14-3-3 protein sigma	0.3708	0.6975	0.0528	0
Q9H0K1	SIK2_HUMAN	SIK2	Serine/threonine-protein kinase SIK2	0.0372	0.005	0	0
Q9Y2K2-1	SIK3_HUMAN	SIK3	Serine/threonine-protein kinase SIK3	0.106	0	0	0
Q9H2G2-1	SLK_HUMAN	SLK	STE20-like serine/threonine-protein kinase	0	2.3856	0	0
P12931-1	SRC_HUMAN	SRC	Proto-oncogene tyrosine-protein kinase Src	4.68	2.3028	0	0
O94804	STK10_HUMAN	STK10	Serine/threonine-protein kinase 10	0	10.6375	0	0
Q9Y6E0-1	STK24_HUMAN	STK24	Serine/threonine-protein kinase 24	0	0.26	0	0
Q9UHD2	TBK1_HUMAN	TBK1	Serine/threonine-protein kinase TBK1	0	3.5088	0	0
P42680	TEC_HUMAN	TEC	Tyrosine-protein kinase Tec	8.7348	0	0	0
Q96S53-1	TESK2_HUMAN	TESK2	Dual specificity testis-specific protein kinase 2	0.0828	0	0	0
P49411	EFTU_HUMAN	TUFM	Elongation factor Tu, mitochondrial	0	2.8875	0	0
P26368-1	U2AF2_HUMAN	U2AF2	Splicing factor U2AF 65 kDa subunit	1.36	1.5392	2.8864	2.576
Q6PHR2-1	ULK3_HUMAN	ULK3	Serine/threonine-protein kinase ULK3	0	1.155	0	0
Q13432-1	U119A_HUMAN	UNC119	Protein unc-119 homolog A	0.48	0.64	0	0
P07947	YES_HUMAN	YES1	Proto-oncogene tyrosine-protein kinase Yes	11.1302	4.5684	0	0
P31946-1	1433B_HUMAN	YWHAB	14-3-3 protein beta/alpha	1.05	2.0688	0.0616	0
P62258	1433E_HUMAN	YWHAE	14-3-3 protein epsilon	3.9928	7.6518	0.45	0.0875
P61981	1433G_HUMAN	YWHAG	14-3-3 protein gamma	3.4	5.3992	0.5792	0.1242
Q04917	1433F_HUMAN	YWHAH	14-3-3 protein eta	1.032	1.725	0.0616	0
P27348	1433T_HUMAN	YWHAQ	14-3-3 protein theta	2.2736	3.6828	0.3196	0.06
P63104	1433Z_HUMAN	YWHAZ	14-3-3 protein zeta/delta	4.165	9.5786	0.7875	0.315

Cell Line	IKZF1	CDKN2A	PAX5	C20orf94	RB1	MEF2C	EBF1	BTG1	DLEU2	FHIT	ETV6
BV-173	-5.28	-3.0816	0.1053	-0.047	0.0765	0.012	0.2693	0.0036	0.0765	0.1211	0.0182
Z-119	N/A	N/A	N/A	N/A	N/A	N/A	N/A	N/A	N/A	N/A	N/A
SUP-B15	-0.8345	-2.2783	-0.8556	-4.0348	0.0687	-0.0184	0.0457	-0.9328	0.0687	0.0612	0.0158

AC	ID	Gene_symbol	Descr	IKZF1	CDKN2A	PAX5	C20orf94	RB1	MEF2C	EBF1	BTG1	FHIT	ETV6	BCR-ABL
Q2M2I8	AAK1_HUMAN	AAK1	AP2-associated protein kinase 1	2	4	4	4	4	4	4	4	4	3	2
Q8IZP0	ABI1_HUMAN	ABI1	Abl interactor 1	3	2	3	3	2	3	4	4	3	3	3
P00519	ABL1_HUMAN	ABL1	Tyrosine-protein kinase ABL1	3	2	3	3	1	2	3	3	2	2	2
P42684	ABL2_HUMAN	ABL2	Tyrosine-protein kinase ABL2	3	3	3	3	2	3	2	4	2	2	2
P60709	ACTB_HUMAN	ACTB	Actin, cytoplasmic 1	3	1	2	3	2	3	3	3	3	2	3
P68032	ACTC_HUMAN	ACTC1	Actin, alpha cardiac muscle 1	3	3	4	2	3	3	4	3	3	2	2
P55263	ADK_HUMAN	ADK	Adenosine kinase	NA	NA	NA	NA	NA	NA	NA	NA	NA	NA	NA
O95782	AP2A1_HUMAN	AP2A1	AP-2 complex subunit alpha-1	3	3	3	3	2	3	4	3	3	2	1
O94973	AP2A2_HUMAN	AP2A2	AP-2 complex subunit alpha-2	2	3	3	4	3	3	3	3	3	3	2
P63010	AP2B1_HUMAN	AP2B1	AP-2 complex subunit beta	2	3	3	3	3	3	4	3	3	2	1
Q96CW1	AP2M1_HUMAN	AP2M1	AP-2 complex subunit mu	1	3	3	3	3	3	3	3	3	2	1
P53680	AP2S1_HUMAN	AP2S1	AP-2 complex subunit sigma	3	3	4	4	3	3	4	4	3	2	2
bcrabl	bcrabl	BCR-ABL	BCR-ABL fusion protein	2	3	3	4	2	3	4	4	3	2	0
P51451	BLK_HUMAN	BLK	Tyrosine-protein kinase Blk	3	3	3	4	2	3	3	4	3	3	2
Q9NSY1	BMP2K_HUMAN	BMP2K	BMP-2-inducible protein kinase	3	4	4	4	3	4	4	5	4	3	1
Q06187	BTK_HUMAN	BTK	Tyrosine-protein kinase BTK	3	3	3	4	2	3	2	3	3	2	2
Q8WUW1	BRK1_HUMAN	C3orf10	Probable protein BRICK1	NA	NA	NA	NA	NA	NA	NA	NA	NA	NA	NA
Q8NI60	ADCK3_HUMAN	CABC1	Chaperone activity of bc1 complex-like, mitochondrial	4	4	4	3	4	4	4	4	3	4	4
Q13554	KCC2B_HUMAN	CAMK2B	Calcium/calmodulin-dependent protein kinase type II subunit beta	4	2	4	3	3	3	4	4	3	3	3
Q13557	KCC2D_HUMAN	CAMK2D	Calcium/calmodulin-dependent protein kinase type II subunit delta	3	3	4	3	3	3	3	3	3	2	2
Q13555	KCC2G_HUMAN	CAMK2G	Calcium/calmodulin-dependent protein kinase type II subunit gamma	3	2	3	3	3	2	3	4	3	3	3
Q14004	CDK13_HUMAN	CDK13	Cell division protein kinase 13	3	3	4	3	3	4	4	3	3	3	3
Q07002	CDK18_HUMAN	CDK18	Cell division protein kinase 18	3	3	4	4	3	3	4	4	3	3	3
Q00535	CDK5_HUMAN	CDK5	Cell division protein kinase 5	2	2	3	3	2	2	3	3	3	3	3
P50750	CDK9_HUMAN	CDK9	Cell division protein kinase 9	2	2	2	2	1	3	3	3	2	2	3
O96017	CHK2_HUMAN	CHEK2	Serine/threonine-protein kinase Chk2	3	3	3	2	1	3	3	4	3	3	3
Q16630	CPSF6_HUMAN	CPSF6	Cleavage and polyadenylation specificity factor subunit 6	3	2	3	3	3	3	3	3	3	3	3
Q8N684	CPSF7_HUMAN	CPSF7	Cleavage and polyadenylation specificity factor subunit 7	3	3	4	4	2	4	3	3	3	3	3
P41240	CSK_HUMAN	CSK	Tyrosine-protein kinase CSK	3	3	2	3	2	3	2	3	2	3	2
P68400	CSK21_HUMAN	CSNK2A1	Casein kinase II subunit alpha	2	2	3	2	2	2	2	3	2	3	3
P19784	CSK22_HUMAN	CSNK2A2	Casein kinase II subunit alpha'	2	2	3	3	2	3	2	3	2	3	3
P67870	CSK2B_HUMAN	CSNK2B	Casein kinase II subunit beta	3	2	3	2	2	3	3	3	3	3	3
Q96F07	CYFP2_HUMAN	CYFIP2	Cytoplasmic FMR1-interacting protein 2	3	3	3	3	3	3	3	4	3	3	3
P27707	DCK_HUMAN	DCK	Deoxycytidine kinase	3	3	4	4	3	4	4	4	3	3	4
P59665	DEF1_HUMAN	DEFA1B	Neutrophil defensin 1	4	3	4	4	3	4	4	4	4	3	4
P06733	ENOA_HUMAN	ENO1	Alpha-enolase	3	3	3	3	3	3	3	4	3	2	2
P13929	ENOB_HUMAN	ENO3	Beta-enolase	5	5	4	5	4	5	5	5	5	4	5
P54753	EPHB3_HUMAN	EPHB3	Ephrin type-B receptor 3	3	3	4	4	3	3	4	4	3	3	2
P54760	EPHB4_HUMAN	EPHB4	Ephrin type-B receptor 4	3	3	3	4	3	4	4	4	3	3	3
P22830	HEMH_HUMAN	FECH	Ferrochelatase, mitochondrial	4	3	4	3	3	4	4	5	4	4	3
P16591	FER_HUMAN	FER	Tyrosine-protein kinase Fer	3	3	3	3	2	3	3	4	2	3	3
P07332	FES_HUMAN	FES	Tyrosine-protein kinase Fes/Fps	3	2	3	3	2	3	3	3	3	2	2
Q8NFF5	FAD1_HUMAN	FLAD1	FAD synthase	3	4	4	4	3	4	4	5	3	3	4
P36888	FLT3_HUMAN	FLT3	FL cytokine receptor	3	2	3	3	2	3	3	3	3	2	2
P42685	FRK_HUMAN	FRK	Tyrosine-protein kinase FRK	3	3	3	4	1	3	3	4	4	3	3
P06241	FYN_HUMAN	FYN	Tyrosine-protein kinase Fyn	3	2	3	3	2	2	3	3	2	2	2
O14976	GAK_HUMAN	GAK	Cyclin-G-associated kinase	2	3	2	4	2	3	3	3	3	3	2
P04406	G3P_HUMAN	GAPDH	Glyceraldehyde-3-phosphate dehydrogenase	3	1	2	3	2	2	2	3	3	2	3
P14136	GFAP_HUMAN	GFAP	Glial fibrillary acidic protein	3	2	3	3	2	3	3	3	2	3	3
Q13588	GRAP_HUMAN	GRAP	GRB2-related adapter protein	NA	NA	NA	NA	NA	NA	NA	NA	NA	NA	NA
P62993	GRB2_HUMAN	GRB2	Growth factor receptor-bound protein 2	2	2	3	3	2	2	3	3	2	1	1
P08631	HCK_HUMAN	HCK	Tyrosine-protein kinase HCK	3	3	3	3	2	3	3	3	3	2	2
P10412	H14_HUMAN	HIST1H1E	Histone H1.4	3	3	3	4	2	3	3	4	3	2	3
O60814	H2B1K_HUMAN	HIST1H2BK	Histone H2B type 1-K	4	4	4	4	3	4	4	4	4	4	5
P08107	HSP71_HUMAN	HSPA1B	Heat shock 70 kDa protein 1A/1B	3	2	2	3	2	3	3	3	2	3	3
P34931	HS71L_HUMAN	HSPA1L	Heat shock 70 kDa protein 1-like	3	2	3	3	3	3	3	3	3	2	2
P17066	HSP76_HUMAN	HSPA6	Heat shock 70 kDa protein 6	3	3	3	3	2	3	3	3	3	3	3
P04792	HSPB1_HUMAN	HSPB1	Heat shock protein beta-1	2	2	2	2	2	3	3	3	3	2	2
Q14164	IKKE_HUMAN	IKBKE	Inhibitor of nuclear factor kappa-B kinase subunit epsilon	2	1	3	2	2	3	3	3	2	2	2
Q13418	ILK_HUMAN	ILK	Integrin-linked protein kinase	3	3	3	3	2	3	3	3	2	3	3
O15357	SHIP2_HUMAN	INPPL1	Phosphatidylinositol-3,4,5-trisphosphate 5-phosphatase 2	3	3	3	4	2	3	3	4	2	2	1
Q9Y616	IRAK3_HUMAN	IRAK3	Interleukin-1 receptor-associated kinase 3	3	3	3	3	3	4	3	3	3	3	3
Q9NWZ3	IRAK4_HUMAN	IRAK4	Interleukin-1 receptor-associated kinase 4	3	3	3	3	3	3	3	3	3	2	3
P35968	VGFR2_HUMAN	KDR	Vascular endothelial growth factor receptor 2	3	3	3	3	2	3	3	4	2	2	2
P06239	LCK_HUMAN	LCK	Tyrosine-protein kinase Lck	3	2	3	3	2	3	3	3	3	2	2
P07195	LDHB_HUMAN	LDHB	L-lactate dehydrogenase B chain	3	3	3	3	3	3	3	4	2	3	3
Q32P28	P3H1_HUMAN	LEPRE1	Prolyl 3-hydroxylase 1	4	3	4	4	3	4	4	4	4	3	4
P53671	LIMK2_HUMAN	LIMK2	LIM domain kinase 2	4	3	4	4	3	3	4	4	3	3	3
P48059	LIMS1_HUMAN	LIMS1	LIM and senescent cell antigen-like-containing domain protein 1	2	3	4	4	2	4	4	4	3	3	3
Q7Z4I7	LIMS2_HUMAN	LIMS2	LIM and senescent cell antigen-like-containing domain protein 2	4	4	4	4	3	4	4	4	3	4	4
Q9Y383	LC7L2_HUMAN	LUC7L2	Putative RNA-binding protein Luc7-like 2	3	3	3	3	3	3	3	3	3	2	2
P07948	LYN_HUMAN	LYN	Tyrosine-protein kinase Lyn	3	2	3	3	2	3	3	3	2	2	2
Q02750	MP2K1_HUMAN	MAP2K1	Dual specificity mitogen-activated protein kinase kinase 1	3	2	3	3	2	2	2	3	3	3	3
P36507	MP2K2_HUMAN	MAP2K2	Dual specificity mitogen-activated protein kinase kinase 2	3	2	3	3	2	3	3	4	3	3	3
Q13163	MP2K5_HUMAN	MAP2K5	Dual specificity mitogen-activated protein kinase kinase 5	3	3	3	3	3	2	3	4	3	2	2
Q13233	M3K1_HUMAN	MAP3K1	Mitogen-activated protein kinase kinase kinase 1	2	2	3	3	2	3	3	3	2	2	2
Q9Y2U5	M3K2_HUMAN	MAP3K2	Mitogen-activated protein kinase kinase kinase 2	3	2	3	3	3	3	3	3	3	3	3
Q99759	M3K3_HUMAN	MAP3K3	Mitogen-activated protein kinase kinase kinase 3	3	2	3	3	2	3	3	3	3	2	3
Q92918	M4K1_HUMAN	MAP4K1	Mitogen-activated protein kinase kinase kinase kinase 1	3	3	3	4	2	3	3	4	2	2	2
Q12851	M4K2_HUMAN	MAP4K2	Mitogen-activated protein kinase kinase kinase kinase 2	3	3	4	4	3	4	4	4	3	3	3
Q8IVH8	M4K3_HUMAN	MAP4K3	Mitogen-activated protein kinase kinase kinase kinase 3	3	3	4	4	3	3	4	4	3	2	2
O95819	M4K4_HUMAN	MAP4K4	Mitogen-activated protein kinase kinase kinase kinase 4	3	3	3	4	2	3	3	4	3	3	3
Q9Y4K4	M4K5_HUMAN	MAP4K5	Mitogen-activated protein kinase kinase kinase kinase 5	3	3	4	4	2	3	4	4	3	2	2
P53779	MK10_HUMAN	MAPK10	Mitogen-activated protein kinase 10	3	1	3	3	2	3	3	3	3	3	3
Q16539	MK14_HUMAN	MAPK14	Mitogen-activated protein kinase 14	3	2	3	3	2	3	3	3	2	3	2
P45983	MK08_HUMAN	MAPK8	Mitogen-activated protein kinase 8	3	1	3	3	2	3	3	3	2	3	2
P45984	MK09_HUMAN	MAPK9	Mitogen-activated protein kinase 9	3	2	3	3	2	3	3	3	2	2	2
P49137	MAPK2_HUMAN	MAPKAPK2	MAP kinase-activated protein kinase 2	3	3	3	3	3	3	3	4	3	3	2
Q16644	MAPK3_HUMAN	MAPKAPK3	MAP kinase-activated protein kinase 3	3	2	3	3	3	3	3	4	3	2	3
Q9NYL2	MLTK_HUMAN	MLTK	Mitogen-activated protein kinase kinase kinase MLT	3	3	4	3	3	3	4	4	3	3	3
Q9P289	MST4_HUMAN	MST4	Serine/threonine-protein kinase MST4	4	3	3	4	2	3	3	4	3	3	3
P35579	MYH9_HUMAN	MYH9	Myosin-9	3	2	3	3	3	3	3	3	3	2	2
O14950	ML12B_HUMAN	MYL12B	Myosin regulatory light chain 12B	4	3	4	4	3	4	4	4	3	3	3
P60660	MYL6_HUMAN	MYL6	Myosin light polypeptide 6	3	2	4	3	3	3	3	3	3	2	2
Q6ZSR9	YJ005_HUMAN	NA	Uncharacterized protein FLJ45252	NA	NA	NA	NA	NA	NA	NA	NA	NA	NA	NA
P16083	NQO2_HUMAN	NQO2	Ribosyldihydronicotinamide dehydrogenase [quinone]	3	2	3	3	2	3	3	3	3	3	3
O43809	CPSF5_HUMAN	NUDT21	Cleavage and polyadenylation specificity factor subunit 5	3	2	3	3	3	3	3	3	3	3	3
Q9UKK9	NUDT5_HUMAN	NUDT5	ADP-sugar pyrophosphatase	3	2	3	3	3	4	4	3	3	3	3
P13674	P4HA1_HUMAN	P4HA1	Prolyl 4-hydroxylase subunit alpha-1	3	3	4	3	2	3	3	4	3	3	4
Q9NWQ8	PAG1_HUMAN	PAG1	Phosphoprotein associated with glycosphingolipid-enriched microdomains 1	NA	NA	NA	NA	NA	NA	NA	NA	NA	NA	NA
Q9HBI0	PARVG_HUMAN	PARVG	Gamma-parvin	4	4	4	4	3	4	4	4	3	4	4
Q9BUL8	PDC10_HUMAN	PDCD10	Programmed cell death protein 10	3	2	4	3	3	3	3	4	3	2	3
P09619	PGFRB_HUMAN	PDGFRB	Beta-type platelet-derived growth factor receptor	3	3	3	4	2	3	3	3	3	2	2
Q99623	PHB2_HUMAN	PHB2	Prohibitin-2	2	2	3	3	2	2	3	3	2	3	3
Q99640	PMYT1_HUMAN	PKMYT1	Membrane-associated tyrosine- and threonine-specific cdc2-inhibitory kinase	3	3	3	2	2	3	4	3	3	3	3
A5A3E0	POTEF_HUMAN	POTEF	POTE ankyrin domain family member F	NA	NA	NA	NA	NA	NA	NA	NA	NA	NA	NA
Q06203	PUR1_HUMAN	PPAT	Amidophosphoribosyltransferase	NA	NA	NA	NA	NA	NA	NA	NA	NA	NA	NA
P23284	PPIB_HUMAN	PPIB	Peptidyl-prolyl cis-trans isomerase B	3	2	3	3	3	3	3	3	3	3	3
Q13131	AAPK1_HUMAN	PRKAA1	5'-AMP-activated protein kinase catalytic subunit alpha-1	3	2	3	3	2	3	3	4	3	3	3
Q05397	FAK1_HUMAN	PTK2	Focal adhesion kinase 1	3	2	3	3	2	3	3	3	2	2	2
Q14289	FAK2_HUMAN	PTK2B	Protein-tyrosine kinase 2-beta	3	3	3	4	2	3	3	4	2	2	2
Q96D71	REPS1_HUMAN	REPS1	RalBP1-associated Eps domain-containing protein 1	3	3	4	4	3	4	4	4	3	3	1
O43353	RIPK2_HUMAN	RIPK2	Receptor-interacting serine/threonine-protein kinase 2	2	2	3	3	2	3	3	3	2	2	3
Q15404	RSU1_HUMAN	RSU1	Ras suppressor protein 1	3	3	3	4	2	3	4	4	3	3	3
P31947	1433S_HUMAN	SFN	14-3-3 protein sigma	2	2	3	3	2	2	3	3	3	2	3
Q86YV5	SG223_HUMAN	SGK223	Tyrosine-protein kinase SgK223	3	3	4	4	3	3	4	4	4	3	4
Q9H0K1	SIK2_HUMAN	SIK2	Serine/threonine-protein kinase SIK2	3	3	4	3	3	4	4	4	3	3	3
Q9Y2K2	SIK3_HUMAN	SIK3	Serine/threonine-protein kinase SIK3	3	3	4	4	3	3	4	4	3	3	3
Q9H2G2	SLK_HUMAN	SLK	STE20-like serine/threonine-protein kinase	3	2	4	3	2	3	4	4	3	3	3
P12931	SRC_HUMAN	SRC	Proto-oncogene tyrosine-protein kinase Src	3	2	2	3	2	2	2	3	1	2	2
O94804	STK10_HUMAN	STK10	Serine/threonine-protein kinase 10	NA	NA	NA	NA	NA	NA	NA	NA	NA	NA	NA
Q9Y6E0	STK24_HUMAN	STK24	Serine/threonine-protein kinase 24	3	2	3	3	2	3	3	3	3	3	3
Q7RTN6	STRAA_HUMAN	STRADA	STE20-related kinase adapter protein alpha	3	3	4	4	3	4	4	4	4	4	3
P53999	TCP4_HUMAN	SUB1	Activated RNA polymerase II transcriptional coactivator p15	3	2	3	3	2	2	2	3	3	3	3
Q9UHD2	TBK1_HUMAN	TBK1	Serine/threonine-protein kinase TBK1	3	2	3	2	2	3	3	3	3	2	3
P42680	TEC_HUMAN	TEC	Tyrosine-protein kinase Tec	3	3	3	3	2	3	3	3	3	3	2
Q15569	TESK1_HUMAN	TESK1	Dual specificity testis-specific protein kinase 1	3	3	4	4	3	3	4	4	3	3	3
Q96S53	TESK2_HUMAN	TESK2	Dual specificity testis-specific protein kinase 2	3	3	4	4	3	3	4	4	3	3	3
Q71U36	TBA1A_HUMAN	TUBA1A	Tubulin alpha-1A chain	2	1	3	3	2	3	3	3	3	3	3
P68363	TBA1B_HUMAN	TUBA1B	Tubulin alpha-1B chain	3	2	3	3	2	3	3	4	3	2	2
Q9BQE3	TBA1C_HUMAN	TUBA1C	Tubulin alpha-1C chain	3	1	3	3	2	3	3	3	3	3	3
P49411	EFTU_HUMAN	TUFM	Elongation factor Tu, mitochondrial	3	2	3	3	2	3	3	3	3	2	2
P29597	TYK2_HUMAN	TYK2	Non-receptor tyrosine-protein kinase TYK2	3	3	3	4	2	3	3	3	3	2	2
P26368	U2AF2_HUMAN	U2AF2	Splicing factor U2AF 65 kDa subunit	3	2	3	3	3	3	3	3	2	3	3
Q8TF42	UBS3B_HUMAN	UBASH3B	Ubiquitin-associated and SH3 domain-containing protein B	3	3	3	3	3	3	3	4	3	2	1
Q6PHR2	ULK3_HUMAN	ULK3	Serine/threonine-protein kinase ULK3	NA	NA	NA	NA	NA	NA	NA	NA	NA	NA	NA
Q13432	U119A_HUMAN	UNC119	Protein unc-119 homolog A	2	2	3	3	2	3	3	3	2	2	3
P07947	YES_HUMAN	YES1	Proto-oncogene tyrosine-protein kinase Yes	3	3	3	4	2	3	3	4	2	3	3
P31946	1433B_HUMAN	YWHAB	14-3-3 protein beta/alpha	2	2	3	3	2	2	3	3	2	2	2
P62258	1433E_HUMAN	YWHAE	14-3-3 protein epsilon	2	2	3	3	2	2	3	3	3	2	3
P61981	1433G_HUMAN	YWHAG	14-3-3 protein gamma	2	2	3	2	2	2	3	3	2	2	2
Q04917	1433F_HUMAN	YWHAH	14-3-3 protein eta	2	2	3	3	2	2	3	3	3	2	3
P27348	1433T_HUMAN	YWHAQ	14-3-3 protein theta	2	2	2	3	2	3	3	3	2	2	2
P63104	1433Z_HUMAN	YWHAZ	14-3-3 protein zeta/delta	2	2	3	3	2	2	3	3	2	2	2

	mRNA levels relative to ABL			
**Patient**	**BTK**	**LYN**	**ILK**	**TEC**
1	6.68	3.61	4.40	0.51
2	6.44	1.35	6.77	0.39
3	5.15	1.86	11.67	0.24
4	11.55	6.63	5.48	1.51
5	8.22	1.57	17.98	0.37
6	5.37	3.42	8.83	0.25

	mRNA levels relative to ABL			
**Cell Line**	**BTK**	**LYN**	**ILK**	**TEC**
BV-173	1.57	1.24	0.45	0.01
Z-119	11.52	7.74	2.85	0.42
SUP-B15	3.52	1.03	2.55	0.17
