# Supplementary material for: A Target-Disease Network Model of Second-Generation BCR-ABL Inhibitor Action in Ph+ ALL
Source: PLoS One. 2013 Oct 10;8(10):e77155. doi: 10.1371/journal.pone.0077155 (PMC3795025; doi:10.1371/journal.pone.0077155)
Supplement: File S2 — Figure S1. The example of how a single seed diffuses information towards its neighbors. Figure S2. Three variants of a random walk. Figure S3. Computation of the disease P-values. Table S1. Disease network model P-values sorted in ascending order for dasatinib. (PDF) [file pone.0077155.s002.pdf]

Supplementary Methods

A target-disease network model of second-generation BCR-ABL inhibitor action in Ph+ ALL

Uwe Rix[1,*,¶], Jacques Colinge[1,*], Katharina Blatt[2], Manuela Gridling[1], Lily L. Remsing Rix,[1] Katja Parapatics[1], Sabine Cerny-Reiterer[2,3], Thomas R. Burkard[1#], Ulrich Jäger[2], Junia V. Melo[4], Keiryn L. Bennett[1], Peter Valent[2,3] and Giulio Superti-Furga[1]

[1]CeMM – Research Center for Molecular Medicine of the Austrian Academy of Sciences, Vienna, Austria; [2]Department of Internal Medicine I, Division of Hematology and Hemostaseology, Medical University of Vienna, Vienna, Austria; [3]Ludwig Boltzmann Cluster Oncology, Vienna, Austria; [4]Department of Haematology, SA Pathology Centre for Cancer Biology, Adelaide, Australia

*These two authors contributed equally

¶Current address: H. Lee Moffitt Cancer Center & Research Institute, Tampa, FL, USA

#Current address: IMBA - Institute for Molecular Biotechnology of the Austrian Academy of Sciences, Vienna, Austria

Motivating diffusion processes

The most common diffusion process introduced on a network – or a graph – for the purpose of scoring the relatedness of all the network nodes with a set of seed nodes representing disease genes is a random walk with restart[1,2,3,4]. The motivation is to extend the influence of a given set of seed nodes, e.g. disease causing genes, to the entire network to associate each node with a score that measures its relevance with respect to the *perturbation* represented by the seed nodes.

Naively, directly adjacent nodes could be chosen but it would ignore long range interactions and if a seed node is a hub and has a large number of neighbors, it is questionable whether it would impact all its neighbors as strongly as another less connected seed. Considering adding a second layer of adjacent nodes (the neighbors of the seed neighbors) to capture long range influences is usually meaningless since the topology of the human PPI network (scale-free) is such that a gigantic number are then selected and the specificity of the seed nodes lost completely. These considerations motivated the use of global methods able to take the whole network topology into account to both measure longer range synergies between seed nodes and address the large number of neighbor issue by reducing the impact on neighbors in such cases.

A random walk is a diffusion process that naturally implements the above concepts of global topology consideration and reduction of highly connected node influence on their neighbors, See Fig. 4A, S1 in this file and S2A in this file. Hereafter, we introduce two additional variants of the regular random walk that have alternative and potentially relevant interpretation of how the influence of seed nodes diffuses in a biological network.

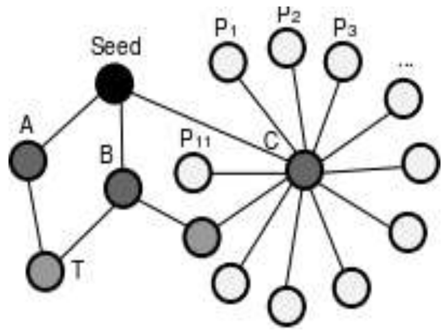

Figure S1. The example of how a single seed diffuses information towards its neighbors. A, B, and C are strongly influenced as they are immediate neighbors of the seed and the seed does not have a large number of neighbors. The neighbors of C receive little influence due to their number, except the one shared with B that benefits from a synergy between B and C. The same is true for T, which is shared by A and B and thus, although it is a second interactor of the seed, gets more influence than the unique neighbors of C. When multiple seeds exist, their combined influence works in a similar fashion as in the synergistic influence of A and B on T for instance.

Different diffusion processes

In the case of a random walk, the matrix P of the iteration

$$x_{i+1}=(1-\alpha)Px_i+\alpha x_0 \quad (1)$$

is obtained by computing the transpose of the row normalized adjacency matrix of the PPI network. Namely, let the PPI network be represented by the graph $G=(V,E)$, V the nodes (vertices) and E the edges (the protein-protein interactions). The adjacency matrix A of the graph G is a binary matrix with a_{ij}=1 if and only if the nodes v_i and v_j are connected by an edge $e_{ij} \in E$. The row normalized adjacency matrix, which we denote B, is obtained by modifying A such that the sum of all the elements of one row is equal to 1:

$$b_{jk} = \frac{a_{jk}}{\sum_i a_{ji}}$$

and

$$P=B^{\mathrm{T}}. \quad (2)$$

In their paper, Köhler et al.[1] even studied more general diffusion kernel and observed no advantage over the random walk with restart defined by Eq. (1) and (2). We did not repeat this study and only considered the random walk with restart. The choice of the restart parameter α has also been shown to have very limited impact on the results[5], an observation we also made (data not shown). How the random walk process diffuses a score present at a given node to neighbors is illustrated in Fig. S2A in this file and we observe that a node i diffuses its current score to its neighbors in identical proportions equal to 1/number of neighbors.

In the regular random walk obtained with $P=B^{\mathrm{T}}$, the diffusion depends on the number of edges of a given node to distribute its score – or probability – to its neighbors. Another natural alternative is to say that the "receiving" nodes get a score from each of their neighbors according to the number of their neighbors, i.e. the same logic is applied from the perspective of the target node instead of the source node, see Fig. S2B in this file. Biologically, one can argue that a protein regulated by many others is less influenced by each of its regulators compared to a protein regulated by a few others. The P matrix is computed as follows:

$$N_k = \sum_j a_{kj}, \quad (3)$$

$$p_{jk} = \frac{\frac{1}{N_k}}{\sum_i \frac{1}{N_i} a_{ji}}. \quad (4)$$

A natural alternative to the regular random walk above is not to transpose B, i.e. to use

$$P=B. \quad (5)$$

The consequence on the diffusion is illustrated in Fig. S2C in this file and we observe that in strings of nodes the score diffusing from one seed node is less reduced, which might be a better model for capturing signaling cascades. Another consequence of Eq. (5) is that $\sum_i x_i^\infty \neq 1$, whereas for Eq. (2) the asymptotic score distribution sums up to unity since it is a Markov chain.

Convergence of the iteration

The convergence of (1) can be proved for all 3 variants above at once. We have:

$$x_1 = (1-\alpha)Px_0 + \alpha x_0,$$
$$x_2 = (1-\alpha)^2P^2x_0 + \alpha(1-\alpha)Px_0 + \alpha x_0$$

and hence by induction

$$x_m = [(1-\alpha)^m P^m + \alpha \textstyle\sum_{i=0}^{m-1}(1-\alpha)^i P^i]x_0. \qquad (6)$$

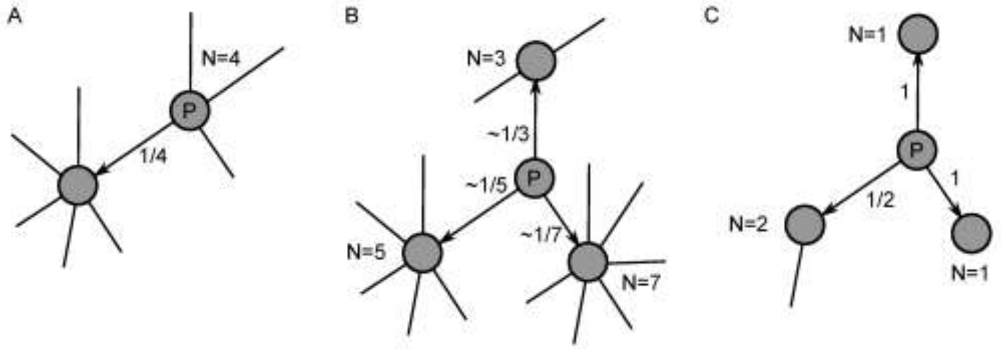

Figure S2. Three variants of a random walk. (A) The regular random walk, where at every iteration the probability to move from node P to another node is equal to 1/degree(P). (B) A modified version where the transition probabilities from node P are proportional to the degree of each connected node (sum of transition probabilities is 1). (C) A diffusion process that is no longer a true Markov chain and where the transition probabilities are equal to the 1/degree of the target nodes but with summing to one. Comparing A, B, and C, we see that they model “signal transmission” differently. A means that a hub does not influence its neighbors strongly, which is likely to be wrong. B means that highly connected (regulated) proteins are more difficult to influence, which is more plausible. C has some potential to send a signal without excessive attenuation, which also provides a plausible model and – in our hands – gave better results repurposing compounds, e.g. as illustrated in Table S1in this file.

Now, since all norms are equivalent in a finite dimensional real-valued vector space, we chose the infinite norm and obtain

$$\|x_{m+k} - x_m\| = \left\|\left[(1-\alpha)^{m+k}P^{m+k} - (1-\alpha)^m P^m + \alpha \sum_{i=m}^{m+k-1}(1-\alpha)^i P^i\right]x_0\right\|$$
$$\leq \left[(1-\alpha)^{m+k}\|P\|^{m+k} + (1-\alpha)^m\|P\|^m + \alpha \textstyle\sum_{i=m}^{m+k-1}(1-\alpha)^i\|P\|^i\right]\|x_0\|. \qquad (7)$$

For the infinite norm, $\|P\| \leq 1$ (for each 3 variants of the iteration) and $\|x_0\| \leq 1$. Therefore

$$(7) \leq 2(1-\alpha)^m + \alpha(1-\alpha)^m \frac{1-(1-\alpha)^k}{1-(1-\alpha)} \leq 3(1-\alpha)^m, \qquad (8)$$

which can be made arbitrarily small provided m is large enough and $\alpha \in]0;1[$, i.e. proving that the sequence x_i is Cauchy and thus convergent.

Diffusion process used in the manuscript

To analyze the kinase inhibitor target profiles discussed in the paper we selected the variant without transpose ($P{=}B$) since we have observed that it ranks disease network models against a drug treatment network model better (known application areas ranked higher), see hereunder.

By comparing each drug treatment network model against a set of diseases [1] distinct from Ph+ ALL, we obtained for each compound a null distribution used to estimated P-values for the relevant cell lines, see Figure S3 in this file. In Table S1 in this file, we report the best 20 P-values for the Ph+ ALL cell lines, the Köhler et al. list, and two CML models [4] we added (with and without LYN) to cover the most prominent area of application of the four compounds (CML). The “LYN-resistant” patient model achieves a better P-value for these second generation BCR-ABL inhibitors, except in the case of nilotinib that does not perform well in general. We note the prevalence of cancers at the top of the list, which makes sense, with some known case of drug efficacy [6] and plausible new indications, e.g. Noonan syndrome where other kinase inhibitors are in clinical trials.

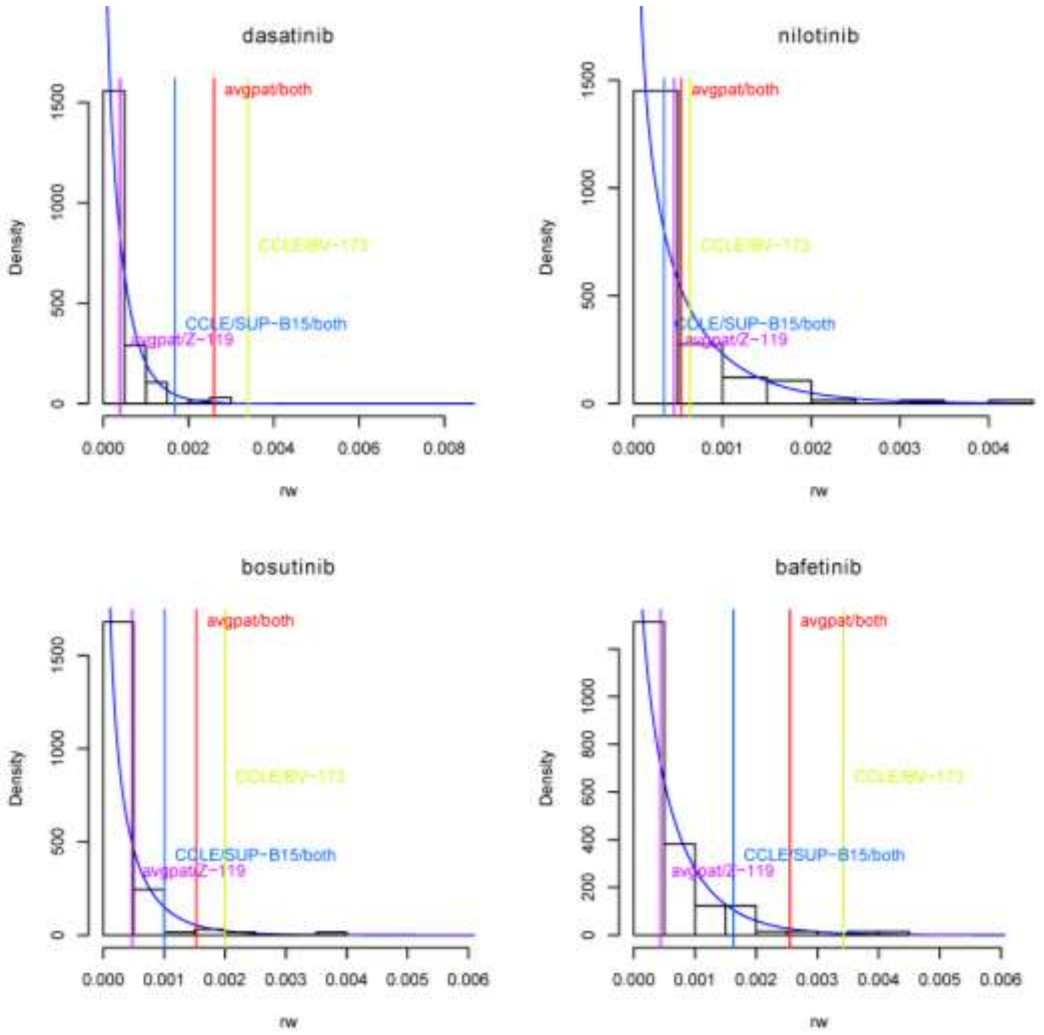

Figure S3. Computation of the P-values. A Gamma distribution (blue solid line) is fit on the non related disease models of the Köhler list (histogram) to determine the P-values of the 5 relevant cell lines.

Table S1. Disease network model P-values sorted in ascending order for dasatinib (first 20 entries). Significant P-values (<5%) are in red.

Disease	dasa.pval	nilo.pval	bosu.pval	bafe.pval
CML (LYN)	7.63E-09	1.27E-01	1.30E-05	1.15E-04
CML	5.66E-05	3.00E-01	3.58E-03	2.95E-03
CCLE/BV-173	5.34E-04	2.69E-01	1.27E-02	5.03E-03
Lung cancer	1.64E-03	4.87E-03	6.54E-03	3.50E-03
avgpat/both	2.92E-03	3.17E-01	2.93E-02	1.80E-02
Noonan Syndrome, Costello syndrome, Cardiofaciocutaneous Syndrome (Noonan Syndrome)	3.38E-03	8.06E-02	2.02E-02	1.84E-02
Hepataocellular carcinoma	5.23E-03	1.56E-03	2.24E-02	2.08E-03
CCLE/SUP-B15/both	2.07E-02	4.42E-01	7.74E-02	7.03E-02
Breast cancer familial (Familial breast cancer (somatic mutation))	5.11E-02	2.74E-02	9.28E-02	3.56E-02
Atypical mycobacteriosis, familial	5.85E-02	6.19E-02	5.67E-02	5.33E-02
Medulloblastoma	5.88E-02	6.50E-02	9.85E-02	6.00E-02
Juvenile myelomonocytic leukemia	6.69E-02	2.35E-01	1.14E-01	1.21E-01
Elliptocytosis	7.80E-02	2.39E-01	1.42E-01	2.47E-01
Systemic lupus erythematodes (Susceptibility to SLE)	8.43E-02	5.12E-01	1.17E-01	1.80E-01
Glioma of brain, familial (Hereditary disorders characterized by occurence of glioma and other disorders)	8.63E-02	5.71E-02	1.17E-01	6.09E-02
Pheochromocytoma (Somatic mutations associated with isolated pheochromocytoma)	1.03E-01	5.08E-02	1.47E-01	4.33E-02
Pancreatic carcinoma	1.05E-01	4.70E-02	1.32E-01	5.99E-02
Esophageal carcinoma	1.08E-01	5.82E-02	1.49E-01	6.91E-02

References

1. Kohler S, Bauer S, Horn D, Robinson PN (2008) Walking the interactome for prioritization of candidate disease genes. Am J Hum Genet 82: 949-958.
2. Berger SI, Ma'ayan A, Iyengar R (2010) Systems pharmacology of arrhythmias. Sci Signal 3: ra30.
3. Colinge J, Rix U, Bennett KL, Superti-Furga G (2012) Systems biology analysis of protein-drug interactions. Proteomics Clin Appl 6: 102-116.
4. Colinge J, Rix U, Superti-Furga G (2010) Novel global network scores to analyze kinase inhibitor profiles. In: Chen L, Zhang X, Shen B, Wu L, Wang Y, editors. 4th International conference on computational systems biology. Suzhou, China: World Publishing Company. pp. 305-313.
5. Chen J, Xu H, Aronow BJ, Jegga AG (2007) Improved human disease candidate gene prioritization using mouse phenotype. BMC Bioinformatics 8: 392.
6. Li J, Rix U, Fang B, Bai Y, Edwards A, et al. (2010) A chemical and phosphoproteomic characterization of dasatinib action in lung cancer. Nat Chem Biol 6: 291-299.
